# Gene Discovery through Transcriptome Sequencing for the Invasive Mussel *Limnoperna fortunei*

**DOI:** 10.1371/journal.pone.0102973

**Published:** 2014-07-21

**Authors:** Marcela Uliano-Silva, Juliana Alves Americo, Rodrigo Brindeiro, Francesco Dondero, Francisco Prosdocimi, Mauro de Freitas Rebelo

**Affiliations:** 1 Carlos Chagas Filho Biophysics Institute, UFRJ, Rio de Janeiro, Brazil; 2 Biology Institute, UFRJ, Rio de Janeiro, Brazil; 3 Departmenf of Science and Technological Innovation, Universitá del Piemonte Orientale, Alessandra, Italy; 4 Medical Biochemistry Institute, UFRJ, Rio de Janeiro, Brazil; Universidade Federal do Rio de Janeiro, Brazil

## Abstract

The success of the Asian bivalve *Limnoperna fortunei* as an invader in South America is related to its high acclimation capability. It can inhabit waters with a wide range of temperatures and salinity and handle long-term periods of air exposure. We describe the transcriptome of *L. fortunei* aiming to give a first insight into the phenotypic plasticity that allows non-native taxa to become established and widespread. We sequenced 95,219 reads from five main tissues of the mussel *L. fortunei* using Roche’s 454 and assembled them to form a set of 84,063 unigenes (contigs and singletons) representing partial or complete gene sequences. We annotated 24,816 unigenes using a BLAST sequence similarity search against a NCBI nr database. Unigenes were divided into 20 eggNOG functional categories and 292 KEGG metabolic pathways. From the total unigenes, 1,351 represented putative full-length genes of which 73.2% were functionally annotated. We described the first partial and complete gene sequences in order to start understanding bivalve invasiveness. An expansion of the hsp70 gene family, seen also in other bivalves, is present in *L. fortunei* and could be involved in its adaptation to extreme environments, e.g. during intertidal periods. The presence of toll-like receptors gives a first insight into an immune system that could be more complex than previously assumed and may be involved in the prevention of disease and extinction when population densities are high. Finally, the apparent lack of special adaptations to extremely low O_2_ levels is a target worth pursuing for the development of a molecular control approach.

## Introduction

Only a small fraction of non-native taxa arriving in a new environment can establish populations and become widespread plagues [1], and their invasiveness can be related to phenotypic plasticity [2]. Bivalves are one of the oldest and most widespread groups of invertebrates [3] and it is therefore not surprising that this taxon contains some of the most aggressive invasive species. Two notorious examples are the Golden Mussel *Limnoperna fortunei* in South America and the Zebra Mussel *Dreissena polymorpha* in North America and Europe [4].

Since its arrival in Argentina through ballast water of ships coming from Asia in 1991 [5] *L. fortunei* has dispersed more than 5,000 km upstream and is now in dangerous proximity to the Amazon River basin. With high filter-feeding and reproductive rates [6], tolerance to a wide range of environmental parameters [7] and the ability to attach to almost any surface, it can reach dramatic densities of 150,000 ind.m^−2^ over a year [9]. It has colonized lotic and lentic environments as diverse in limnological characteristics as the La Plata river estuary and the Pantanal wetlands. The mussel’s ability to harm the environment has earned it the epithet ‘ecosystem engineer’ (for a review refers to [8]).

So far, there is no consensual approach to control the spread of invasive bivalves. Chlorine is still routinely employed in industrial facilities to kill bivalve larvae and adults, in spite of its inefficiency and environmental hazard. Attempts to control infestation using a wide variety of chemical substances have failed due to *L. fortunei*’s high tolerance and resistance [10]. In the field, only the drastic reduction in water depth and dissolved oxygen resulting from the flood rhythm in the Brazilian Pantanal watershed (a phenomenon regionally known as ‘dequada’) seems to be able to annihilate mussels, decreasing the populations and controlling the dispersion up to the Amazon river basin [11].

Here we investigate *L. fortunei’s* transcriptome to bring insights of genes and pathways that could be related to its success as an invader: genes involved in high filtering rates and in the production of a strong byssus; antioxidant enzymes and chaperones involved in the ability to inhabit waters with wide variations of dissolved oxygen and temperature. The recently sequenced genome of the oyster *Crassostrea gigas* [12] has uncovered an unusually high copy number of genes related to gene-environment relationships in bivalves, such as heat shock proteins and cytochrome P450, that are highly regulated under environmental stress.

In this study, we performed a *de* *novo* transcriptome sequencing of the Golden Mussel *L. fortunei* on a normalized library of expressed transcripts from five main tissues aiming to discover gene sequences that could enable us to study *L. fortunei*’s invasive abilities at a molecular level. We provide valuable information to detect specific targets for future approaches to control infestation in industrial plants and prevent dispersion in the delicate South America’s continental waters.

## Results

### 2.1. Sequencing analysis

The normalized cDNA library of *L. fortunei* was submitted to two full plate 454 GS Jr (Basel, Switzerland) sequencing runs for a total of two independent reactions. After adaptor trimming, 91.65% of pyrosequencing data were used for further analysis (Table 1). Sequencing reads were deposited in the SRA database [13] (accession number SRR942484).

**Table 1:** Summary of the cleaning procedure for *L. fortunei* transcriptomic data.

	TOTAL NUMBEROF READS	TOTAL NUMBEROF BASES	READS AVERAGESIZE	TOTAL NUMBEROF CLEAN READS	TOTAL NUMBEROF CLEAN BASES^a^	CLEAN READSAVERAGE SIZE^a^
**1^st^ run**	41,045	25,741,354	627	41,044	23,595,038	574
**2^nd^ run**	54,174	31,737,816	585	54,173	29,161,265	538
**Total**	95,219	57,479,170	603	95,217	52,756,303	554

a Crossmatch data, after cleaning.

### 2.2. Sequence assembly

Three different assembly algorithms, CAP3, MIRA and Newbler, were tested on *L. fortunei* transcriptome data and were evaluated using BLAST annotation, and Internal and External Consistency Indexes – ICI/ECI (Table 2; Methods 5.5). The 4,605 contigs were gather with the 79,478 singletons to form the set of 84,063 unigenes (Table 2). Contigs consisted in the longer length consensus sequences formed from overlapping transcripts during clusterization process. Singletons are single transcripts that did not overlap with any other during clusterization.

**Table 2:** A summary of the Sequence clustering analysis.

ASSEMBLYALGORITHM	NUMBER OFSEQUENCESCLUSTERED	NUMBER OFCONTIGS	AVERAGECONTIGSSIZE	NUMBER OFUNIGENES^a^	AVERAGEUNIGENESSIZE	AVERAGEICI SCORE	AVERAGEECI SCORE	UNIGENESANNOTATED
**CAP3**	15,759	4,605	614	84,063	574	86.24	24.5	17,174
**Newbler**	41,474	2,395	504	52,268	267	83.90	39.2	10,303
**MIRA**	21,366	7,343	596	81,195	498	74.12	15.5	16,475

a Contigs and singletons.

ICI pointed to CAP3 (86.24) as the best assembly method. All three algorithms computed a satisfactory average ECI distance between consensus pairs although values for CAP3 (24.5) were 62% and for MIRA (15.5) were 39% smaller than Newbler’s (39.2). Finally, the highest number of annotated sequences were found for contigs assembled by CAP3 which were annotated by BLAST (e-valeu cutoff < = 10^−5^) against UNIPROT database (Table 2).

The evaluation using the three metrics led to the choice of the CAP3 assembly dataset, using singletons and contigs, for downstream analysis. This Transcriptome Shotgun Assembly project has been deposited at DDBJ/EMBL/GenBank under the accession GBGC00000000. The version described in this paper is the first version, GBGC01000000. Unigenes are also available for download at http://goo.gl/DZt7K8 (including sequences from Table S6).

### 2.3. Analysis of unigenes assembled by CAP3

**Figure 1:**
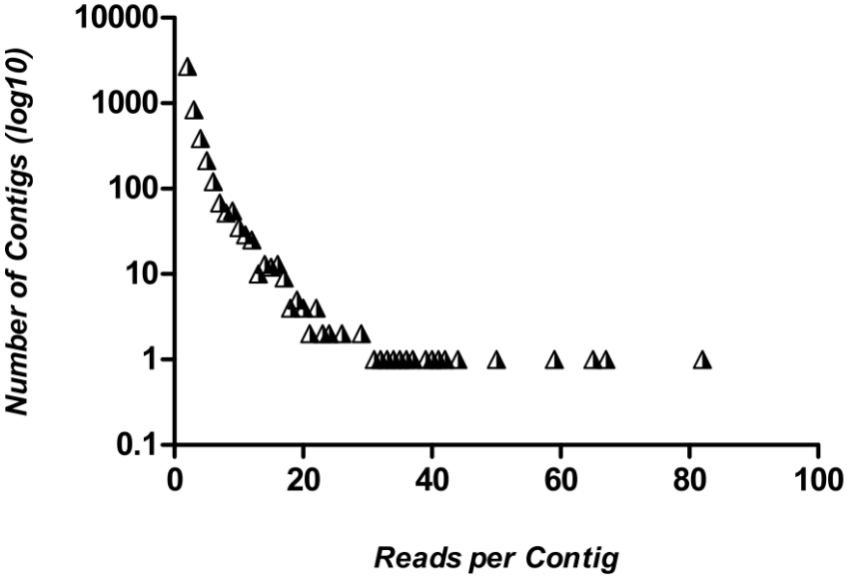
Quantity of contigs by number of reads assembled. Most contigs (2,687, representing 58% of total) resulted from the assembly of two single reads. Only nine contigs were formed by 40 or more reads. Triangles represent *L. fortunei* contigs.

Figure 1 shows the number of reads used to build the different contigs. As expected in a well-normalized library, most contigs (2,687, representing 58% of the total) resulted from the assembly of two single reads. Only nine contigs were formed by 40 or more reads, including three full-length genes annotated as constitutive genes (e-value cutoff < = 10^−5^) by resemblance with other bivalve proteins: actin (EKC33605.1), nuclear autoantigenic sperm protein (EKC35856.1) and 60S ribosomal protein (ABA46793.1).

### 2.4. Sequence annotation

BLAST analysis returned significant similarities (e-value cutoff < = 10^−5^) with other protein-coding genes for a small fraction of assembled genes. Table 3 reports the results obtained against the three major protein databases, UNIPROT, NCBI non redundant (nr) and eggNOG. UNIPROT more frequent BLAST hits were against human sequences (16%), *Mus musculus* (15%), *Ratus novergicus* (9%) and *Drosophila melanogaster* (5%). The reproducibility of results in different databases highlights the robustness of the analysis. However, almost 71% of unigenes were not annotated in BLAST searches with protein sequences from the NCBI nr database (FASTA sequences and the annotation text file can be downloaded at http://goo.gl/DZt7K8). We identified several key genes for gene-environment relationships such as cytochrome P450, proteins involved in the toll-like receptors signaling pathway, glutathione S-transferase (GST), gamma-glutamil-transpeptidase (GGT) and adhesive proteins Mepf1 (9 unigenes) and Mepf2 (103 unigenes) (Table S2 in supporting information presents the KEGG Orthology identifiers (KO) and the unigenes IDs). For a more straightforward analysis, Table 4 shows the quantity of unigenes annotated as cellular defenses-related genes through BLAST searches against *C. gigas* protein sequences (Table S5 presents *L. fortunei* IDs for genes in Table 4).

**Table 3:** Functional annotation of the *L. fortunei* transcriptome.

Number of unigenes		No of non-annotated unigenes	% of annotated unigenes
**Total**	84,063	–	
**BLAST matches against NCBI nr**	24,816	59,247	29
**BLAST matches against UNIPROT**	17.174	66,889	20
**BLAST matches against eggNOG**	12,284	71,779	14

**Table 4:** Number of annotated transcripts related to cellular defense.

Gene family	
Catalase (CAT)	4
Superoxide dismutase (SOD)	20
Glutathione Reductase (GR)	10
Glutathione Peroxidase (GPx)	13
Thioredoxin (TRx)	85
Peroxiredoxin (PRx)	6
ABCs	38
γ-glutamil-transpeptidase (GGT)	2
Cytochrome P450 (CYP)	95

**Figure 2:**
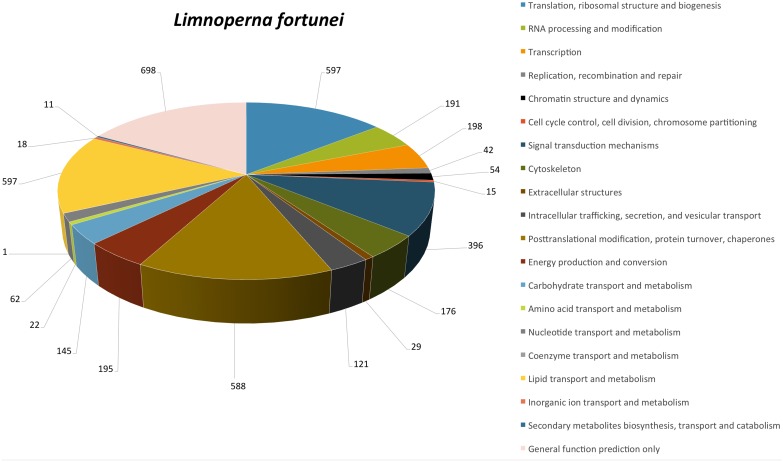
Functional eggNOG categories of *L. fortunei* unigenes.

A total of 3,612 *L. fortunei* unigenes were classified into 20 of 24 functional categories at eggNOG database (Figure 2).

KO identifiers were assigned to 2,579 unigenes, indicating involvement in 292 different pathways (Table S2). These sequences could be assigned to specific KEGG classes: 39.8% to genetic information processing (GIP); 34.3% to metabolism (with enzymes being the most abundant subcategory, 368 unigenes) and 11.5% to Signaling and cellular processes, including cytoskeleton proteins and transporters.

### 2.5. Transcriptome coverage and full-length identification

To estimate transcriptome coverage, we BLASTed *L. fortunei* unigenes against *C. gigas* predicted proteins [12], annotating 24,682 unigenes (29.4% of the total) against 12,682 out of 28,027 *C. gigas* protein.

This BLAST search also allowed us to estimate 1,351 full-length genes. A gene was considered to be complete when an *L. fortunei* unigene (assembled from the transcriptome described here) covered 90% or more of the sequence length in *C. gigas*. Of the full length genes, 990 were annotated by BLASTp searches against NCBI nr (Supporting Information Table S3).

### 2.6. Heat shock protein - hsp70 family in Mollusca

KEGG annotation showed 33 different heat shock proteins. This estimation increased to 55 different HSP70, among 127 unigenes, after BLASTing against the predicted proteins annotated in the *C. gigas* genome project [12]. The relevance of this gene family for gene environment relationships led us to further study the evolutionary divergence, conducting a phylogenetic analysis of all 179 known HSP70 proteins from 46 mollusk species.

**Figure 3:**
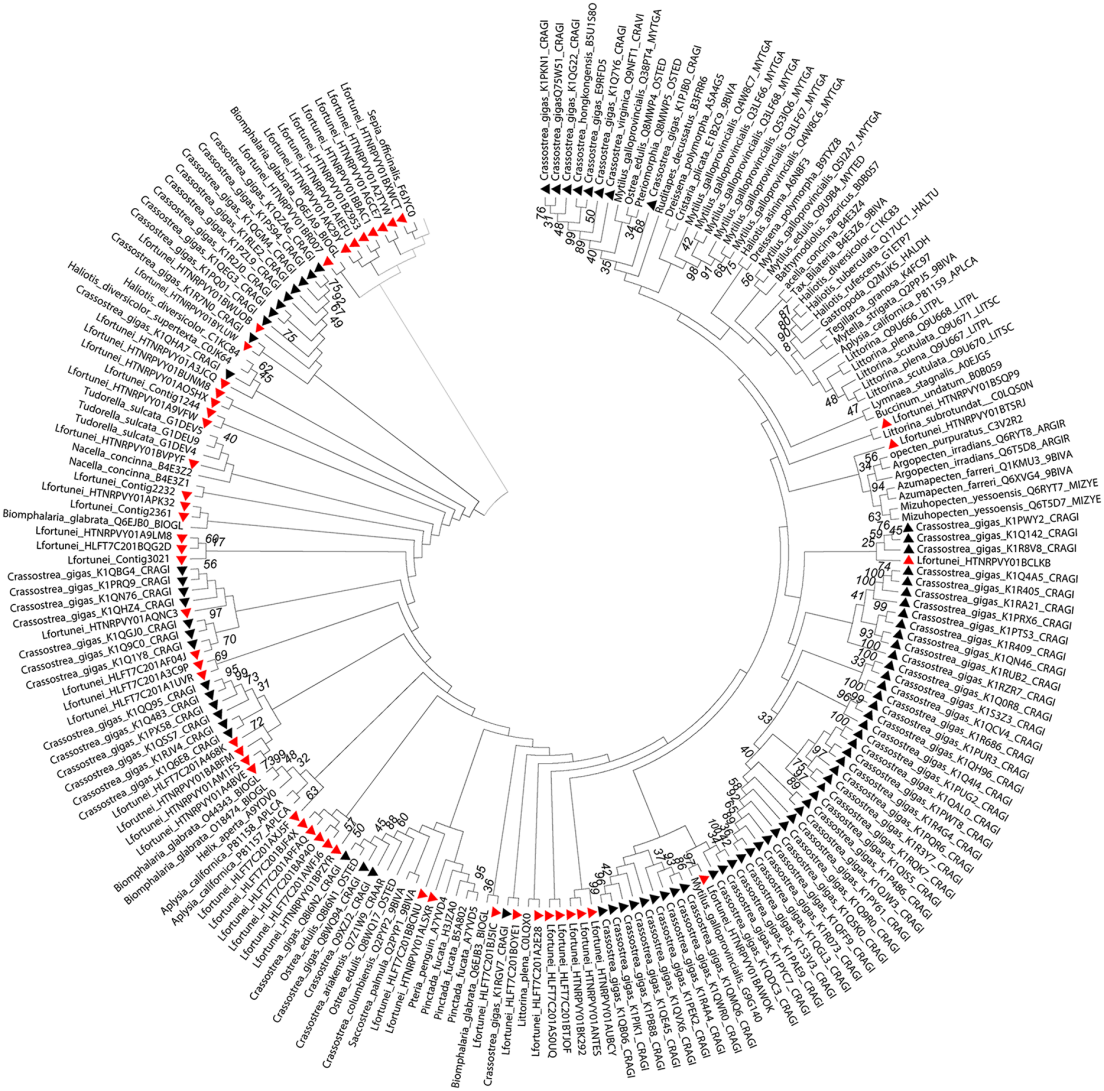
Consensus phylogenetic tree of molluscan hsp70 family members. *L. fortunei* sequences are marked with a red triangle and *C. gigas* sequences with a black triangle for comparison. The tree was constructed using the maximum likelihood method and bootstrapping (100 pseudoreplicates, values less than 30% are not shown).

Figure 3 shows the Maximum Likelihood tree (Methods 5.9) whose robustness was assessed using bootstrapping (100 pseudo-replicates).

While most of *L. fortunei*’s HSP70 isoforms were phylogenetically related to several different HSP70s of mollusks, two of them (HTNRPVY01BCLKB and HTNRPVY01BAWOK) clustered within a *C. gigas* expansion characterized by Zhang et al [12].

### 2.7. SSR identification

Of a total of 37,062 low complexity sequence regions or simple sequence repeats - SSRs - 19,776 were identified within unigenes, 3,378 (17%) of which were annotated by BLAST sequence similarity searches and can be considered as priority candidates for further development as genetic markers (Supporting information S1). The most frequent repeat motifs were tetranucleotides, which accounted for 35.68% of all SSRs, followed by pentanucleotides (30.75%), trinucleotides (16.3%), hexanucleotides (13.37%) and dinucleotides (3.98%). Among dinucleotides, AT motifs (49.7%) were the most abundant followed by AG (42.2%); among trinucleotide repeats, ACT motifs (39.6%) were the most abundant followed by ACG motifs (22%). The most abundant tetranucleotide motif was ACGT (76.9%). The most abundant penta- and hexanucleotide motifs were AACGT (57%) and AAACGT (28.3%), respectively.

## Discussion

In our study we assembled and annotated the first *de* *novo* transcriptome for the invasive species *L. fortunei*, which will allow the study of acclimation capacities involved in occupying new environments. The 24,816 annotated sequences and 1,351 full-length genes represent a rich source for gene discovery and invasiveness research. Our study has more than doubled the amount of information available in online databases, as of June 2013, for Mytilidae, which highlights the power of NGS technologies to enable molecular studies of non-model organisms [14].

Almost 70% of *L. fortunei* unigenes described here could not be functionally annotated. There is an obvious lack of protein and nucleotide sequences for related species in various databases: to date, only 1.5% of sequences present at NCBI nr belong to Mollusca, with only 0.5% from bivalves. Moreover, some unigenes were too short (574 bp) for significant alignments with characterized sequences [15]. Furthermore, taxonomically-restricted genes (TRGs) that allow species to adapt to specific ecological niches [16] usually account for 10 to 20% of total genes, with no resemblance in other species. The annotation generally tends to improve as more studies investigating the molecular mechanisms of mollusks are being developed [17], [18], [19].

The lack of a reference genome to allow a reads-mapping process requires special attention to the *de* *novo* assembly process [20], a complex decision that usually depends on data input [21], [22]. The high ICI scores found in this study suggest the reliability of all three assemble algorithms tested. CAP3 was chosen due to the highest ICI score and the larger number of unigenes with hits against sequences at the UNIPROT database.

### 3.1. Low complexity regions, SSRs

A large number of different low complexity regions known as simple sequence repeats (SSRs) were identified with the SciRoko software in the assembled *L. fortunei* transcriptome.

Such regions can be microsatellites [23], [24] but they may result from errors in sequencing homopolymeric regions: (1) over- and undercall, caused by longer (or shorter) homopolymer detection in the 454 light detection system [25], [26], (2) the carry forward and incomplete extension (CAFIE) caused by a few copies that grow in de-synchronization with the template, producing hybrid (of different sizes) or smaller reads [25], and (3) insertions followed by deletions (or vice versa) that create mismatches in sequence alignments [27].

Taking these possibilities into account, we suggest functionally annotated SSR-containing unigenes (Table S1) to be the best microsatellite candidates for *L. fortunei*, because of the unlikelihood of annotating a random sequence error.

### 3.2. The role of HSP70 in acclimation robustness

*Limnoperna fortunei* is notorious for its ability to tolerate a wide range of abiotic parameters such as water temperature (8–39°C) and salinity (up to 12 ppm) [8]. The same physiological robustness was described by Zhang *et al.* (2012) [12] in the pacific oyster *C. gigas* that they related to a high copy number of some important stress genes. In particular, the heat shock protein HSP70 was represented by 88 copies in the genome, a surprisingly high number when compared to 39 copies in sea urchins and 17 copies in humans.

Zhang *et al*. (2012) [12] published the only complete genome for bivalves so far. Because of the completeness of their approach it is relevant for a better understanding of *L. fortunei’s* biology.

In the phylogenetic tree for mollusca described by Zhang *et al* (2012–Supporting Information), most of the *C. gigas* HSP70 sequences are grouped in a separate expansion. According to the authors, this expansion suggests an adaptation to the stress of bivalve life history and in fact they found that the expression of all hsp70 increased at least 15-fold when oysters were exposed to air, high/low temperature or heavy metals. The expression of five of these genes (CGI10002823, CGI10003417, CGI10010647, CGI10002594, CGI10010646) increased 2,000 fold after exposure to air and high temperatures. With three of these HSP70 present inside the new gene expansion [12] its importance for the acclimatization to harsh environments seems very likely.

The resulting maximum likelihood tree resembles the one built by Zang *et al* ([12] in Supplementary Information). Some of the nodes do not have bootstrap support, which is a common problem when constructing phylogenetic trees of large gene families. Also, we have only partial sequences for a number of *L. fortunei* hsp70. The two unigenes of *L. fortunei* clustered within the *C. gigas* expansion (Results 2.6) suggest that this hsp70 expansion is present in the Golden Mussel as well. Other unigenes of *L. fortunei* are dispersed throughout the phylogenetic tree.

Under the assumption that the presence of the hsp70 gene expansion and the strong expression of its genes is the key to homeostasis maintenance of *C. gigas* under heavy stress conditions of estuarine environments, we might speculate that a similar hsp70 gene expansion provides *L. fortunei* the ability to invade and become established in new, challenging environments. In fact, the importance of HSP chaperones in invasiveness was also proposed for the recent introduction and establishment of *Mytilus gallopronvincialis* over *Mytilus trossulus* in North America [37]. Comparative transcriptomics [38] and proteomics [39] showed dramatically higher expression levels of the small heat-shock protein HSP24 in *M. galloprovincialis* compared to *M. trossulus* under heat stress. Evans and Hofmann [37] even suggest that the characterization of HSP gene expression pattern may help to predict a species invasive success [37].

### 3.3. Gene discovery for invasion control

As invasiveness potential and acclimation robustness seem to be related to phenotypic plasticity, the detection of key gene sequences could be the first step towards reliable predictive models to help control and fight invasive species.

At least three important properties of *L. fortunei* can be directly linked to gene-environment relationships when considering its invasion history in South America: (1) the adhesion potential of the Golden Mussel byssus that allows hitchhiking on the hulls of boats and other surfaces, (2) an unusual (for invertebrates) immunity to disease even under extremely high population densities, and (3) high tolerance/resistance to chemical control in industrial plants and facilities.

We identified two byssus proteins, Mepf1 and Mepf2 that are part of the plaque that confers high adhesion capability to solid surfaces (Table S4). The byssal plaque has strong cross-links between neighboring proteins, with a high content of DOPA, a modified amino acid, and metal atoms, ensuring consistent adhesion even in the presence of water [52]. These properties make byssus filaments about 6 times more resistant to tension than human tendons [36]. Further studies of such sequences may lead to the development of new methods to control *L. fortunei* by preventing or weakening byssus adhesion to surfaces.

We characterized at least eight genes involved in the signaling pathway of Toll-like receptors (Table S2). Recent studies of the urchin *Strongylocentrous purpuratus* [30], the leech *Hirudi medicinalis* [31] and cnidarians [32] showed that invertebrates may possess precursors of the adaptative immune system of mammals. Philipp *et al* [33] (2012) found that the mussel *M. edulis* has actually 27 Toll-like receptors instead of only two, as previously described [34], [35]. It is unlikely that the diversity and cosmopolitism of bivalves was possible without an excellent defense system. While viral and fungal infectious diseases represent the most serious threat to shrimp farming worldwide, this is not the case for bivalve cultivars. Studies have shown that the oyster *C. gigas* is easily farmed in Southern Brazilian waters highly contaminated with viruses (human adenovirus, noroviruses, Hepatitis A, JC Polyomavirus) and fecal coliforms [53].

The high resistance of *L. fortunei* to chemical control in industrial plants, is to some extent also observed in natural environments, where the Golden Mussel can survive inside the digestive tract of fishes [54]. Although it is more tolerant to chemical control in lab conditions than *D. polymorpha* [10], *L. fortunei* seems to be sensitive to the natural phenomenon of ‘dequada’ in the Pantanal wetlands. As the water quality deteriorates after extensive areas of vegetation are flooded during the rainy season, oxygen levels drop to zero and thus control *L. fortunei* populations [28]. In contrast to indigenous species from the Pantanal, *L. fortunei* lacks special adaptations to resist the low O_2_ conditions during the ‘dequada’. The Pacu fish (*Piaractus mesopotamicus*) for example, has a peroxide-depredate enzyme (GPx) with enhanced basal activity that helps it overcome oxidative stress [29]. *C. gigas* genome by Zhang *et al* (2012) showed no expansion in gene families related to the antioxidant system. This work has described a number of defense-related genes for *L. fortunei* (Table 4) that will allow future investigation of its antioxidant defenses genetic profile. If low resistence to ‘dequada’ is confirmed to be related to a less prominent antioxidant system, it should be a target for developing a control tool against *L. fortunei* infestation.

### Conclusions

About 80,000 unigenes were assembled and described and more than 20,000 of them functionally annotated. These are now available to study *L. fortunei’s* molecular metabolism and acclimation robustness. *Limnoperna fortunei* seems to have an expanded group of the hsp70 family which, together with genotypes favoring surface attachment and disease resistance, may be related to its ability to establish in a plethora of different environments. However, the possible lack of specific adaptations to oxidative stress may restrict its occurrence in wetlands and may be a target for the development of control strategies in both industrial facilities and natural environments.

## Methods

### 4.1. Ethics Statement

*L. fortunei* specimens were collected at the dam of Itaipu Hydroelectric Power Plant (25° 33′S, 54° 37′W) in Paraná, PR, Brazil. The bivalve is an exotic species and, therefore, do not characterize as endangered or protected species. The collection of mussels was authorized by Hélio Martins Fontes Júnior, Head of the Reservoir Department at the Itaipu Hydroelectric Power Plant.

### 4.2. Sampling, cDNA synthesis, normalization and 454 sequencing

Total RNA was extracted using Tri-Reagent (Sigma-Aldrich, USA) from five main tissues: mantle, gills, digestive gland and abductor muscle. RNA from all individuals (n = 10) and all five tissues were polled in equal quantities (800 ng) and enriched for mRNA using GenElute mRNA miniprep Kit (Sigma-Aldrich, St. Louis, Missouri, USA). The final enriched high-quality mRNA was used for cDNA synthesis.

cDNA was synthetized using MINT Kit (Evrogen, Moscow, Russia), following the manufacturer’s instructions, with a modified oligo-dT (5′-AAGCAGTGGTATCAACGCAGAGTCGCAGTCGGTACTTTTTTCTTTTTTV-3′) which has a poly-T stretch broken by the inclusion of an internal C to minimize the potential for 454 sequencing problems in this homopolymer stretch [40].

The cDNA library was normalized using the Trimmer Direct kit (Evrogen, Moscow, Russia) to prevent over-representation of the most common transcripts. Finally, approximately 10 µg of cDNA were used to construct two cDNA GS Junior 454 libraries that were sequenced separately. Roche GS Junior 454 pyrosequencing runs were conducted at the Biology Institute at the Federal University of Rio de Janeiro, Brazil.

### 4.3. Reads extraction and processing

Reads were extracted from the SFF files using Blanca and Chevreux’ssff_extract command line application (http://bioinf.comav.upv.es/sff_extract) and stored in FASTA files. Then, the original FASTA format sequencing reads were scanned and the adaptors used in the cDNA and 454 libraries preparation were masked using Cross_Match Script from the Phred, Phrap and Consed package (http://goo.gl/WGrNHF). To remove the masked adaptors from the clean reads a perl script was created and used (*biggerSubstringClip.pl*).

### 4.4. Assembly of reads and algorithms tested

FASTA reads free of adaptors were clustered using three strategies: CAP3 [21]; MIRA [20]; and Newbler [41]. CAP3 was run in a stringent clustering mode with flags set to -o 40 and -s 800, as described elsewhere [42], while MIRA was used as described in the manual for EST clustering of 454 sequences, using the flags -job = denovo, est, normal, and 454 [20]. Newbler was used with default settings and using the flag -cdna suggested for better results for cDNA single end reads from 454 libraries. CAP3 unigenes can be downloaded at http://goo.gl/oeXQHf.

### 4.5. Assembly evaluation

Three metrics were applied to identify the best clustered dataset. The internal and external consistency methods were already employed and detailed elsewhere [42]. The internal consistence evaluates the correct read to consensus mapping. It is tested when the original reads are aligned using BLAST against their consensus sequences. An ICI value of 100 means that the entire read was mapped with 100% sequence identity; lesser ICI values indicate a lower quality of consensus mapping.

The external consistency evaluates whether two different contigs should be regarded as one. A contig aligning perfectly against itself would have an ECI score of 100, whereas smaller ECI values would describe an average overlap size and identity value of sequence similarity between contigs. ECI scores higher than 75 [42], [43], [44] indicate that the consensus sequences are too close to each other; i.e., the program has separated sequences that represent the same gene and thus should be kept in the same contig.

In a final step, the number of unigenes annotated against a well-curated database (UNIPROT) was used as a metric of evaluation. Although different species have a specific amount of genes with the same function but different nucleotide sequences, the capability to assemble and annotate unigenes can be indicative of the effectiveness of the assemble algorithm.

### 4.6. SSR discovery

SciRoko program V3.3 [45] was used to identify microsatellite motifs. All types of SSR motifs, from dinucleotides to hexanucleotides were categorized using default settings.

### 4.7. Similarity searches against primary and secondary databases

The dataset of assembled sequences (unigenes of the best assembly after evaluating the three metrics) were compared against the NCBI non-redundant (nr) protein database [46], UNIPROT database [47], eggNOG [48] and KEGG [49] using BLAST with an e-value cutoff of 10e-05. Gene names were assigned to each assembled sequence based on the best BLAST hit (highest score). KEGG pathways were assigned to the assembled sequences using the online KEGG Automatic Annotation Server (KAAS), http://www.genome.jp/kegg/kass/. The bi-directional best hit (BBH) method was used to obtain KEGG Orthology (KO) assignment.

### 4.8. Transcriptome coverage, full-lengths

The quantity of full-length genes described for *L. fortunei* was estimated based on BLASTx searches between *L. fortunei* unigenes and *C. gigas* predicted proteins described by Zhang *et al.* [12] (available for download at http://oysterdb.cn/) and the percentage of coverage of *C. gigas* sequences with *L. fortunei* unigenes. A python script (Coverage.py) was developed to count how many *C. gigas* predicted proteins were covered 90% or more by unigenes from *L. fortunei* using the blast results as input. Such sequences were considered full-length described in this transcriptome for *L. fortunei.*

### 4.9. Searches for HSP70 and phylogenetic analysis

A total of 127 *L. fortunei* unigenes for HSP70 were found via BLASTx searches against *C. gigas* HSP70 predicted proteins. All 127 unigenes were translated to the best frame as protein sequences. We downloaded all 179 HSP70 protein sequences, representing 46 mollusks, from UNIPROT. Then, we conducted a phylogenetic analysis of all HSP70 sequences for mollusks. Protein sequences were aligned by CLUSTALW. The phylogenetic tree was constructed using maximum likelihood (ML) performed with the Whelan and Goldman model [50]. Robustness was accessed using bootstrapping (100 pseudo-replicates, values less than 30% are not shown). Evolutionary analysis were conducted in MEGA5 [51].

## Supporting Information

Table S1 **IDs of annotated unigenes containing SSRs.**
(DOCX)

Table S2 **IDs of unigenes annotated by KEGG and their respective KO identifiers.**
(XLS)

Table S3 **BLAST result for full-length search:** ***C. gigas*** **IDs together with the unigenes of** ***L. fortunei*** **annotated for each** ***C. gigas*** **sequence.**
(DOCX)

Table S4 **IDs of unigenes annotated as the footproteins Mepf1 and Mepf2.**
(XLSX)

Table S5 **IDs of unigenes annotated against** ***C. gigas*** **sequences related to cellular defenses.**
(DOCX)

Table S6 **IDs of unigenes filtered out by NCBI.**
(DOCX)
