# Supplementary material for: Gene Discovery through Transcriptome Sequencing for the Invasive Mussel Limnoperna fortunei
Source: PLoS One. 2014 Jul 21;9(7):e102973. doi: 10.1371/journal.pone.0102973 (PMC4105566; doi:10.1371/journal.pone.0102973)
Supplement: Table S1 — IDs of annotated unigenes containing SSRs. (DOCX) [file pone.0102973.s001.docx]

**SUPPORTING INFORMATION S1**

Uliano-Silva *et al.,* 2014. Gene discovery through transcriptome sequencing for the invasive mussel *Limnoperna fortunei.*

IDs of the unigenes having SSRs identified by SciRocko program and that were annotated through BLASTx searches against NCBI nr. The unigenes of *L. fortunei* are available for download at: **http://goo.gl/mNYPbX** .

Unigenes IDS

| Contig21 | HLFT7C201AZCHM | HLFT7C201BQGYK | HTNRPVY01AZIIT | |
| --- | --- | --- | --- | --- |
| Contig39 | HLFT7C201BJU4G | HLFT7C201BJL4Y | HTNRPVY01ANDI8 | |
| Contig89 | HLFT7C201BPD17 | HLFT7C201BFJSG | HTNRPVY01AUOU2 | |
| Contig115 | HLFT7C201BQWQ8 | HLFT7C201BYMHY | HTNRPVY01AV2GR | |
| Contig125 | HLFT7C201APUJJ | HLFT7C201ADDGY | HTNRPVY01ADMTT | |
| Contig203 | HLFT7C201A8GGC | HLFT7C201AKDSP | HTNRPVY01A4YE9 | |
| Contig266 | HLFT7C201ARBM3 | HLFT7C201BGL16 | HTNRPVY01B08E2 | |
| Contig281 | HLFT7C201AD9G5 | HLFT7C201A100X | HTNRPVY01APDX3 | |
| Contig344 | HLFT7C201A5TKR | HLFT7C201AF0VA | HTNRPVY01B2DVG | |
| Contig353 | HLFT7C201AEU6W | HLFT7C201A27G4 | HTNRPVY01AQ8VQ | |
| Contig377 | HLFT7C201AOYFO | HLFT7C201BCHN4 | HTNRPVY01AIY62 | |
| Contig379 | HLFT7C201AL40R | HLFT7C201BGSSD | HTNRPVY01A8MCR | |
| Contig380 | HLFT7C201AMXH1 | HLFT7C201BKN2D | HTNRPVY01B1CBH | |
| Contig400 | HLFT7C201BQMTL | HLFT7C201BEFTT | HTNRPVY01A1YMU | |
| Contig433 | HLFT7C201AYCH6 | HLFT7C201BMNYN | HTNRPVY01BMLTW | |
| Contig478 | HLFT7C201BT4VO | HLFT7C201BQ8FI | HTNRPVY01BLVEM | |
| Contig559 | HLFT7C201AXM91 | HLFT7C201ADTJ0 | HTNRPVY01A8CJ8 | |
| Contig564 | HLFT7C201AC0SK | HLFT7C201A6BVA | HTNRPVY01ALZFK | |
| Contig592 | HLFT7C201AT762 | HLFT7C201BECNP | HTNRPVY01BLVDA | |
| Contig616 | HLFT7C201AM397 | HLFT7C201AJIPB | HTNRPVY01BFD34 | |
| Contig624 | HLFT7C201AEFCL | HLFT7C201A931R | HTNRPVY01A9ONX | |
| Contig631 | HLFT7C201A47LM | HLFT7C201BVYZI | HTNRPVY01BE3ZJ | |
| Contig648 | HLFT7C201ARUS5 | HLFT7C201A7DYO | HTNRPVY01ANWUT | |
| Contig668 | HLFT7C201A53RF | HLFT7C201A77GY | HTNRPVY01BJLU5 | |
| Contig675 | HLFT7C201A8Y8Q | HLFT7C201A7HFE | HTNRPVY01BOFDJ | |
| Contig684 | HLFT7C201AOITY | HLFT7C201BSM0I | HTNRPVY01BAKSZ | |
| Contig685 | HLFT7C201B2HFH | HLFT7C201BN6RL | HTNRPVY01ARIL9 | |
| Contig725 | HLFT7C201BZULY | HLFT7C201A0ZBY | HTNRPVY01BRUQ9 | |
| Contig731 | HLFT7C201A2UQO | HLFT7C201BD467 | HTNRPVY01BU24J | |
| Contig793 | HLFT7C201BIW7O | HLFT7C201A26OB | HTNRPVY01ADG5B | |
| Contig800 | HLFT7C201BNTHV | HLFT7C201BVMT2 | HTNRPVY01AOCYL | |
| Contig806 | HLFT7C201BDP7N | HLFT7C201BXM6E | HTNRPVY01B1UNO | |
| Contig825 | HLFT7C201BJ76J | HLFT7C201BA3T0 | HTNRPVY01A2NTT | |
| Contig827 | HLFT7C201BB5SP | HLFT7C201AY2BA | HTNRPVY01BQSVB | |
| Contig830 | HLFT7C201BO1QZ | HLFT7C201APD54 | HTNRPVY01BXQI3 | |
| Contig831 | HLFT7C201AXAF4 | HLFT7C201AYL7F | HTNRPVY01A3FWO | |
| Contig834 | HLFT7C201BLCQT | HLFT7C201B0AZO | HTNRPVY01APAQ4 | |
| Contig841 | HLFT7C201AYO8R | HLFT7C201AFD8C | HTNRPVY01A8SYZ | |
| Contig843 | HLFT7C201BDF9Q | HLFT7C201BWKYY | HTNRPVY01A1PGG | |
| Contig923 | HLFT7C201BEH76 | HLFT7C201A9BD3 | HTNRPVY01BIPXY | |
| Contig927 | HLFT7C201A14RU | HLFT7C201AV57A | HTNRPVY01A8I5M | |
| Contig928 | HLFT7C201A9H4A | HLFT7C201BGJWG | HTNRPVY01BMHUA | |
| Contig949 | HLFT7C201BSDXD | HLFT7C201ASENA | HTNRPVY01ANMYG | |
| Contig958 | HLFT7C201AW033 | HLFT7C201AM0LL | HTNRPVY01ATFHV | |
| Contig960 | HLFT7C201BBYSL | HLFT7C201BN5VA | HTNRPVY01AV513 | |
| Contig966 | HLFT7C201BAGNE | HLFT7C201AEPDJ | HTNRPVY01BZA2W | |
| Contig967 | HLFT7C201BL2HB | HLFT7C201A5PX5 | HTNRPVY01BIJQE | |
| Contig975 | HLFT7C201BMX31 | HLFT7C201BHLW0 | HTNRPVY01AIG1B | |
| Contig986 | HLFT7C201AVHH4 | HLFT7C201ASGMH | HTNRPVY01AK36Y | |
| Contig988 | HLFT7C201ARUMF | HLFT7C201A39EH | HTNRPVY01AL5ZG | |
| Contig997 | HLFT7C201AK9PN | HLFT7C201AM685 | HTNRPVY01BE10X | |
| Contig1002 | HLFT7C201BPZ3H | HLFT7C201AUNY4 | HTNRPVY01ABM38 | |
| Contig1004 | HLFT7C201B2QWV | HLFT7C201AFDQ4 | HTNRPVY01BU7FM | |
| Contig1059 | HLFT7C201BKZ9U | HLFT7C201AGC2V | HTNRPVY01BSJIS | |
| Contig1085 | HLFT7C201BLYH5 | HLFT7C201BGGFK | HTNRPVY01BJBWV | |
| Contig1087 | HLFT7C201BFDXQ | HLFT7C201BWXSL | HTNRPVY01BHH2L | |
| Contig1092 | HLFT7C201BOSCD | HLFT7C201A6PCR | HTNRPVY01BEIB0 | |
| Contig1099 | HLFT7C201BADCT | HLFT7C201AXQ82 | HTNRPVY01AVZYM | |
| Contig1105 | HLFT7C201A46TK | HLFT7C201BMN7Q | HTNRPVY01AFA71 | |
| Contig1122 | HLFT7C201AQZVF | HLFT7C201AY75Q | HTNRPVY01AZW8K | |
| Contig1128 | HLFT7C201BKNFO | HLFT7C201BUOUM | HTNRPVY01ANDLW | |
| Contig1129 | HLFT7C201AZ01T | HLFT7C201AH955 | HTNRPVY01BQMUT | |
| Contig1137 | HLFT7C201BZKMD | HLFT7C201B0HCL | HTNRPVY01BPQIO | |
| Contig1138 | HLFT7C201BIGXF | HLFT7C201AWFFH | HTNRPVY01ANJ2R | |
| Contig1139 | HLFT7C201BCNRE | HLFT7C201B2N3G | HTNRPVY01A3SOR | |
| Contig1151 | HLFT7C201BTPAL | HLFT7C201AU1DD | HTNRPVY01AVZ8L | |
| Contig1153 | HLFT7C201B2Q5H | HLFT7C201A551Z | HTNRPVY01BZA6V | |
| Contig1158 | HLFT7C201AVAX2 | HLFT7C201A7H9I | HTNRPVY01BWRRH | |
| Contig1159 | HLFT7C201BLIUM | HLFT7C201ABLVA | HTNRPVY01AJPCF | |
| Contig1170 | HLFT7C201AYDHJ | HLFT7C201AD3DW | HTNRPVY01BAGTM | |
| Contig1171 | HLFT7C201AHODW | HLFT7C201BFJ0M | HTNRPVY01A9IJ8 | |
| Contig1173 | HLFT7C201AYINE | HLFT7C201BH3IL | HTNRPVY01BFNA6 | |
| Contig1174 | HLFT7C201ADSWP | HLFT7C201APIEU | HTNRPVY01B2JSI | |
| Contig1175 | HLFT7C201AKKMJ | HLFT7C201BDTQJ | HTNRPVY01BB442 | |
| Contig1185 | HLFT7C201BX3JJ | HLFT7C201B14WI | HTNRPVY01BRU52 | |
| Contig1190 | HLFT7C201B3V8X | HLFT7C201BQS09 | HTNRPVY01BFAO4 | |
| Contig1194 | HLFT7C201BL164 | HLFT7C201BI2Z1 | HTNRPVY01BW7I0 | |
| Contig1204 | HLFT7C201A88FB | HLFT7C201BV78U | HTNRPVY01AV2V3 | |
| Contig1218 | HLFT7C201BH021 | HLFT7C201BQF80 | HTNRPVY01AXZM1 | |
| Contig1221 | HLFT7C201AIG9P | HLFT7C201AFJ9L | HTNRPVY01A0F95 | |
| Contig1222 | HLFT7C201B26P6 | HLFT7C201ARYUO | HTNRPVY01BT4HT | |
| Contig1233 | HLFT7C201BJCFP | HLFT7C201B1VM9 | HTNRPVY01B2XAC | |
| Contig1241 | HLFT7C201ALJCP | HLFT7C201AFQY7 | HTNRPVY01A5S5M | |
| Contig1257 | HLFT7C201AKJ8S | HLFT7C201BXHQJ | HTNRPVY01A1C5W | |
| Contig1289 | HLFT7C201AYSVG | HLFT7C201BAJPM | HTNRPVY01BNWYY | |
| Contig1293 | HLFT7C201BVW1G | HLFT7C201BFDXX | HTNRPVY01BTB2Q | |
| Contig1333 | HLFT7C201AO43M | HLFT7C201A9KZ4 | HTNRPVY01AKW4Z | |
| Contig1370 | HLFT7C201BUIHX | HLFT7C201BRPBA | HTNRPVY01ALPY7 | |
| Contig1381 | HLFT7C201BZHXI | HLFT7C201BWEMN | HTNRPVY01AE5HZ | |
| Contig1396 | HLFT7C201BHBC6 | HLFT7C201B3S5M | HTNRPVY01AGFK9 | |
| Contig1405 | HLFT7C201A0GX6 | HLFT7C201AB5Z7 | HTNRPVY01BSJXG | |
| Contig1409 | HLFT7C201ACUHU | HLFT7C201B291T | HTNRPVY01BLWBG | |
| Contig1414 | HLFT7C201ALTP9 | HLFT7C201BVNI6 | HTNRPVY01A4OSF | |
| Contig1459 | HLFT7C201A4RMG | HLFT7C201BGYLE | HTNRPVY01A9OI5 | |
| Contig1463 | HLFT7C201BJLR4 | HLFT7C201A27EG | HTNRPVY01AXN3E | |
| Contig1469 | HLFT7C201B3SA8 | HLFT7C201AB51I | HTNRPVY01BKRAB | |
| Contig1479 | HLFT7C201AZ6NE | HLFT7C201A3L5S | HTNRPVY01A38LW | |
| Contig1504 | HLFT7C201AYYJC | HLFT7C201BI0IT | HTNRPVY01AJVSE | |
| Contig1505 | HLFT7C201A4UD5 | HLFT7C201BFNY2 | HTNRPVY01BADII | |
| Contig1552 | HLFT7C201A3MMC | HLFT7C201BOMIP | HTNRPVY01B1CMW | |
| Contig1572 | HLFT7C201BFDPN | HLFT7C201AOCEY | HTNRPVY01BKEE7 | |
| Contig1579 | HLFT7C201ARBGC | HLFT7C201AZ6MU | HTNRPVY01BA9JR | |
| Contig1598 | HLFT7C201BU661 | HLFT7C201A1O4G | HTNRPVY01BJBMD | |
| Contig1604 | HLFT7C201BMOJV | HLFT7C201B1IIQ | HTNRPVY01ARFFS | |
| Contig1608 | HLFT7C201ASTLL | HLFT7C201BDAD3 | HTNRPVY01BF0JI | |
| Contig1611 | HLFT7C201A14P4 | HLFT7C201ADCXQ | HTNRPVY01ATL0I | |
| Contig1632 | HLFT7C201BLWJP | HLFT7C201AFG5C | HTNRPVY01BFWQ7 | |
| Contig1642 | HLFT7C201A3SY9 | HLFT7C201BH4OY | HTNRPVY01ATC2K | |
| Contig1662 | HLFT7C201ABV2D | HLFT7C201AG2XL | HTNRPVY01BOOL6 | |
| Contig1669 | HLFT7C201AQS0Y | HLFT7C201BVTXF | HTNRPVY01BMT9F | |
| Contig1687 | HLFT7C201ALVQO | HLFT7C201BEL3H | HTNRPVY01BKQWB | |
| Contig1697 | HLFT7C201AW18N | HLFT7C201BJCCD | HTNRPVY01AFD82 | |
| Contig1715 | HLFT7C201A2Z91 | HLFT7C201A5XJB | HTNRPVY01BF0OS | |
| Contig1721 | HLFT7C201BUW85 | HLFT7C201A8DWF | HTNRPVY01AYSL5 | |
| Contig1738 | HLFT7C201ATIWJ | HLFT7C201BZKTO | HTNRPVY01AXQK1 | |
| Contig1740 | HLFT7C201ATPG9 | HLFT7C201BJMFU | HTNRPVY01AN9MI | |
| Contig1743 | HLFT7C201ACHO9 | HLFT7C201AR75Z | HTNRPVY01AC31Z | |
| Contig1787 | HLFT7C201BLIVO | HLFT7C201BESJE | HTNRPVY01A68DI | |
| Contig1801 | HLFT7C201BSZNO | HLFT7C201AT5F5 | HTNRPVY01AT84A | |
| Contig1808 | HLFT7C201A9FBC | HLFT7C201BNTHS | HTNRPVY01ARR7C | |
| Contig1811 | HLFT7C201AV5UT | HLFT7C201ANT5N | HTNRPVY01ANQ5Z | |
| Contig1822 | HLFT7C201A5C47 | HTNRPVY01AN5UI | HTNRPVY01BKHY0 | |
| Contig1826 | HLFT7C201BI2JP | HTNRPVY01AU609 | HTNRPVY01A1LA8 | |
| Contig1839 | HLFT7C201A4HTW | HTNRPVY01AB8E7 | HTNRPVY01ARX8P | |
| Contig1841 | HLFT7C201BOPCR | HTNRPVY01AEXV3 | HTNRPVY01ACOIZ | |
| Contig1844 | HLFT7C201A7HMU | HTNRPVY01A3PQJ | HTNRPVY01AZ7F8 | |
| Contig1851 | HLFT7C201BFQQ9 | HTNRPVY01AEH8J | HTNRPVY01BEI87 | |
| Contig1859 | HLFT7C201BJFRO | HTNRPVY01A5610 | HTNRPVY01A3P5R | |
| Contig1872 | HLFT7C201A0AAG | HTNRPVY01BNJDL | HTNRPVY01BCBGT | |
| Contig1894 | HLFT7C201BXT2I | HTNRPVY01A6RT2 | HTNRPVY01BTE44 | |
| Contig1901 | HLFT7C201BYLUG | HTNRPVY01A2ZWY | HTNRPVY01BADXD | |
| Contig1929 | HLFT7C201ATPJ3 | HTNRPVY01AGSCJ | HTNRPVY01B1U0F | |
| Contig1934 | HLFT7C201BPQG6 | HTNRPVY01BXNCB | HTNRPVY01B2QTP | |
| Contig1950 | HLFT7C201AJFR3 | HTNRPVY01BRE1T | HTNRPVY01A8V97 | |
| Contig1963 | HLFT7C201BNDLQ | HTNRPVY01B0MMW | HTNRPVY01BCERQ | |
| Contig1964 | HLFT7C201BEHS1 | HTNRPVY01AJ40B | HTNRPVY01BI2TK | |
| Contig1983 | HLFT7C201AZYEC | HTNRPVY01AOL9U | HTNRPVY01A2T64 | |
| Contig1987 | HLFT7C201AN8U7 | HTNRPVY01B1UPY | HTNRPVY01BAP1U | |
| Contig2004 | HLFT7C201AKNXD | HTNRPVY01BT77Y | HTNRPVY01BC6H4 | |
| Contig2046 | HLFT7C201BJR5Y | HTNRPVY01AFQDX | HTNRPVY01BQZEB | |
| Contig2052 | HLFT7C201AYYS0 | HTNRPVY01BLSKQ | HTNRPVY01AITHT | |
| Contig2053 | HLFT7C201AKRBD | HTNRPVY01A7RP4 | HTNRPVY01A0AFV | |
| Contig2073 | HLFT7C201AQ8I1 | HTNRPVY01AI5V6 | HTNRPVY01AQ2Q7 | |
| Contig2092 | HLFT7C201BHD7A | HTNRPVY01APRJD | HTNRPVY01BDY71 | |
| Contig2111 | HLFT7C201AS99D | HTNRPVY01AB8J1 | HTNRPVY01A26MV | |
| Contig2112 | HLFT7C201BVCRV | HTNRPVY01BHLIL | HTNRPVY01BKQI6 | |
| Contig2116 | HLFT7C201AS3W5 | HTNRPVY01AM7WP | HTNRPVY01AC7EX | |
| Contig2133 | HLFT7C201AMIJO | HTNRPVY01AYO36 | HTNRPVY01AADSH | |
| Contig2136 | HLFT7C201A61PO | HTNRPVY01AERN7 | HTNRPVY01AFGWA | |
| Contig2146 | HLFT7C201A7A9K | HTNRPVY01AG150 | HTNRPVY01AM6XZ | |
| Contig2151 | HLFT7C201BKKUS | HTNRPVY01A9FKT | HTNRPVY01A0DUV | |
| Contig2154 | HLFT7C201B0PZU | HTNRPVY01A2T9H | HTNRPVY01BG4ZS | |
| Contig2155 | HLFT7C201BK0EF | HTNRPVY01A0WBV | HTNRPVY01A3Y76 | |
| Contig2164 | HLFT7C201BEB89 | HTNRPVY01A05V5 | HTNRPVY01A70EO | |
| Contig2180 | HLFT7C201AXGOX | HTNRPVY01A2A44 | HTNRPVY01A6OVZ | |
| Contig2201 | HLFT7C201A2A6X | HTNRPVY01B0494 | HTNRPVY01BS2HA | |
| Contig2202 | HLFT7C201BT5NN | HTNRPVY01A0MW1 | HTNRPVY01AJXYN | |
| Contig2209 | HLFT7C201AQM2V | HTNRPVY01AJPF7 | HTNRPVY01B3L2F | |
| Contig2224 | HLFT7C201BAKRG | HTNRPVY01AHA6V | HTNRPVY01BG439 | |
| Contig2241 | HLFT7C201AIZH3 | HTNRPVY01AC4PD | HTNRPVY01A56JN | |
| Contig2246 | HLFT7C201BSPZX | HTNRPVY01AO094 | HTNRPVY01BLSZE | |
| Contig2278 | HLFT7C201AG8IU | HTNRPVY01A9IZT | HTNRPVY01BRUFQ | |
| Contig2282 | HLFT7C201AIGI0 | HTNRPVY01AR1SH | HTNRPVY01AMK1R | |
| Contig2284 | HLFT7C201AK0RG | HTNRPVY01A7KAN | HTNRPVY01BC3HX | |
| Contig2286 | HLFT7C201BF3I6 | HTNRPVY01BKOLF | HTNRPVY01ACBDD | |
| Contig2298 | HLFT7C201AINLD | HTNRPVY01AP3TD | HTNRPVY01AOMEW | |
| Contig2300 | HLFT7C201BTR38 | HTNRPVY01BLFSZ | HTNRPVY01BP3EF | |
| Contig2305 | HLFT7C201AJU01 | HTNRPVY01BHX8L | HTNRPVY01AEV0H | |
| Contig2310 | HLFT7C201B02E8 | HTNRPVY01BHEUS | HTNRPVY01BS9G4 | |
| Contig2378 | HLFT7C201AV8FG | HTNRPVY01AU66R | HTNRPVY01BC6VN | |
| Contig2380 | HLFT7C201AB7ZH | HTNRPVY01BDZFG | HTNRPVY01BE0VJ | |
| Contig2381 | HLFT7C201BS3E5 | HTNRPVY01AT1TE | HTNRPVY01BSMQG | |
| Contig2382 | HLFT7C201BA9LN | HTNRPVY01BVMII | HTNRPVY01BF6DT | |
| Contig2423 | HLFT7C201BDSI3 | HTNRPVY01BIDAF | HTNRPVY01AFBG6 | |
| Contig2426 | HLFT7C201BRBHK | HTNRPVY01BJ4MT | HTNRPVY01ABAB9 | |
| Contig2463 | HLFT7C201AYFUU | HTNRPVY01BV8NK | HTNRPVY01A2G3F | |
| Contig2473 | HLFT7C201AOSBC | HTNRPVY01BK6WC | HTNRPVY01B3B76 | |
| Contig2494 | HLFT7C201BG1PU | HTNRPVY01BZ3KJ | HTNRPVY01AAERX | |
| Contig2539 | HLFT7C201ABSRX | HTNRPVY01BTI95 | HTNRPVY01BOIAO | |
| Contig2551 | HLFT7C201ALWC8 | HTNRPVY01BNV7N | HTNRPVY01A55VV | |
| Contig2553 | HLFT7C201A0DCA | HTNRPVY01A23KH | HTNRPVY01ASKMU | |
| Contig2558 | HLFT7C201AME76 | HTNRPVY01BE1XQ | HTNRPVY01BJVC5 | |
| Contig2586 | HLFT7C201AM1MI | HTNRPVY01ASKO4 | HTNRPVY01A9L87 | |
| Contig2609 | HLFT7C201A6X3I | HTNRPVY01ABVHH | HTNRPVY01B3C2O | |
| Contig2632 | HLFT7C201BEB9V | HTNRPVY01AGF2K | HTNRPVY01BUWVD | |
| Contig2666 | HLFT7C201BL4R4 | HTNRPVY01AU910 | HTNRPVY01AU9PH | |
| Contig2692 | HLFT7C201BXZ3I | HTNRPVY01AJFKN | HTNRPVY01AW8LY | |
| Contig2702 | HLFT7C201BFMWE | HTNRPVY01A6LEO | HTNRPVY01BXAAI | |
| Contig2752 | HLFT7C201ADDOT | HTNRPVY01AQI8L | HTNRPVY01A14WK | |
| Contig2769 | HLFT7C201BAZJY | HTNRPVY01A58TA | HTNRPVY01AB2JT | |
| Contig2784 | HLFT7C201A7UAL | HTNRPVY01AUKUH | HTNRPVY01AIKA1 | |
| Contig2798 | HLFT7C201BG8OQ | HTNRPVY01ACKPU | HTNRPVY01BOOHV | |
| Contig2811 | HLFT7C201BIKFD | HTNRPVY01BU35Q | HTNRPVY01BVDNR | |
| Contig2826 | HLFT7C201A9UHS | HTNRPVY01BL7YT | HTNRPVY01A4N8T | |
| Contig2836 | HLFT7C201AYC2V | HTNRPVY01AFZP0 | HTNRPVY01AT12A | |
| Contig2867 | HLFT7C201BIPO5 | HTNRPVY01ATGHJ | HTNRPVY01B14PQ | |
| Contig2907 | HLFT7C201AFAZ4 | HTNRPVY01A3PD7 | HTNRPVY01BFXW0 | |
| Contig2955 | HLFT7C201A1IVO | HTNRPVY01AQ546 | HTNRPVY01AE8P0 | |
| Contig2957 | HLFT7C201B0TF3 | HTNRPVY01BOMDF | HTNRPVY01AS95P | |
| Contig2973 | HLFT7C201BESNQ | HTNRPVY01AS30M | HTNRPVY01BGYAB | |
| Contig3000 | HLFT7C201BXNHY | HTNRPVY01AZ02Y | HTNRPVY01AKKPY | |
| Contig3017 | HLFT7C201BIZ6N | HTNRPVY01BVM7P | HTNRPVY01BXQ0R | |
| Contig3027 | HLFT7C201AOOL8 | HTNRPVY01ACHUT | HTNRPVY01BIF53 | |
| Contig3058 | HLFT7C201B2NNE | HTNRPVY01AB2L8 | HTNRPVY01AQMP7 | |
| Contig3072 | HLFT7C201B2KUQ | HTNRPVY01AGLZ1 | HTNRPVY01A5NPX | |
| Contig3078 | HLFT7C201BHEHO | HTNRPVY01BJ49L | HTNRPVY01B02PM | |
| Contig3094 | HLFT7C201BUQZT | HTNRPVY01BR1NS | HTNRPVY01ARURT | |
| Contig3098 | HLFT7C201AMUY1 | HTNRPVY01AP6N3 | HTNRPVY01A3MY9 | |
| Contig3099 | HLFT7C201A4HSE | HTNRPVY01AVGXN | HTNRPVY01A0NSX | |
| Contig3114 | HLFT7C201AH65V | HTNRPVY01BLDQV | HTNRPVY01ASAP7 | |
| Contig3127 | HLFT7C201A2IDS | HTNRPVY01ANZJN | HTNRPVY01AMBZC | |
| Contig3131 | HLFT7C201A468K | HTNRPVY01BNTDJ | HTNRPVY01BH3X2 | |
| Contig3180 | HLFT7C201AN29P | HTNRPVY01AKEQ4 | HTNRPVY01BCERH | |
| Contig3186 | HLFT7C201AZTZO | HTNRPVY01BMW7Y | HTNRPVY01BGLNC | |
| Contig3194 | HLFT7C201A5JTK | HTNRPVY01BVVQX | HTNRPVY01BP4DI | |
| Contig3212 | HLFT7C201BF9RX | HTNRPVY01AVASL | HTNRPVY01BM1GN | |
| Contig3216 | HLFT7C201BAJP8 | HTNRPVY01BSHRN | HTNRPVY01BFJSS | |
| Contig3222 | HLFT7C201BCKP1 | HTNRPVY01BFZRB | HTNRPVY01AXZWE | |
| Contig3226 | HLFT7C201BTBXG | HTNRPVY01BAM6J | HTNRPVY01AFTH0 | |
| Contig3243 | HLFT7C201BMXT2 | HTNRPVY01BQYWG | HTNRPVY01AJO0K | |
| Contig3257 | HLFT7C201AE8CI | HTNRPVY01AKA4X | HTNRPVY01BERFV | |
| Contig3263 | HLFT7C201BX5YD | HTNRPVY01ASGSC | HTNRPVY01BJ5AQ | |
| Contig3296 | HLFT7C201ABO4M | HTNRPVY01A3ADL | HTNRPVY01AR7P2 | |
| Contig3297 | HLFT7C201AEPAG | HTNRPVY01BJOZ5 | HTNRPVY01AQ8NS | |
| Contig3300 | HLFT7C201BE0S2 | HTNRPVY01AR1HY | HTNRPVY01A8P0C | |
| Contig3351 | HLFT7C201AS6SQ | HTNRPVY01ANQM6 | HTNRPVY01BBUY7 | |
| Contig3363 | HLFT7C201ACBHC | HTNRPVY01BBX95 | HTNRPVY01AN3DY | |
| Contig3375 | HLFT7C201ABPOW | HTNRPVY01BE4VD | HTNRPVY01BBCI9 | |
| Contig3408 | HLFT7C201AVP1D | HTNRPVY01BS2JH | HTNRPVY01BPNQY | |
| Contig3448 | HLFT7C201BA214 | HTNRPVY01AWE07 | HTNRPVY01AKQNL | |
| Contig3466 | HLFT7C201BS8YX | HTNRPVY01BRFJG | HTNRPVY01A9OAD | |
| Contig3479 | HLFT7C201ACUTI | HTNRPVY01A5D1R | HTNRPVY01ASARK | |
| Contig3491 | HLFT7C201B3MR7 | HTNRPVY01BBFPU | HTNRPVY01AC9V2 | |
| Contig3513 | HLFT7C201AWB9U | HTNRPVY01BF3NT | HTNRPVY01AC068 | |
| Contig3517 | HLFT7C201BUKE5 | HTNRPVY01AE7K9 | HTNRPVY01BTRQN | |
| Contig3574 | HLFT7C201A3DD8 | HTNRPVY01BC92P | HTNRPVY01ARSIF | |
| Contig3587 | HLFT7C201BXNI8 | HTNRPVY01AQ2L4 | HTNRPVY01BDV8U | |
| Contig3592 | HLFT7C201BWUWZ | HTNRPVY01BM4T4 | HTNRPVY01A4QZ1 | |
| Contig3604 | HLFT7C201BI8ZC | HTNRPVY01BT703 | HTNRPVY01A1E06 | |
| Contig3615 | HLFT7C201ADZT7 | HTNRPVY01BV9LK | HTNRPVY01AA9WV | |
| Contig3631 | HLFT7C201BL9A4 | HTNRPVY01A4EON | HTNRPVY01BBPWQ | |
| Contig3650 | HLFT7C201ARIP1 | HTNRPVY01A5ZVS | HTNRPVY01ARBYB | |
| Contig3652 | HLFT7C201AGDFF | HTNRPVY01BH4CA | HTNRPVY01BAQ2W | |
| Contig3660 | HLFT7C201AWOTC | HTNRPVY01AHBBK | HTNRPVY01AVZXM | |
| Contig3662 | HLFT7C201BOLZP | HTNRPVY01BPZYR | HTNRPVY01BQ2VW | |
| Contig3664 | HLFT7C201A646M | HTNRPVY01B0TQL | HTNRPVY01AJRWA | |
| Contig3670 | HLFT7C201BAQJW | HTNRPVY01BC0H6 | HTNRPVY01AM4S5 | |
| Contig3679 | HLFT7C201BIM2N | HTNRPVY01AD5CZ | HTNRPVY01AGV6I | |
| Contig3703 | HLFT7C201ANJJF | HTNRPVY01AXXNG | HTNRPVY01A47OX | |
| Contig3712 | HLFT7C201BWBO2 | HTNRPVY01AG2LN | HTNRPVY01BX3TY | |
| Contig3727 | HLFT7C201AA33F | HTNRPVY01BJL0L | HTNRPVY01BDSSP | |
| Contig3732 | HLFT7C201BWBKN | HTNRPVY01BA6R4 | HTNRPVY01ADF4A | |
| Contig3739 | HLFT7C201BVDGW | HTNRPVY01A8GIN | HTNRPVY01AIEFU | |
| Contig3752 | HLFT7C201BWRG5 | HTNRPVY01BUT4O | HTNRPVY01AATD8 | |
| Contig3759 | HLFT7C201AKOL5 | HTNRPVY01AZ7OC | HTNRPVY01A674D | |
| Contig3770 | HLFT7C201BBCLO | HTNRPVY01BK3QQ | HTNRPVY01BLS5X | |
| Contig3771 | HLFT7C201AWH4H | HTNRPVY01A1XU6 | HTNRPVY01ABGKF | |
| Contig3817 | HLFT7C201A0AJF | HTNRPVY01BISW3 | HTNRPVY01AVZSH | |
| Contig3835 | HLFT7C201A1E8X | HTNRPVY01ANAIJ | HTNRPVY01AV9P4 | |
| Contig3837 | HLFT7C201AC7BY | HTNRPVY01AL44E | HTNRPVY01A8P1M | |
| Contig3846 | HLFT7C201BJING | HTNRPVY01BGCQH | HTNRPVY01BA6DO | |
| Contig3854 | HLFT7C201BP0DV | HTNRPVY01AVGGR | HTNRPVY01B0DLV | |
| Contig3862 | HLFT7C201BLMK2 | HTNRPVY01BSM2H | HTNRPVY01BRXK1 | |
| Contig3866 | HLFT7C201AWR0B | HTNRPVY01AJMCA | HTNRPVY01A2QV8 | |
| Contig3889 | HLFT7C201AQ5YT | HTNRPVY01B22SG | HTNRPVY01A2UG6 | |
| Contig3891 | HLFT7C201BNNH7 | HTNRPVY01ANMV3 | HTNRPVY01A230M | |
| Contig3901 | HLFT7C201A8PG3 | HTNRPVY01AXM2X | HTNRPVY01BLPPE | |
| Contig3909 | HLFT7C201AJCMD | HTNRPVY01BUBRH | HTNRPVY01BXAMF | |
| Contig3919 | HLFT7C201BLM9I | HTNRPVY01AKT0C | HTNRPVY01A6I4I | |
| Contig3948 | HLFT7C201AI9OW | HTNRPVY01AGMX4 | HTNRPVY01AIDSM | |
| Contig3958 | HLFT7C201ABYGY | HTNRPVY01BRCE0 | HTNRPVY01BT1KV | |
| Contig3960 | HLFT7C201BC381 | HTNRPVY01AD9WU | HTNRPVY01BJXZ2 | |
| Contig3964 | HLFT7C201BHCDT | HTNRPVY01B2HIY | HTNRPVY01AR39E | |
| Contig3980 | HLFT7C201BFWLI | HTNRPVY01BCEYH | HTNRPVY01A5TF6 | |
| Contig3983 | HLFT7C201BHK6F | HTNRPVY01BYS30 | HTNRPVY01BAADA | |
| Contig3985 | HLFT7C201B0F37 | HTNRPVY01AKAXJ | HTNRPVY01BPD7E | |
| Contig3986 | HLFT7C201BN5Y8 | HTNRPVY01ACELV | HTNRPVY01A5QQ2 | |
| Contig3998 | HLFT7C201ABSFR | HTNRPVY01BTPVI | HTNRPVY01AURMZ | |
| Contig4010 | HLFT7C201AS9WD | HTNRPVY01BNWV4 | HTNRPVY01BP3YA | |
| Contig4038 | HLFT7C201AGJL5 | HTNRPVY01BVZQJ | HTNRPVY01BLQG2 | |
| Contig4058 | HLFT7C201AKBG6 | HTNRPVY01AQJKO | HTNRPVY01AFW1G | |
| Contig4061 | HLFT7C201AKA52 | HTNRPVY01BDZM8 | HTNRPVY01A7W5N | |
| Contig4062 | HLFT7C201BK3AH | HTNRPVY01BO1DF | HTNRPVY01ACCES | |
| Contig4066 | HLFT7C201AYYGX | HTNRPVY01BABCG | HTNRPVY01BOB3L | |
| Contig4078 | HLFT7C201BYR4K | HTNRPVY01AUEZ6 | HTNRPVY01BSAYA | |
| Contig4157 | HLFT7C201AROA4 | HTNRPVY01AUD5N | HTNRPVY01A3CJR | |
| Contig4164 | HLFT7C201BEBQE | HTNRPVY01BXQ38 | HTNRPVY01BDMC1 | |
| Contig4176 | HLFT7C201BYYX8 | HTNRPVY01B0VWE | HTNRPVY01BRNW6 | |
| Contig4181 | HLFT7C201BSNAA | HTNRPVY01ANWCH | HTNRPVY01BHUMB | |
| Contig4217 | HLFT7C201A90NN | HTNRPVY01BUXJI | HTNRPVY01AF66Y | |
| Contig4235 | HLFT7C201BLYR0 | HTNRPVY01BXXDM | HTNRPVY01AAP4S | |
| Contig4243 | HLFT7C201AGPAZ | HTNRPVY01BKGYL | HTNRPVY01BS2YV | |
| Contig4263 | HLFT7C201B1FFQ | HTNRPVY01B1VCP | HTNRPVY01AAKLX | |
| Contig4265 | HLFT7C201AB49E | HTNRPVY01B05SR | HTNRPVY01AGWQO | |
| Contig4271 | HLFT7C201AWE3S | HTNRPVY01AQ8Q6 | HTNRPVY01AEL8R | |
| Contig4277 | HLFT7C201AQWJG | HTNRPVY01BW4X8 | HTNRPVY01AK3LD | |
| Contig4280 | HLFT7C201BYRZW | HTNRPVY01A2EC5 | HTNRPVY01BVL8Q | |
| Contig4281 | HLFT7C201A0V86 | HTNRPVY01ADZDC | HTNRPVY01A5ARP | |
| Contig4283 | HLFT7C201BC0XL | HTNRPVY01ALL6Y | HTNRPVY01AKRQS | |
| Contig4284 | HLFT7C201BWFEU | HTNRPVY01A9N7E | HTNRPVY01BV5MJ | |
| Contig4298 | HLFT7C201AXTH4 | HTNRPVY01A6L7W | HTNRPVY01BB7TT | |
| Contig4303 | HLFT7C201A7KPL | HTNRPVY01AB1JS | HTNRPVY01AH38C | |
| Contig4324 | HLFT7C201BYDG5 | HTNRPVY01B20H3 | HTNRPVY01BJ34Q | |
| Contig4350 | HLFT7C201A3SDZ | HTNRPVY01A0S0S | HTNRPVY01AP1HJ | |
| Contig4360 | HLFT7C201AJMSG | HTNRPVY01BPXKC | HTNRPVY01AX9C9 | |
| Contig4368 | HLFT7C201BCK65 | HTNRPVY01A8AIY | HTNRPVY01AOB27 | |
| Contig4373 | HLFT7C201BX212 | HTNRPVY01AQVZB | HTNRPVY01ASW6M | |
| Contig4392 | HLFT7C201AOR77 | HTNRPVY01AATZV | HTNRPVY01ADMJ7 | |
| Contig4398 | HLFT7C201BJ7K8 | HTNRPVY01A0WLR | HTNRPVY01BEOWW | |
| Contig4402 | HLFT7C201A3CQ1 | HTNRPVY01B2G0F | HTNRPVY01AKNG6 | |
| Contig4407 | HLFT7C201BQS4U | HTNRPVY01AY5SL | HTNRPVY01A03JL | |
| Contig4424 | HLFT7C201BTLW9 | HTNRPVY01BCTWQ | HTNRPVY01A9631 | |
| Contig4439 | HLFT7C201A967P | HTNRPVY01BWUCH | HTNRPVY01AY8DL | |
| Contig4457 | HLFT7C201A8ZRD | HTNRPVY01A0DIY | HTNRPVY01BKG4M | |
| Contig4459 | HLFT7C201A3QAT | HTNRPVY01A38NZ | HTNRPVY01BHBAS | |
| Contig4463 | HLFT7C201BDWQZ | HTNRPVY01AAKA8 | HTNRPVY01BOSIF | |
| Contig4482 | HLFT7C201AXEG3 | HTNRPVY01AWI22 | HTNRPVY01A6YPH | |
| Contig4484 | HLFT7C201A3I46 | HTNRPVY01AJOU6 | HTNRPVY01B3C6J | |
| Contig4485 | HLFT7C201ABSHS | HTNRPVY01BO07C | HTNRPVY01A8L6F | |
| Contig4490 | HLFT7C201AHKW1 | HTNRPVY01BMLVC | HTNRPVY01BOFJS | |
| Contig4500 | HLFT7C201AGVEP | HTNRPVY01AY1RN | HTNRPVY01AJPKS | |
| Contig4504 | HLFT7C201BTL59 | HTNRPVY01A9XW6 | HTNRPVY01BN9SV | |
| Contig4516 | HLFT7C201AMOON | HTNRPVY01BJY4F | HTNRPVY01A3GQR | |
| Contig4518 | HLFT7C201BKP82 | HTNRPVY01BF0CQ | HTNRPVY01BTUW0 | |
| Contig4519 | HLFT7C201A3JJP | HTNRPVY01BX0RU | HTNRPVY01AZ9ZP | |
| Contig4523 | HLFT7C201BJH8B | HTNRPVY01BL8N8 | HTNRPVY01AW7IJ | |
| Contig4532 | HLFT7C201AECOI | HTNRPVY01ANDZX | HTNRPVY01BBO6I | |
| Contig4540 | HLFT7C201AHUTH | HTNRPVY01AEEV3 | HTNRPVY01APH14 | |
| Contig4547 | HLFT7C201B3O1Z | HTNRPVY01ARI3X | HTNRPVY01AVSMT | |
| Contig4558 | HLFT7C201BWEMY | HTNRPVY01BRR3H | HTNRPVY01A0ZSB | |
| Contig4570 | HLFT7C201A4NZG | HTNRPVY01BNW9X | HTNRPVY01AAZ5X | |
| Contig4586 | HLFT7C201ALO7H | HTNRPVY01A8PR0 | HTNRPVY01AUUCB | |
| Contig4593 | HLFT7C201AO172 | HTNRPVY01BH7ES | HTNRPVY01BICXQ | |
| HLFT7C201AQSSV | HLFT7C201BMBJ3 | HTNRPVY01BL2P7 | HTNRPVY01AEH4A | |
| HLFT7C201BTYKF | HLFT7C201BVSYV | HTNRPVY01BMT4S | HTNRPVY01AXHW5 | |
| HLFT7C201BTYFS | HLFT7C201AJ4TG | HTNRPVY01BVAKT | HTNRPVY01BFTH6 | |
| HLFT7C201AV6N3 | HLFT7C201BHXWI | HTNRPVY01BLVXH | HTNRPVY01A82JH | |
| HLFT7C201A4636 | HLFT7C201BX0BF | HTNRPVY01ATSYH | HTNRPVY01AK9DH | |
| HLFT7C201BHK5A | HLFT7C201BI17G | HTNRPVY01BY5F6 | HTNRPVY01BBLMS | |
| HLFT7C201BN2X7 | HLFT7C201BIAQO | HTNRPVY01AGVK2 | HTNRPVY01A727W | |
| HLFT7C201A09SV | HLFT7C201BHR34 | HTNRPVY01BUE9W | HTNRPVY01BBYEL | |
| HLFT7C201ALNFB | HLFT7C201AJ1CK | HTNRPVY01BDDMP | HTNRPVY01AE388 | |
| HLFT7C201BD3DB | HLFT7C201BLSRA | HTNRPVY01A6FL9 | HTNRPVY01AP0ZJ | |
| HLFT7C201BERCQ | HLFT7C201B23QP | HTNRPVY01ADGI4 | HTNRPVY01AFTZY | |
| HLFT7C201AU3ZH | HLFT7C201ALO7V | HTNRPVY01A0QQG | HTNRPVY01BQ2HX | |
| HLFT7C201AH67U | HLFT7C201AT1X3 | HTNRPVY01ABVFZ | HTNRPVY01BBITP | |
| HLFT7C201B09FL | HLFT7C201ABY91 | HTNRPVY01AISXO | HTNRPVY01BUKKW | |
| HLFT7C201ARCP0 | HLFT7C201AV8XT | HTNRPVY01BA28T | HTNRPVY01A3JCQ | |
| HLFT7C201A0DNM | HLFT7C201A0DZB | HTNRPVY01ABR73 | HTNRPVY01BL42G | |
| HLFT7C201AATM4 | HLFT7C201A6SOQ | HTNRPVY01ALIXE | HTNRPVY01AFKRL | |
| HLFT7C201AHUGV | HLFT7C201ANNDN | HTNRPVY01BCNKQ | HTNRPVY01AULBI | |
| HLFT7C201B1VAY | HLFT7C201ABFEH | HTNRPVY01A9VO0 | HTNRPVY01AVM48 | |
| HLFT7C201AZBF6 | HLFT7C201ANQV7 | HTNRPVY01BVV9I | HTNRPVY01BZQ89 | |
| HLFT7C201A24G4 | HLFT7C201AKNQ6 | HTNRPVY01AAQN7 | HTNRPVY01BKWLI | |
| HLFT7C201AP31Y | HLFT7C201AHKN6 | HTNRPVY01A2UDY | HTNRPVY01AL5MT | |
| HLFT7C201AOSD5 | HLFT7C201BJUW7 | HTNRPVY01BJ4W5 | HTNRPVY01AG1W6 | |
| HLFT7C201A17IT | HLFT7C201BYYJ3 | HTNRPVY01A76TP | HTNRPVY01A854T | |
| HLFT7C201AN6DX | HLFT7C201AB766 | HTNRPVY01AA6TA | HTNRPVY01BWOM0 | |
| HLFT7C201B1VA5 | HLFT7C201BUNIZ | HTNRPVY01AMBRX | HTNRPVY01APG7Y | |
| HLFT7C201AP0P6 | HLFT7C201BOVPM | HTNRPVY01BL422 | HTNRPVY01ARYCJ | |
| HLFT7C201AYL7M | HLFT7C201A79TK | HTNRPVY01BEVKP | HTNRPVY01BO7TU | |
| HLFT7C201BMKKB | HLFT7C201AFEGC | HTNRPVY01ALMD0 | HTNRPVY01BIJRN | |
| HLFT7C201AOLN5 | HLFT7C201AEE6E | HTNRPVY01BUQPL | HTNRPVY01A4K2H | |
| HLFT7C201AW1DF | HLFT7C201ATIXU | HTNRPVY01APZ88 | HTNRPVY01AJE0R | |
| HLFT7C201A4VNA | HLFT7C201BPK0E | HTNRPVY01BDGV0 | HTNRPVY01B1I93 | |
| HLFT7C201AXXQJ | HLFT7C201AX22F | HTNRPVY01BWIL2 | HTNRPVY01BNAZC | |
| HLFT7C201BTVCB | HLFT7C201AGDBI | HTNRPVY01A9783 | HTNRPVY01A7KO6 | |
| HLFT7C201BFNOU | HLFT7C201A67JA | HTNRPVY01AGGPK | HTNRPVY01BD81M | |
| HLFT7C201AGL0C | HLFT7C201A3TJA | HTNRPVY01ACISK | HTNRPVY01BN9IW | |
| HLFT7C201B0FY6 | HLFT7C201BLI5T | HTNRPVY01AD5SJ | HTNRPVY01BK9A5 | |
| HLFT7C201BIM76 | HLFT7C201BQVS1 | HTNRPVY01AERPH | HTNRPVY01AXHQF | |
| HLFT7C201B20KH | HLFT7C201BDDB1 | HTNRPVY01BC7KD | HTNRPVY01AKKV5 | |
| HLFT7C201BE35P | HLFT7C201ABF7L | HTNRPVY01BRBDK | HTNRPVY01ANQ2V | |
| HLFT7C201BP4DV | HLFT7C201AU65M | HTNRPVY01BS55E | HTNRPVY01BGC40 | |
| HLFT7C201AJO12 | HLFT7C201BJO86 | HTNRPVY01A0G3U | HTNRPVY01ACERN | |
| HLFT7C201A31X9 | HLFT7C201AS0CC | HTNRPVY01AIZ3C | HTNRPVY01BF5YA | |
| HLFT7C201ANG8U | HLFT7C201A8VYV | HTNRPVY01ALMGJ | HTNRPVY01BFMV0 | |
| HLFT7C201AB5IA | HLFT7C201BF50P | HTNRPVY01APRC3 | HTNRPVY01BDY6P | |
| HLFT7C201AE38X | HLFT7C201BKHPX | HTNRPVY01ABYXZ | HTNRPVY01B3F6X | |
| HLFT7C201AXOHR | HLFT7C201AZEVY | HTNRPVY01BK34A | HTNRPVY01AXA7U | |
| HLFT7C201A00CO | HLFT7C201AN2M8 | HTNRPVY01AF6UD | HTNRPVY01A5DZ9 | |
| HLFT7C201AXQE9 | HLFT7C201B17UI | HTNRPVY01BKJ2L | HTNRPVY01BKQBD | |
| HLFT7C201AJRZ4 | HLFT7C201AN9R9 | HTNRPVY01BQ561 | HTNRPVY01ADTEC | |
| HLFT7C201AY7YT | HLFT7C201AKKDM | HTNRPVY01BGSVU | HTNRPVY01BZLC0 | |
| HLFT7C201AC64K | HLFT7C201BCA25 | HTNRPVY01APW2S | HTNRPVY01BZ3Y1 | |
| HLFT7C201AEOBN | HLFT7C201A46SB | HTNRPVY01BFRA3 | HTNRPVY01BWN1L | |
| HLFT7C201AFA8S | HLFT7C201BT4KL | HTNRPVY01ALZSI | HTNRPVY01AR66N | |
| HLFT7C201BEEUB | HLFT7C201BXDUZ | HTNRPVY01B2P5P | HTNRPVY01A85QS | |
| HLFT7C201BZNK0 | HLFT7C201AE1UD | HTNRPVY01AWOBB | HTNRPVY01AJR0P | |
| HLFT7C201BMH2Y | HLFT7C201AH4OK | HTNRPVY01BXHXO | HTNRPVY01AMXM1 | |
| HLFT7C201AA938 | HLFT7C201AXWR8 | HTNRPVY01AHVIH | HTNRPVY01AFTVA | |
| HLFT7C201BLTDH | HLFT7C201AZIA7 | HTNRPVY01BDVOX | HTNRPVY01ALGP2 | |
| HLFT7C201AMI17 | HLFT7C201AQYY5 | HTNRPVY01BQF7C | HTNRPVY01BSJID | |
| HLFT7C201B2HOH | HLFT7C201A224L | HTNRPVY01BOZFM | HTNRPVY01AOCWX | |
| HLFT7C201ACESE | HLFT7C201BXTZG | HTNRPVY01BP0W4 | HTNRPVY01BND8I | |
| HLFT7C201AYFS6 | HLFT7C201A8TQ4 | HTNRPVY01AB2FW | HTNRPVY01BVPE8 | |
| HLFT7C201AYZMS | HLFT7C201AEU0L | HTNRPVY01AEFRY | HTNRPVY01A3YS6 | |
| HLFT7C201A3GR1 | HLFT7C201ADJYK | HTNRPVY01AZXJN | HTNRPVY01ANDY1 | |
| HLFT7C201BZ6I3 | HLFT7C201AE7RN | HTNRPVY01A67HX | HTNRPVY01BKKJ4 | |
| HLFT7C201AWB5M | HLFT7C201BCEOG | HTNRPVY01BLWAA | HTNRPVY01BSNJC | |
| HLFT7C201B0FY0 | HLFT7C201BR7FV | HTNRPVY01BQ2U1 | HTNRPVY01BMKWE | |
| HLFT7C201AN5RX | HLFT7C201BNAK6 | HTNRPVY01BCK1W | HTNRPVY01AZNY5 | |
| HLFT7C201BGO5Z | HLFT7C201BV1Z4 | HTNRPVY01BUQTK | HTNRPVY01AYWEL | |
| HLFT7C201BSNIB | HLFT7C201BIAFV | HTNRPVY01B3MYS | HTNRPVY01BAV7R | |
| HLFT7C201AXZ7X | HLFT7C201AFKK9 | HTNRPVY01BPUXY | HTNRPVY01AGJYY | |
| HLFT7C201AX20U | HLFT7C201BUTTY | HTNRPVY01AY8TB | HTNRPVY01BFJY4 | |
| HLFT7C201AXK7R | HLFT7C201BWO2X | HTNRPVY01AZUHJ | HTNRPVY01BBVYK | |
| HLFT7C201BY102 | HLFT7C201BUKRP | HTNRPVY01BV9HO | HTNRPVY01B1U9K | |
| HLFT7C201BKQQ7 | HLFT7C201AK6JI | HTNRPVY01A528W | HTNRPVY01A1YS8 | |
| HLFT7C201A6IB0 | HLFT7C201A6LOE | HTNRPVY01BAAX5 | HTNRPVY01A5851 | |
| HLFT7C201BKUOX | HLFT7C201A58X4 | HTNRPVY01A5EHQ | HTNRPVY01ADP09 | |
| HLFT7C201BN6PR | HLFT7C201A293M | HTNRPVY01AMN9K | HTNRPVY01AZIE0 | |
| HLFT7C201ALZ1R | HLFT7C201B1U5K | HTNRPVY01AQYW5 | HTNRPVY01ASEMX | |
| HLFT7C201AVTW9 | HLFT7C201AQS0O | HTNRPVY01AG2NR | HTNRPVY01AZRM7 | |
| HLFT7C201ANZSP | HLFT7C201ANTAS | HTNRPVY01BBSBU | HTNRPVY01A6X9F | |
| HLFT7C201AH1VQ | HLFT7C201AD5Y9 | HTNRPVY01AO5Q2 | HTNRPVY01ATL4C | |
| HLFT7C201AI0ME | HLFT7C201BWRUR | HTNRPVY01BP6C2 | HTNRPVY01BU35X | |
| HLFT7C201A1SSD | HLFT7C201ASHJY | HTNRPVY01BSGCC | HTNRPVY01BM4DL | |
| HLFT7C201BH7CQ | HLFT7C201BAZ5M | HTNRPVY01AKHY2 | HTNRPVY01AHQ1P | |
| HLFT7C201BSKT8 | HLFT7C201BBIYL | HTNRPVY01B0MHH | HTNRPVY01A8ND0 | |
| HLFT7C201BBO7R | HLFT7C201BI2M4 | HTNRPVY01BUKLT | HTNRPVY01BM62A | |
| HLFT7C201B0ZJU | HLFT7C201BLYG1 | HTNRPVY01BIAO4 | HTNRPVY01AV9M0 | |
| HLFT7C201AGZBR | HLFT7C201BSZH0 | HTNRPVY01ALJVR | HTNRPVY01BSC5I | |
| HLFT7C201ABFSU | HLFT7C201BDJ3M | HTNRPVY01BEURL | HTNRPVY01AOB5Y | |
| HLFT7C201ALCQV | HLFT7C201B17TB | HTNRPVY01AKDWE | HTNRPVY01A5D39 | |
| HLFT7C201BV8A7 | HLFT7C201BZQ22 | HTNRPVY01BXW80 | HTNRPVY01ATF77 | |
| HLFT7C201A9Q59 | HLFT7C201BIQ1X | HTNRPVY01BFDED | HTNRPVY01A6PHP | |
| HLFT7C201AAWX6 | HLFT7C201AGMR3 | HTNRPVY01AEISE | HTNRPVY01BUKB0 | |
| HLFT7C201BADJE | HLFT7C201BWER2 | HTNRPVY01AQ9KU | HTNRPVY01A8PAJ | |
| HLFT7C201AB1JY | HLFT7C201AK9Z4 | HTNRPVY01BIV8C | HTNRPVY01AYI1W | |
| HLFT7C201BT4MR | HLFT7C201AC3H5 | HTNRPVY01A43R7 | HTNRPVY01ABVZ3 | |
| HLFT7C201BNTLU | HLFT7C201ASECE | HTNRPVY01A1RUO | HTNRPVY01ALF23 | |
| HLFT7C201BYMJ0 | HLFT7C201AGL9Z | HTNRPVY01A97JZ | HTNRPVY01AHK5T | |
| HLFT7C201BC3XK | HLFT7C201AIDQ1 | HTNRPVY01A6X6P | HTNRPVY01A3WKN | |
| HLFT7C201A3MGG | HLFT7C201AAJXT | HTNRPVY01BKN29 | HTNRPVY01ANTUQ | |
| HLFT7C201AQDZF | HLFT7C201BPUDQ | HTNRPVY01AT7S1 | HTNRPVY01BCA01 | |
| HLFT7C201AZHY0 | HLFT7C201AL4YV | HTNRPVY01A71D3 | HTNRPVY01AQG8P | |
| HLFT7C201ADGML | HLFT7C201BAJZQ | HTNRPVY01A41X7 | HTNRPVY01AM4BW | |
| HLFT7C201ADWH7 | HLFT7C201BPKS1 | HTNRPVY01BBYAZ | HTNRPVY01AUQ2E | |
| HLFT7C201AJCWA | HLFT7C201AOSBZ | HTNRPVY01A343M | HTNRPVY01AW7SO | |
| HLFT7C201AOYDQ | HLFT7C201BNJGB | HTNRPVY01BITHI | HTNRPVY01AOYES | |
| HLFT7C201AMK76 | HLFT7C201BPK3F | HTNRPVY01AF3H7 | HTNRPVY01A2235 | |
| HLFT7C201A6YZ1 | HLFT7C201AE7F3 | HTNRPVY01BUHHK | HTNRPVY01BAT2D | |
| HLFT7C201BAEIJ | HLFT7C201AOCTK | HTNRPVY01AWVTB | HTNRPVY01BQ5J6 | |
| HLFT7C201BLVIG | HLFT7C201A6139 | HTNRPVY01BAWRJ | HTNRPVY01AAZ7I | |
| HLFT7C201ATDC5 | HLFT7C201BP0TC | HTNRPVY01BO06T | HTNRPVY01AIC4W | |
| HLFT7C201BD8T6 | HLFT7C201A0Y5X | HTNRPVY01AOR2I | HTNRPVY01ASWMQ | |
| HLFT7C201AXGPF | HLFT7C201BJC6O | HTNRPVY01APQ34 | HTNRPVY01APN6T | |
| HLFT7C201AMSKK | HLFT7C201AKWYX | HTNRPVY01B3AD0 | HTNRPVY01BLCEV | |
| HLFT7C201ASJS3 | HLFT7C201BQWRN | HTNRPVY01BHW42 | HTNRPVY01BEL6P | |
| HLFT7C201ARLVD | HLFT7C201AWOO1 | HTNRPVY01APH01 | HTNRPVY01A06LV | |
| HLFT7C201BSGZJ | HLFT7C201AL5UE | HTNRPVY01AVDOH | HTNRPVY01AQ5XF | |
| HLFT7C201BT5T4 | HLFT7C201BHEX3 | HTNRPVY01AEY7O | HTNRPVY01ALPNR | |
| HLFT7C201AJ8UG | HLFT7C201BUXEQ | HTNRPVY01AYY8M | HTNRPVY01ADGK3 | |
| HLFT7C201BNJOD | HLFT7C201AXQM8 | HTNRPVY01AKQOU | HTNRPVY01A5T1S | |
| HLFT7C201BCN1J | HLFT7C201BF0P9 | HTNRPVY01B23BI | HTNRPVY01BEPCJ | |
| HLFT7C201A5V4B | HLFT7C201ANQPI | HTNRPVY01BI88F | HTNRPVY01ALSYO | |
| HLFT7C201AOMVT | HLFT7C201A1SH7 | HTNRPVY01BH00P | HTNRPVY01AWUXC | |
| HLFT7C201A254Z | HLFT7C201B3ZD5 | HTNRPVY01AYTJP | HTNRPVY01BEIVB | |
| HLFT7C201AFQZI | HLFT7C201ARBOA | HTNRPVY01AXXEI | HTNRPVY01BNP6S | |
| HLFT7C201BG15S | HLFT7C201ACEOI | HTNRPVY01AF284 | HTNRPVY01BBMJS | |
| HLFT7C201A46YN | HLFT7C201AK7AQ | HTNRPVY01AWVC9 | HTNRPVY01AL8OL | |
| HLFT7C201BKZ9P | HLFT7C201AF3R5 | HTNRPVY01BJ1WO | HTNRPVY01A5GH9 | |
| HLFT7C201ARLNT | HLFT7C201BF9TO | HTNRPVY01BSTMQ | HTNRPVY01BWYC2 | |
| HLFT7C201AR18F | HLFT7C201BV2XF | HTNRPVY01A2M5Z | HTNRPVY01AVP9B | |
| HLFT7C201BGR21 | HLFT7C201APXJV | HTNRPVY01A7BST | HTNRPVY01AT18W | |
| HLFT7C201BKKD8 | HLFT7C201AIV80 | HTNRPVY01AHUT3 | HTNRPVY01BHH75 | |
| HLFT7C201AYL2B | HLFT7C201AFUHH | HTNRPVY01APH5F | HTNRPVY01A9UOZ | |
| HLFT7C201ARUGT | HLFT7C201BAECI | HTNRPVY01AGZTA | HTNRPVY01AYPGF | |
| HLFT7C201A5JFA | HLFT7C201BWLT1 | HTNRPVY01BTILP | HTNRPVY01AE7Z4 | |
| HLFT7C201BXMXF | HLFT7C201A85X5 | HTNRPVY01A5DB1 | HTNRPVY01A965W | |
| HLFT7C201AKXGV | HLFT7C201AOIXL | HTNRPVY01AGDBY | HTNRPVY01AGF12 | |
| HLFT7C201BEOB5 | HLFT7C201A3CF2 | HTNRPVY01BJO54 | HTNRPVY01AN5V0 | |
| HLFT7C201AJ7NT | HLFT7C201BXD8G | HTNRPVY01BA64J | HTNRPVY01BNAO9 | |
| HLFT7C201B3R66 | HLFT7C201BC9YH | HTNRPVY01AXWRW | HTNRPVY01AXP45 | |
| HLFT7C201BE1EW | HLFT7C201AH6TE | HTNRPVY01BBI8J | HTNRPVY01A38LJ | |
| HLFT7C201A3ZSI | HLFT7C201AUNN1 | HTNRPVY01AITAY | HTNRPVY01AGSQ3 | |
| HLFT7C201BOUT5 | HLFT7C201A5F91 | HTNRPVY01ACEE8 | HTNRPVY01A1IVP | |
| HLFT7C201AEE89 | HLFT7C201BPW6U | HTNRPVY01BOYHE | HTNRPVY01AISW0 | |
| HLFT7C201BXZMH | HLFT7C201BW7S1 | HTNRPVY01A2Q1N | HTNRPVY01A3QJS | |
| HLFT7C201BBL5P | HLFT7C201ALFOS | HTNRPVY01BOCIF | HTNRPVY01BHAZG | |
| HLFT7C201AMOTB | HLFT7C201BI8YK | HTNRPVY01ARRU9 | HTNRPVY01BXZKA | |
| HLFT7C201AH638 | HLFT7C201BIZTZ | HTNRPVY01BQZC3 | HTNRPVY01BXZHS | |
| HLFT7C201BBFTT | HLFT7C201B1CBE | HTNRPVY01BKHRX | HTNRPVY01A6JBC | |
| HLFT7C201ASHCR | HLFT7C201AB5SN | HTNRPVY01BE5HL | HTNRPVY01ABY2Y | |
| HLFT7C201ADNHM | HLFT7C201BGSRP | HTNRPVY01AY8N1 | HTNRPVY01AKHGH | |
| HLFT7C201B3R6O | HLFT7C201BLI80 | HTNRPVY01BQZOK | HTNRPVY01AKKOL | |
| HLFT7C201A4LCA | HLFT7C201AJL04 | HTNRPVY01AHA69 | HTNRPVY01AX85B | |
| HLFT7C201A819G | HLFT7C201AMRFN | HTNRPVY01A6RSI | HTNRPVY01BD9C9 | |
| HLFT7C201AQ5SR | HLFT7C201BEK1D | HTNRPVY01A0PXE | HTNRPVY01B0C4L | |
| HLFT7C201BXJNW | HLFT7C201AXBA6 | HTNRPVY01A0PME | HTNRPVY01AE5EL | |
| HLFT7C201AGCE3 | HLFT7C201BW02Z | HTNRPVY01AB1WY | HTNRPVY01AKJ7V | |
| HLFT7C201AV2H3 | HLFT7C201BNAFP | HTNRPVY01BG5V8 | HTNRPVY01AZ4X2 | |
| HLFT7C201A7TNU | HLFT7C201BE73Y | HTNRPVY01AL2MO | HTNRPVY01BWUCL | |
| HLFT7C201BL2UG | HLFT7C201ANARJ | HTNRPVY01A224R | HTNRPVY01BI0B7 | |
| HLFT7C201ANM3T | HLFT7C201A64US | HTNRPVY01ALJO2 | HTNRPVY01BBCN4 | |
| HLFT7C201BX6FD | HLFT7C201AXQE5 | HTNRPVY01AJVAX | HTNRPVY01BHOU3 | |
| HLFT7C201AJLWG | HLFT7C201AXKT8 | HTNRPVY01A08K1 | HTNRPVY01B2UKP | |
| HLFT7C201BFTGD | HLFT7C201A4EEA | HTNRPVY01A2QLF | HTNRPVY01A1VAG | |
| HLFT7C201A3MIU | HLFT7C201BS2T2 | HTNRPVY01BQJGH | HTNRPVY01A8CTR | |
| HLFT7C201A06PY | HLFT7C201BEXOL | HTNRPVY01A644R | HTNRPVY01AV2ZV | |
| HLFT7C201AUTWZ | HLFT7C201APZ6R | HTNRPVY01ADKKY | HTNRPVY01BNHEK | |
| HLFT7C201B2879 | HLFT7C201AQGVG | HTNRPVY01BI80K | HTNRPVY01A843I | |
| HLFT7C201AQ46U | HLFT7C201A8GNH | HTNRPVY01BIAUP | HTNRPVY01BANWD | |
| HLFT7C201BOGFC | HLFT7C201A2Q5K | HTNRPVY01AS2Q2 | HTNRPVY01AZRFY | |
| HLFT7C201B1K1R | HLFT7C201A4IEU | HTNRPVY01A3GA6 | HTNRPVY01BABFM | |
| HLFT7C201BQ15L | HLFT7C201A3DOE | HTNRPVY01AHT5S | HTNRPVY01A6U62 | |
| HLFT7C201AMX9I | HLFT7C201AQPJC | HTNRPVY01BV12D | HTNRPVY01BGDL9 | |
| HLFT7C201AA9GI | HLFT7C201BYJR9 | HTNRPVY01B05PG | HTNRPVY01ABGW2 | |
| HLFT7C201BOYLN | HLFT7C201BQDAZ | HTNRPVY01AB7Y3 | HTNRPVY01BLDL1 | |
| HLFT7C201ASHB2 | HLFT7C201A82F3 | HTNRPVY01B2GZ4 | HTNRPVY01BHLID | |
| HLFT7C201APW83 | HLFT7C201ACKOZ | HTNRPVY01A9LHI | HTNRPVY01AS97F | |
| HLFT7C201BYFU5 | HLFT7C201AOF18 | HTNRPVY01AY5MB | HTNRPVY01A47AH | |
| HLFT7C201AHER2 | HLFT7C201A8V3S | HTNRPVY01ARICI | HTNRPVY01AUN64 | |
| HLFT7C201BY47P | HLFT7C201BJX3C | HTNRPVY01BKA4P | HTNRPVY01BO4S8 | |
| HLFT7C201AP7SQ | HLFT7C201BXZLE | HTNRPVY01A6OYT | HTNRPVY01BKQNC | |
| HLFT7C201AST20 | HLFT7C201A41EA | HTNRPVY01BYFEO | HTNRPVY01AV8YO | |
| HLFT7C201ASEAV | HLFT7C201ANHNQ | HTNRPVY01BDV30 | HTNRPVY01A2QU8 | |
| HLFT7C201A29A6 | HLFT7C201BA3AY | HTNRPVY01BDZAT | HTNRPVY01ACIQ0 | |
| HLFT7C201BKAXD | HLFT7C201BRCQU | HTNRPVY01ABSM4 | HTNRPVY01BB2HY | |
| HLFT7C201B0CRB | HLFT7C201BYX8Q | HTNRPVY01A8KDZ | HTNRPVY01BOU0N | |
| HLFT7C201BCHS0 | HLFT7C201AQZBI | HTNRPVY01BQF9M | HTNRPVY01ALAG2 | |
| HLFT7C201ANHVC | HLFT7C201BAT0U | HTNRPVY01AL5PK | HTNRPVY01BM935 | |
| HLFT7C201AI2Y7 | HLFT7C201BLSF9 | HTNRPVY01B0CUO | HTNRPVY01AHUAT | |
| HLFT7C201BRNW4 | HLFT7C201BXQVI | HTNRPVY01BYIPO | HTNRPVY01BA2UW | |
| HLFT7C201BXNS7 | HLFT7C201A6VUK | HTNRPVY01B0Y4Y | HTNRPVY01BA3IC | |
| HLFT7C201AM1UW | HLFT7C201AFM5B | HTNRPVY01BWIF1 | HTNRPVY01BD2SR | |
| HLFT7C201BD83U | HLFT7C201B0P6Y | HTNRPVY01A7XAO | HTNRPVY01AI6LV | |
| HLFT7C201BIA81 | HLFT7C201AHIEG | HTNRPVY01A4N95 | HTNRPVY01AOZJR | |
| HLFT7C201BS561 | HLFT7C201ACHDE | HTNRPVY01AWYPN | HTNRPVY01BHX33 | |
| HLFT7C201A23OI | HLFT7C201A4E3F | HTNRPVY01BJF38 | HTNRPVY01AMH4M | |
| HLFT7C201BSMY9 | HLFT7C201BLF90 | HTNRPVY01BE8AI | HTNRPVY01ATJJI | |
| HLFT7C201BSBA2 | HLFT7C201AFG5S | HTNRPVY01AQ46V | HTNRPVY01BC9RS | |
| HLFT7C201BKZPG | HLFT7C201BGL74 | HTNRPVY01AOLQR | HTNRPVY01BLP41 | |
| HLFT7C201AU3PG | HLFT7C201BYSI4 | HTNRPVY01BZU59 | HTNRPVY01BDKEY | |
| HLFT7C201BJU4J | HLFT7C201BPUKX | HTNRPVY01BTI4O | HTNRPVY01BFZ3K | |
| HLFT7C201AI6C1 | HLFT7C201BD52U | HTNRPVY01BSS61 | HTNRPVY01AZQXF | |
| HLFT7C201A7RRK | HLFT7C201B3ZHH | HTNRPVY01BH7AL | HTNRPVY01ARRUY | |
| HLFT7C201AUE9Q | HLFT7C201BD5G2 | HTNRPVY01AJOR4 | HTNRPVY01BHQS7 | |
| HLFT7C201B3L0K | HLFT7C201BZENR | HTNRPVY01A2TZ0 | HTNRPVY01A43PN | |
| HLFT7C201BN3J8 | HLFT7C201BD5V2 | HTNRPVY01AUX8B | HTNRPVY01BC4E5 | |
| HLFT7C201AZW7X | HLFT7C201A6FYK | HTNRPVY01AKD1B | HTNRPVY01AX50R | |
| HLFT7C201BPXI0 | HLFT7C201BG5FG | HTNRPVY01AQJYH | HTNRPVY01BYVQ1 | |
| HLFT7C201BCB0R | HLFT7C201BVJYZ | HTNRPVY01AAJTT | HTNRPVY01A1YVN | |
| HLFT7C201APEON | HLFT7C201AAP4X | HTNRPVY01AMBB7 | HTNRPVY01A9UCT | |
| HLFT7C201ARCNY | HLFT7C201A0DEO | HTNRPVY01A58SD | HTNRPVY01ADAL1 | |
| HLFT7C201AN27F | HLFT7C201A65PA | HTNRPVY01ALMA4 | HTNRPVY01AGSHH | |
| HLFT7C201A6705 | HLFT7C201BNGIN | HTNRPVY01BADMA | HTNRPVY01AHBMA | |
| HLFT7C201AATR4 | HLFT7C201AWFJS | HTNRPVY01BV5BU | HTNRPVY01ANQ33 | |
| HLFT7C201AHH3X | HLFT7C201BQVHQ | HTNRPVY01BC6T7 | HTNRPVY01AHBHC | |
| HLFT7C201AZHXN | HLFT7C201A9X08 | HTNRPVY01ACK54 | HTNRPVY01AP0T2 | |
| HLFT7C201ANT0J | HLFT7C201BL5FU | HTNRPVY01BVPYJ | HTNRPVY01BKUD7 | |
| HLFT7C201AR4N0 | HLFT7C201BUQUB | HTNRPVY01BCYB2 | HTNRPVY01AGC9P | |
| HLFT7C201A50I4 | HLFT7C201BXDDC | HTNRPVY01BY4PX | HTNRPVY01AN3SY | |
| HLFT7C201A3211 | HLFT7C201AG5HK | HTNRPVY01AWJBA | HTNRPVY01A7XJN | |
| HLFT7C201AMHP6 | HLFT7C201BT4L6 | HTNRPVY01BYJHC | HTNRPVY01BMRJB | |
| HLFT7C201B2NUQ | HLFT7C201ANW14 | HTNRPVY01BOV5E | HTNRPVY01BFDID | |
| HLFT7C201A8JJ2 | HLFT7C201AX9MT | HTNRPVY01AVAO5 | HTNRPVY01AD27O | |
| HLFT7C201AMUUP | HLFT7C201BQFPG | HTNRPVY01AZ3TJ | HTNRPVY01BAALT | |
| HLFT7C201AMH7U | HLFT7C201AZRS8 | HTNRPVY01BQVUX | HTNRPVY01AOFH2 | |
| HLFT7C201ALGP4 | HLFT7C201BVSHK | HTNRPVY01BSWYI | HTNRPVY01AFBTK | |
| HLFT7C201BAS5M | HLFT7C201B05XK | HTNRPVY01BIS45 | HTNRPVY01AVAMK | |
| HLFT7C201AZHGS | HLFT7C201ASM3K | HTNRPVY01AJ41Y | HTNRPVY01AXKEI | |
| HLFT7C201AVZBL | HLFT7C201ABV7V | HTNRPVY01AX3WE | HTNRPVY01A05TE | |
| HLFT7C201AIQ3L | HLFT7C201BS57F | HTNRPVY01AIZBZ | HTNRPVY01A23CQ | |
| HLFT7C201A2WY2 | HLFT7C201BROQV | HTNRPVY01AUIGM | HTNRPVY01ABSCX | |
| HLFT7C201BRA75 | HLFT7C201ALG0M | HTNRPVY01AYSIB | HTNRPVY01BXDJC | |
| HLFT7C201AGSBB | HLFT7C201ASDVY | HTNRPVY01AINBM | HTNRPVY01AXN7O | |
| HLFT7C201AT81V | HLFT7C201A4NU5 | HTNRPVY01BZYHN | HTNRPVY01A20C7 | |
| HLFT7C201BIMGR | HLFT7C201AATML | HTNRPVY01BQ2AV | HTNRPVY01AL530 | |
| HLFT7C201AJ2JU | HLFT7C201BZEWN | HTNRPVY01A1B7P | HTNRPVY01A5HFR | |
| HLFT7C201BQMWP | HLFT7C201BOLLS | HTNRPVY01AEF12 | HTNRPVY01BT1CF | |
| HLFT7C201A7DUE | HLFT7C201B0TI3 | HTNRPVY01B3PQP | HTNRPVY01BPN8L | |
| HLFT7C201ARK7T | HLFT7C201BNM7O | HTNRPVY01AOMND | HTNRPVY01ABYCF | |
| HLFT7C201BBPBW | HLFT7C201A20RK | HTNRPVY01AKEG4 | HTNRPVY01A0Y45 | |
| HLFT7C201BSGVS | HLFT7C201A3FQH | HTNRPVY01AH91M | HTNRPVY01ATB65 | |
| HLFT7C201AZ60C | HLFT7C201AIACD | HTNRPVY01AIWLY | HTNRPVY01A4OL3 | |
| HLFT7C201BZ7B9 | HLFT7C201BMNQJ | HTNRPVY01BAKP9 | HTNRPVY01BERZH | |
| HLFT7C201A94RZ | HLFT7C201BCKJO | HTNRPVY01AKRTV | HTNRPVY01BHG88 | |
| HLFT7C201BT7OB | HLFT7C201BOEZ4 | HTNRPVY01BV2T5 | HTNRPVY01BTB95 | |
| HLFT7C201AI8ZJ | HLFT7C201AHN1J | HTNRPVY01AVC73 | HTNRPVY01BYCM7 | |
| HLFT7C201A8ZH2 | HLFT7C201A03KN | HTNRPVY01AU0B1 | HTNRPVY01AYGLK | |
| HLFT7C201A3LWW | HLFT7C201A2WPK | HTNRPVY01BDD61 | HTNRPVY01AHNXR | |
| HLFT7C201AO4JT | HLFT7C201BLY9A | HTNRPVY01BHH43 | HTNRPVY01BJPED | |
| HLFT7C201BFJS8 | HLFT7C201BFTVL | HTNRPVY01A3C34 | HTNRPVY01BC7X6 | |
| HLFT7C201A816S | HLFT7C201A40JG | HTNRPVY01AVTEU | HTNRPVY01BSTK2 | |
| HLFT7C201BU1GG | HLFT7C201AHX93 | HTNRPVY01AMRJJ | HTNRPVY01AQZXE | |
| HLFT7C201AC0TI | HLFT7C201BQSD8 | HTNRPVY01AN6BS | HTNRPVY01AGS4U | |
| HLFT7C201ACUMT | HLFT7C201AOOPF | HTNRPVY01A1C42 | HTNRPVY01A7BXY | |
| HLFT7C201AOSC2 | HLFT7C201AC0E9 | HTNRPVY01AG8RL | HTNRPVY01AWRGO | |
| HLFT7C201AIJ5K | HLFT7C201A1YXZ | HTNRPVY01BMD9U | HTNRPVY01BQTS6 | |
| HLFT7C201BHHPO | HLFT7C201AQP3V | HTNRPVY01AH7HR | HTNRPVY01BS6KT | |
| HLFT7C201BXHFO | HLFT7C201AKDVN | HTNRPVY01AYPVJ | HTNRPVY01A7KA8 | |
| HLFT7C201BPW3Q | HLFT7C201ABS0Q | HTNRPVY01ADS33 | HTNRPVY01BVPYF | |
| HLFT7C201A70BN | HLFT7C201AC6OI | HTNRPVY01BV20E | HTNRPVY01BG8RW | |
| HLFT7C201BZXEZ | HLFT7C201BYFGN | HTNRPVY01AUB19 | HTNRPVY01A0T3S | |
| HLFT7C201BIJPQ | HLFT7C201AIAL7 | HTNRPVY01AP62S | HTNRPVY01B2KB9 | |
| HLFT7C201BHN15 | HLFT7C201BGVB1 | HTNRPVY01BAK1K | HTNRPVY01AFDTT | |
| HLFT7C201AK62V | HLFT7C201AIP2Q | HTNRPVY01A1R06 | HTNRPVY01AN6V7 | |
| HLFT7C201BFNAH | HLFT7C201AWU1V | HTNRPVY01AP0I1 | HTNRPVY01BAHKM | |
| HLFT7C201BERW4 | HLFT7C201AZU1O | HTNRPVY01BXA96 | HTNRPVY01BM332 | |
| HLFT7C201BY4LO | HLFT7C201AE7VN | HTNRPVY01B2RAK | HTNRPVY01B2TYS | |
| HLFT7C201BYILS | HLFT7C201A1BQY | HTNRPVY01AI85X | HTNRPVY01BW0WZ | |
| HLFT7C201ANDZX | HLFT7C201AWVIG | HTNRPVY01BT1GB | HTNRPVY01B1XXV | |
| HLFT7C201BGVLY | HLFT7C201BZQ0O | HTNRPVY01A9L8G | HTNRPVY01B3MO8 | |
| HLFT7C201AGCUC | HLFT7C201BH99G | HTNRPVY01A8WA6 | HTNRPVY01AJYMM | |
| HLFT7C201BBE6H | HLFT7C201B2XIL | HTNRPVY01BAG0E |  |  |
| HLFT7C201AMK0Y | HLFT7C201AC333 | HTNRPVY01A8I7F |  |  |
| HLFT7C201ARB2J | HLFT7C201BBSRN | HTNRPVY01BHT18 |  |  |
| HLFT7C201A7N16 | HLFT7C201AOB54 | HTNRPVY01AIKTA |  |  |
| HLFT7C201ASQ88 | HLFT7C201AU9RP | HTNRPVY01A8JUK |  |  |
| HLFT7C201AANDM | HLFT7C201BGL57 | HTNRPVY01BAW05 |  |  |
| HLFT7C201ATVHL | HLFT7C201AK0NX | HTNRPVY01BVL9T |  |  |
| HLFT7C201B2KGV | HLFT7C201ARBUF | HTNRPVY01BS5L7 |  |  |
| HLFT7C201BCAXM | HLFT7C201A2EJ1 | HTNRPVY01BNW1G |  |  |
| HLFT7C201A44DE | HLFT7C201AMX14 | HTNRPVY01AJ4SM |  |  |
| HLFT7C201BWK6Y | HLFT7C201A46XL | HTNRPVY01BDNAB |  |  |
| HLFT7C201AFG64 | HLFT7C201BNZGY | HTNRPVY01AFJYH |  |  |
| HLFT7C201ALJIN | HLFT7C201BR376 | HTNRPVY01A3FNH |  |  |
| HLFT7C201A02QV | HLFT7C201BD9BD | HTNRPVY01BBIGT |  |  |
| HLFT7C201B2T5A | HLFT7C201AKUKH | HTNRPVY01A8IWF |  |  |
| HLFT7C201A7UBE | HLFT7C201BJ8BH | HTNRPVY01BZRD6 |  |  |
| HLFT7C201BAWXR | HLFT7C201AK0IO | HTNRPVY01BXZ76 |  |  |
| HLFT7C201AJZBM | HLFT7C201ARE8K | HTNRPVY01BF9BJ |  |  |
| HLFT7C201A41CU | HLFT7C201ATPZJ | HTNRPVY01AC015 |  |  |
| HLFT7C201AVS5U | HLFT7C201AFNKX | HTNRPVY01AYIWH |  |  |
| HLFT7C201AANBC | HLFT7C201AA918 | HTNRPVY01AOVNW |  |  |
| HLFT7C201BGFLM | HLFT7C201A793T | HTNRPVY01AJ4BL |  |  |
| HLFT7C201AHIFY | HLFT7C201BZD35 | HTNRPVY01A29NL |  |  |
| HLFT7C201AEL1X | HLFT7C201BMORO | HTNRPVY01AZ7BT |  |  |
| HLFT7C201BJ4W2 | HLFT7C201BSZF0 | HTNRPVY01AKOG2 |  |  |
| HLFT7C201BQPR2 | HLFT7C201AVS6W | HTNRPVY01AWLRS |  |  |
| HLFT7C201A8I8T | HLFT7C201AF9L2 | HTNRPVY01AYVKK |  |  |
| HLFT7C201BTLT1 | HLFT7C201BJLU0 | HTNRPVY01BEH3D |  |  |
| HLFT7C201B0SLT | HLFT7C201AAHXK | HTNRPVY01AL9AN |  |  |
| HLFT7C201BWLPW | HLFT7C201BLMTD | HTNRPVY01ALSYD |  |  |
| HLFT7C201BRHQK | HLFT7C201AOI9V | HTNRPVY01BAZJU |  |  |
| HLFT7C201A3VZX | HLFT7C201A0GMA | HTNRPVY01AT5H2 |  |  |
| HLFT7C201BAQ3K | HLFT7C201A727V | HTNRPVY01BLY5D |  |  |
| HLFT7C201BJIHU | HLFT7C201ARCBH | HTNRPVY01A88NY |  |  |
| HLFT7C201BI6N1 | HLFT7C201BF0T5 | HTNRPVY01ASELV |  |  |
| HLFT7C201A3PJ0 | HLFT7C201BSQKF | HTNRPVY01A8WRH |  |  |
| HLFT7C201A1OYN | HLFT7C201A0AD7 | HTNRPVY01BMLBW |  |  |
| HLFT7C201ANEGM | HLFT7C201B2N3V | HTNRPVY01AW341 |  |  |
| HLFT7C201BJ1T2 | HLFT7C201ACN23 | HTNRPVY01A23BV |  |  |
| HLFT7C201A2976 | HLFT7C201A1R5Q | HTNRPVY01BR09G |  |  |
| HLFT7C201A0NSX | HLFT7C201BWLT4 | HTNRPVY01BWRRS |  |  |
| HLFT7C201BJMNC | HLFT7C201A44AP | HTNRPVY01BI9A0 |  |  |
| HLFT7C201BH3JR | HLFT7C201AGF7C | HTNRPVY01BRYLU |  |  |
| HLFT7C201BI16V | HLFT7C201AIT7W | HTNRPVY01BCN5T |  |  |
| HLFT7C201A3L5Q | HLFT7C201A1ODV | HTNRPVY01BOINL |  |  |
| HLFT7C201AHHTQ | HLFT7C201BIDUW | HTNRPVY01AM78A |  |  |
| HLFT7C201AADSJ | HLFT7C201BIVXY | HTNRPVY01A94KW |  |  |
| HLFT7C201B1IF6 | HLFT7C201ASM0X | HTNRPVY01AITWX |  |  |
| HLFT7C201BKAQI | HLFT7C201BN6D6 | HTNRPVY01AIKMN |  |  |
| HLFT7C201B3JJ8 | HLFT7C201A176C | HTNRPVY01A4B15 |  |  |
| HLFT7C201BT474 | HLFT7C201AC6LZ | HTNRPVY01AIWVJ |  |  |
| HLFT7C201BW315 | HLFT7C201AUXG5 | HTNRPVY01A3SDW |  |  |
| HLFT7C201AB1YH | HLFT7C201BPGWU | HTNRPVY01BATPR |  |  |
| HLFT7C201AKD85 | HLFT7C201BZXJA | HTNRPVY01ASAXN |  |  |
| HLFT7C201A6BVO | HLFT7C201BHKWM | HTNRPVY01BX5TW |  |  |
| HLFT7C201AQMXX | HLFT7C201AUA0S | HTNRPVY01AAT1O |  |  |
| HLFT7C201ASZVU | HLFT7C201BBY0N | HTNRPVY01BU6PQ |  |  |
| HLFT7C201BZW2O | HLFT7C201AJE6Y | HTNRPVY01AE4AZ |  |  |
| HLFT7C201BDZPP | HLFT7C201BYI2F | HTNRPVY01BWBJZ |  |  |
| HLFT7C201AFTHJ | HLFT7C201A9FFZ | HTNRPVY01AG9EE |  |  |
| HLFT7C201AC90A | HLFT7C201BPXEB | HTNRPVY01BRH5G |  |  |
| HLFT7C201A29VZ | HLFT7C201A9XME | HTNRPVY01BY1LX |  |  |
| HLFT7C201A3SWW | HLFT7C201AJI71 | HTNRPVY01BP6J5 |  |  |
| HLFT7C201BPUZE | HLFT7C201AJRXU | HTNRPVY01BCE72 |  |  |
| HLFT7C201AIY65 | HLFT7C201AK32R | HTNRPVY01AHKPE |  |  |
| HLFT7C201A6UVD | HLFT7C201BHBUV | HTNRPVY01ACUTO |  |  |
| HLFT7C201AJ4VD | HLFT7C201BELH4 | HTNRPVY01A89BE |  |  |
| HLFT7C201BZESF | HLFT7C201AS0D1 | HTNRPVY01AA5XV |  |  |
| HLFT7C201BN6OR | HLFT7C201A6B1X | HTNRPVY01AE7UL |  |  |
| HLFT7C201BJ2EI | HLFT7C201AGWSD | HTNRPVY01A5A6U |  |  |
| HLFT7C201ACQ7J | HLFT7C201AKD5Q | HTNRPVY01BLSXG |  |  |
| HLFT7C201AS3VN | HLFT7C201BBFUF | HTNRPVY01BYMNP |  |  |
| HLFT7C201A087L | HLFT7C201A85DI | HTNRPVY01ALDHW |  |  |
| HLFT7C201AM31U | HLFT7C201A2ARF | HTNRPVY01A1R9R |  |  |
| HLFT7C201BG5T1 | HLFT7C201BW1FS | HTNRPVY01BTUXR |  |  |
| HLFT7C201A4Q6A | HLFT7C201BH90D | HTNRPVY01AF9PN |  |  |
| HLFT7C201AISYA | HLFT7C201ASRDG | HTNRPVY01BVV13 |  |  |
| HLFT7C201BSGKQ | HLFT7C201BO7JY | HTNRPVY01AM65H |  |  |
| HLFT7C201ADJQ3 | HLFT7C201BQCH9 | HTNRPVY01BTF30 |  |  |
| HLFT7C201ATO49 | HLFT7C201A5AWN | HTNRPVY01BWFHX |  |  |
| HLFT7C201BFQQZ | HLFT7C201BFXBQ | HTNRPVY01BJLG0 |  |  |
| HLFT7C201AQC4Q | HLFT7C201BHH34 | HTNRPVY01BR4PF |  |  |
| HLFT7C201BW31X | HLFT7C201BCH4Q | HTNRPVY01ACNV3 |  |  |
| HLFT7C201AU6O7 | HLFT7C201A7Z33 | HTNRPVY01ALIWQ |  |  |
| HLFT7C201BOU64 | HLFT7C201AIDYA | HTNRPVY01AS3UV |  |  |
| HLFT7C201BV5QP | HLFT7C201BKJ2V | HTNRPVY01BI8KL |  |  |
| HLFT7C201AUB4S | HLFT7C201AC0W2 | HTNRPVY01B0TEI |  |  |
| HLFT7C201BMYJR | HLFT7C201A3MLM | HTNRPVY01ARE22 |  |  |
| HLFT7C201A85QT | HLFT7C201AXNVD | HTNRPVY01AI2EF |  |  |
| HLFT7C201AOLT6 | HLFT7C201AQSXY | HTNRPVY01BUEY3 |  |  |
| HLFT7C201AU0I5 | HLFT7C201BKN3A | HTNRPVY01ASNR6 |  |  |
| HLFT7C201AO4VN | HLFT7C201AVMFA | HTNRPVY01AP1DR |  |  |
| HLFT7C201BCQ23 | HLFT7C201AJ78Q | HTNRPVY01BB5VZ |  |  |
| HLFT7C201BFHER | HLFT7C201AJYS7 | HTNRPVY01BRLLR |  |  |
| HLFT7C201AAZ30 | HLFT7C201BVYQ7 | HTNRPVY01AKQTB |  |  |
| HLFT7C201BEUR7 | HLFT7C201BA602 | HTNRPVY01AOCRD |  |  |
| HLFT7C201AP3CL | HLFT7C201AU9TH | HTNRPVY01ABVBA |  |  |
| HLFT7C201ACUKT | HLFT7C201BOU7Y | HTNRPVY01BINQI |  |  |
| HLFT7C201AQ2M1 | HLFT7C201BT45C | HTNRPVY01AA9XX |  |  |
| HLFT7C201A3S23 | HLFT7C201AOCPA | HTNRPVY01ACBX7 |  |  |
| HLFT7C201BPTR1 | HLFT7C201BPBEU | HTNRPVY01AC6ZJ |  |  |
| HLFT7C201BIZ7K | HLFT7C201ABPKV | HTNRPVY01AZ736 |  |  |
| HLFT7C201BLYRT | HLFT7C201AOZD0 | HTNRPVY01BWOXI |  |  |
| HLFT7C201AN9ID | HLFT7C201BMOD6 | HTNRPVY01AEU8X |  |  |
| HLFT7C201BXW66 | HLFT7C201BP6O5 | HTNRPVY01ABY79 |  |  |
| HLFT7C201BRHUL | HLFT7C201AA9LA | HTNRPVY01B08XS |  |  |
| HLFT7C201AL4YO | HLFT7C201BIAJ4 | HTNRPVY01BJE4K |  |  |
| HLFT7C201BS8TQ | HLFT7C201AH0K1 | HTNRPVY01A1IYY |  |  |
| HLFT7C201B3PXU | HLFT7C201AMU5W | HTNRPVY01BUE2S |  |  |
| HLFT7C201APRC9 | HLFT7C201B1FHT | HTNRPVY01ANG1Q |  |  |
| HLFT7C201BZXIV | HLFT7C201B3DDF | HTNRPVY01ADA44 |  |  |
| HLFT7C201ATIOK | HLFT7C201BYSHP | HTNRPVY01A6L45 |  |  |
| HLFT7C201BZEM8 | HLFT7C201BOS3T | HTNRPVY01ABMXE |  |  |
| HLFT7C201A1FD8 | HLFT7C201AQWRV | HTNRPVY01BD3CQ |  |  |
| HLFT7C201A61IL | HLFT7C201A70Z1 | HTNRPVY01BKZ6P |  |  |
| HLFT7C201AUBM6 | HLFT7C201ABAEO | HTNRPVY01BB2UT |  |  |
| HLFT7C201ABFSE | HLFT7C201BW62U | HTNRPVY01AUQV6 |  |  |
| HLFT7C201AMB6M | HLFT7C201AEIMC | HTNRPVY01BLGFM |  |  |
| HLFT7C201APNF3 | HLFT7C201AZHHG | HTNRPVY01BTLSL |  |  |
| HLFT7C201AN9D2 | HLFT7C201BJH5Q | HTNRPVY01AZ04S |  |  |
| HLFT7C201BQF9K | HLFT7C201AR099 | HTNRPVY01APASN |  |  |
| HLFT7C201AAKLG | HLFT7C201AVSP3 | HTNRPVY01AA9RB |  |  |
| HLFT7C201BLADE | HLFT7C201ACR1R | HTNRPVY01BJOPB |  |  |
| HLFT7C201ACN14 | HLFT7C201AXGW6 | HTNRPVY01ALSN5 |  |  |
| HLFT7C201ACUNG | HLFT7C201A0PUE | HTNRPVY01AQSP6 |  |  |
| HLFT7C201AZ0W2 | HLFT7C201BVGO3 | HTNRPVY01BW01K |  |  |
| HLFT7C201AR1AC | HLFT7C201AXATR | HTNRPVY01AQSYM |  |  |
| HLFT7C201AJRZ0 | HLFT7C201AV2QX | HTNRPVY01ARFB4 |  |  |
| HLFT7C201BVCV9 | HLFT7C201BWH0W | HTNRPVY01A9ID1 |  |  |
| HLFT7C201BLYQG | HLFT7C201AHBXH | HTNRPVY01A74L2 |  |  |
| HLFT7C201A5TVZ | HLFT7C201AXGNU | HTNRPVY01AU3GF |  |  |
| HLFT7C201BW7NT | HLFT7C201A2ZZX | HTNRPVY01BIWB8 |  |  |
| HLFT7C201AV2OC | HLFT7C201A20DN | HTNRPVY01A67YK |  |  |
| HLFT7C201AM7E3 | HLFT7C201BTVYO | HTNRPVY01BHQRD |  |  |
| HLFT7C201ACKV3 | HLFT7C201BXWN1 | HTNRPVY01ABST7 |  |  |
| HLFT7C201AQDGI | HLFT7C201BUWUG | HTNRPVY01AK0EW |  |  |
| HLFT7C201BL12R | HLFT7C201BU28X | HTNRPVY01A3PHS |  |  |
| HLFT7C201AX0CU | HLFT7C201BL8AR | HTNRPVY01AFOJM |  |  |
| HLFT7C201B0ZI6 | HLFT7C201BTRZ8 | HTNRPVY01AF3GF |  |  |
| HLFT7C201APNRI | HLFT7C201BC9NY | HTNRPVY01BCKJN |  |  |
| HLFT7C201A67HY | HLFT7C201AJOXJ | HTNRPVY01BG769 |  |  |
| HLFT7C201B3MM3 | HLFT7C201ABJRV | HTNRPVY01AFKQ7 |  |  |
| HLFT7C201AIAPD | HLFT7C201BTUXG | HTNRPVY01AHX4B |  |  |
| HLFT7C201AZT5O | HLFT7C201BDGAY | HTNRPVY01BGR13 |  |  |
| HLFT7C201A7QY9 | HLFT7C201AV26U | HTNRPVY01AS9HA |  |  |
| HLFT7C201BYMKU | HLFT7C201A5J7I | HTNRPVY01ARK83 |  |  |
| HLFT7C201AMXTG | HLFT7C201BQY4N | HTNRPVY01A7E0N |  |  |
| HLFT7C201BL1OH | HLFT7C201AD8MZ | HTNRPVY01AMX4N |  |  |
| HLFT7C201BCE43 | HLFT7C201B0NEZ | HTNRPVY01ALYZ7 |  |  |
| HLFT7C201BUWWL | HLFT7C201BFUKQ | HTNRPVY01B2OEZ |  |  |
| HLFT7C201BDDJF | HLFT7C201BAQQ8 | HTNRPVY01BEL16 |  |  |
| HLFT7C201BDP9L | HLFT7C201AO4K6 | HTNRPVY01B268B |  |  |
| HLFT7C201BP25G | HLFT7C201ADDTT | HTNRPVY01AOLOA |  |  |
| HLFT7C201BNMJT | HLFT7C201B05RV | HTNRPVY01AN6UH |  |  |
| HLFT7C201BVGBP | HLFT7C201BH0Q8 | HTNRPVY01ANZUU |  |  |
| HLFT7C201ADGI3 | HLFT7C201BFP7W | HTNRPVY01A89G8 |  |  |
| HLFT7C201AHH9D | HLFT7C201AA2SK | HTNRPVY01A5GX8 |  |  |
| HLFT7C201BH7RY | HLFT7C201AG1UL | HTNRPVY01BECY9 |  |  |
| HLFT7C201BPNHC | HLFT7C201BN2QE | HTNRPVY01APQLL |  |  |
| HLFT7C201BJ340 | HLFT7C201BII9H | HTNRPVY01A9CL8 |  |  |
| HLFT7C201BPAXP | HLFT7C201A3IT3 | HTNRPVY01AZQVG |  |  |
| HLFT7C201AVW4L | HLFT7C201BMO74 | HTNRPVY01ASW9D |  |  |
| HLFT7C201AK26H | HLFT7C201AAJ6C | HTNRPVY01BMHTZ |  |  |
| HLFT7C201AP310 | HLFT7C201ADALU | HTNRPVY01BFNEL |  |  |
| HLFT7C201BAQIT | HLFT7C201AJCFN | HTNRPVY01ACXD5 |  |  |
| HLFT7C201BFATB | HLFT7C201BZ39P | HTNRPVY01AM03C |  |  |
| HLFT7C201BCJ95 | HLFT7C201BOLPL | HTNRPVY01BQM4G |  |  |
| HLFT7C201APH0D | HLFT7C201AROLV | HTNRPVY01BCG6Y |  |  |
| HLFT7C201AUQ86 | HLFT7C201BIWE1 | HTNRPVY01BYZCK |  |  |
| HLFT7C201ACN9K | HLFT7C201AKTLI | HTNRPVY01AD86T |  |  |
| HLFT7C201AQJE3 | HLFT7C201BPWQU | HTNRPVY01A2ANC |  |  |
| HLFT7C201AMUWW | HLFT7C201BDI3T | HTNRPVY01AZKPU |  |  |
| HLFT7C201BKAT9 | HLFT7C201BS844 | HTNRPVY01BP23J |  |  |
| HLFT7C201ADAFF | HLFT7C201BG4RU | HTNRPVY01BI5SQ |  |  |
| HLFT7C201AV5MH | HLFT7C201A8F75 | HTNRPVY01AMEX4 |  |  |
| HLFT7C201AX3B1 | HLFT7C201ABZGD | HTNRPVY01A35P5 |  |  |
| HLFT7C201BAGR6 | HLFT7C201BMK12 | HTNRPVY01BJRLN |  |  |
| HLFT7C201AP6PU | HLFT7C201AS9RF | HTNRPVY01ANJ5L |  |  |
| HLFT7C201AOSRL | HLFT7C201BOO5U | HTNRPVY01AM1DF |  |  |
| HLFT7C201AX5YI | HLFT7C201AF56N | HTNRPVY01AK96K |  |  |
| HLFT7C201BPZ0J | HLFT7C201BEUYE | HTNRPVY01AXNVB |  |  |
| HLFT7C201AD53B | HLFT7C201BDJS9 | HTNRPVY01BNJMD |  |  |
| HLFT7C201BV6E6 | HLFT7C201BCQXA | HTNRPVY01BWISF |  |  |
| HLFT7C201AX6QL | HLFT7C201BKEMT | HTNRPVY01AXREO |  |  |
| HLFT7C201A6U0U | HLFT7C201A9RCK | HTNRPVY01ATC5A |  |  |
| HLFT7C201BTC9L | HLFT7C201A23LM | HTNRPVY01B2J1Y |  |  |
| HLFT7C201BYO47 | HLFT7C201BT4OR | HTNRPVY01B0J5W |  |  |
| HLFT7C201AMK5T | HLFT7C201ASD8W | HTNRPVY01AE05C |  |  |
| HLFT7C201BZEWK | HLFT7C201AFESZ | HTNRPVY01BFKJR |  |  |
| HLFT7C201AJ1DI | HLFT7C201AL839 | HTNRPVY01AHXQJ |  |  |
| HLFT7C201A0PQS | HLFT7C201BIGH1 | HTNRPVY01AKA71 |  |  |
| HLFT7C201AWBWC | HLFT7C201APT9N | HTNRPVY01BCW4G |  |  |
| HLFT7C201AUW1Z | HLFT7C201AZ05H | HTNRPVY01B2KLF |  |  |
| HLFT7C201A6VRD | HLFT7C201BLTN7 | HTNRPVY01BF304 |  |  |
| HLFT7C201A9IZ6 | HLFT7C201AMIBS | HTNRPVY01AE1HX |  |  |
| HLFT7C201BMEAN | HLFT7C201BAMSR | HTNRPVY01BDS0W |  |  |
| HLFT7C201BI8VT | HLFT7C201BG4ZQ | HTNRPVY01AKWQE |  |  |
| HLFT7C201AYR3V | HLFT7C201BL8CO | HTNRPVY01BJ75W |  |  |
| HLFT7C201AV5NJ | HLFT7C201AIGEZ | HTNRPVY01AAD39 |  |  |
| HLFT7C201AL2HB | HLFT7C201BIGKY | HTNRPVY01AL15B |  |  |
| HLFT7C201AVY6K | HLFT7C201B02S5 | HTNRPVY01BJFLY |  |  |
| HLFT7C201AWEWR | HLFT7C201BZ0CK | HTNRPVY01A5G13 |  |  |
| HLFT7C201AT5CZ | HLFT7C201BBFT6 | HTNRPVY01AYMFJ |  |  |
| HLFT7C201A65LI | HLFT7C201A8VPW | HTNRPVY01AXWTB |  |  |
| HLFT7C201AMRD3 | HLFT7C201BOYE1 | HTNRPVY01BNZHV |  |  |
| HLFT7C201A2ZXE | HLFT7C201BPXOB | HTNRPVY01BWRNA |  |  |
| HLFT7C201AK6HM | HLFT7C201BQ5NB | HTNRPVY01BSMZO |  |  |
| HLFT7C201AY8UV | HLFT7C201AQI70 | HTNRPVY01A0MK7 |  |  |
| HLFT7C201A09E7 | HLFT7C201BPTXJ | HTNRPVY01BGVDF |  |  |
| HLFT7C201AMRHS | HLFT7C201BE4UQ | HTNRPVY01AQMFE |  |  |
| HLFT7C201A3LX7 | HLFT7C201BP0KG | HTNRPVY01ALM2J |  |  |
| HLFT7C201ATSN5 | HLFT7C201BUK3P | HTNRPVY01BK3TZ |  |  |
| HLFT7C201BUHQS | HLFT7C201A91U8 | HTNRPVY01BW4I1 |  |  |
| HLFT7C201A0DMZ | HLFT7C201BKALY | HTNRPVY01A3SD7 |  |  |
| HLFT7C201BQWQ1 | HLFT7C201AJIT3 | HTNRPVY01BC0TM |  |  |
| HLFT7C201AMHLN | HLFT7C201BDYZH | HTNRPVY01AVJW6 |  |  |
| HLFT7C201BUKV1 | HLFT7C201BKP7R | HTNRPVY01AZA5C |  |  |
| HLFT7C201AY733 | HLFT7C201A3V4P | HTNRPVY01AP1H5 |  |  |
| HLFT7C201AJ412 | HLFT7C201AGFK7 | HTNRPVY01BC6PV |  |  |
| HLFT7C201BQ2D7 | HLFT7C201AGCKQ | HTNRPVY01BYFR5 |  |  |
| HLFT7C201BUXGF | HLFT7C201BMBU6 | HTNRPVY01AU3VQ |  |  |
| HLFT7C201BAN03 | HLFT7C201AKQKJ | HTNRPVY01BTX9Z |  |  |
| HLFT7C201BP25K | HLFT7C201BBSSP | HTNRPVY01BZ0CC |  |  |
| HLFT7C201AUBB0 | HLFT7C201ATYQF | HTNRPVY01BL8SP |  |  |
| HLFT7C201BCAW8 | HLFT7C201ASZIO | HTNRPVY01AU6ZX |  |  |
| HLFT7C201A6MAP | HLFT7C201A3ZKH | HTNRPVY01AKG0B |  |  |
| HLFT7C201BH3LW | HLFT7C201AKB1Z | HTNRPVY01BGAF3 |  |  |
| HLFT7C201AMBAI | HLFT7C201ATFA7 | HTNRPVY01BKAQO |  |  |
| HLFT7C201BQGEW | HLFT7C201A1ODK | HTNRPVY01AHQ09 |  |  |
| HLFT7C201A61PW | HLFT7C201BMXPT | HTNRPVY01ARLBY |  |  |
| HLFT7C201BCUBT | HLFT7C201AMUCZ | HTNRPVY01AAERC |  |  |
| HLFT7C201BVJWS | HLFT7C201A2M70 | HTNRPVY01A4HUH |  |  |
| HLFT7C201ACKPV | HLFT7C201ATU9J | HTNRPVY01A6RN5 |  |  |
| HLFT7C201BXTS6 | HLFT7C201A29XV | HTNRPVY01BXZ15 |  |  |
| HLFT7C201BQYW3 | HLFT7C201AJVIY | HTNRPVY01A7HCT |  |  |
| HLFT7C201BW1ON | HLFT7C201BN811 | HTNRPVY01BQI3Z |  |  |
| HLFT7C201BXNDY | HLFT7C201A1YB3 | HTNRPVY01BECB3 |  |  |
| HLFT7C201BIJUB | HLFT7C201BC34U | HTNRPVY01AHIGQ |  |  |
| HLFT7C201AUUFY | HLFT7C201B1FAJ | HTNRPVY01BI847 |  |  |
| HLFT7C201B2KUB | HLFT7C201B1YME | HTNRPVY01BTRWY |  |  |
| HLFT7C201BDVWS | HLFT7C201AESU5 | HTNRPVY01BDF99 |  |  |
| HLFT7C201AYISS | HLFT7C201B164R | HTNRPVY01A93ZC |  |  |
| HLFT7C201AQDWF | HLFT7C201A7RG6 | HTNRPVY01AE4V8 |  |  |
| HLFT7C201BKEFH | HLFT7C201BESIV | HTNRPVY01AS6FE |  |  |
| HLFT7C201AZQ87 | HLFT7C201BFT6T | HTNRPVY01AOR0C |  |  |
| HLFT7C201A8SVS | HLFT7C201A2J8P | HTNRPVY01BSQB7 |  |  |
| HLFT7C201BOFXV | HLFT7C201AQ8SD | HTNRPVY01B2AJJ |  |  |
| HLFT7C201AVV49 | HLFT7C201BS8WI | HTNRPVY01AZ1HN |  |  |
| HLFT7C201AW1EX | HLFT7C201AX35J | HTNRPVY01AHU6Z |  |  |
| HLFT7C201AP0X8 | HLFT7C201BH0AH | HTNRPVY01BUDXS |  |  |
| HLFT7C201BMIC7 | HLFT7C201B26SP | HTNRPVY01BAWGG |  |  |
| HLFT7C201APN92 | HLFT7C201BJLBZ | HTNRPVY01A14H3 |  |  |
| HLFT7C201AKZ8Y | HLFT7C201AZRAJ | HTNRPVY01ASA1E |  |  |
| HLFT7C201AF990 | HLFT7C201AZKXB | HTNRPVY01AB107 |  |  |
| HLFT7C201AP6RV | HLFT7C201BXS49 | HTNRPVY01A32IW |  |  |
| HLFT7C201AQZQM | HLFT7C201BYCO3 | HTNRPVY01ATYVW |  |  |
| HLFT7C201AIZ04 | HLFT7C201A05MZ | HTNRPVY01AYMXH |  |  |
| HLFT7C201BV84C | HLFT7C201BQYPI | HTNRPVY01AFZ4Y |  |  |
| HLFT7C201APRDC | HLFT7C201ACOQR | HTNRPVY01ALWIM |  |  |
| HLFT7C201BS58I | HLFT7C201BRBTS | HTNRPVY01AWLN5 |  |  |
| HLFT7C201BSGX5 | HLFT7C201AHEDV | HTNRPVY01AQ8D8 |  |  |
| HLFT7C201A1YI2 | HLFT7C201A0TD1 | HTNRPVY01BOLG8 |  |  |
| HLFT7C201A0AMS | HLFT7C201B0J3V | HTNRPVY01AJI34 |  |  |
| HLFT7C201BX8ZP | HLFT7C201AFXHX | HTNRPVY01ATIK1 |  |  |
| HLFT7C201AE753 | HLFT7C201A6MHI | HTNRPVY01A9LM8 |  |  |
| HLFT7C201BHXZM | HLFT7C201AKXUR | HTNRPVY01BSGZ9 |  |  |
| HLFT7C201A1YG9 | HLFT7C201BHOY3 | HTNRPVY01ARR9B |  |  |
| HLFT7C201BU9TF | HLFT7C201B3GGG | HTNRPVY01AH4JR |  |  |
| HLFT7C201BWRCM | HLFT7C201A64ZX | HTNRPVY01AMNW0 |  |  |
| HLFT7C201BDJJA | HLFT7C201AJB2V | HTNRPVY01BVVZE |  |  |
| HLFT7C201AI6DJ | HLFT7C201A6X0B | HTNRPVY01BLPF2 |  |  |
| HLFT7C201AUHYB | HLFT7C201AH1FQ | HTNRPVY01AVY8M |  |  |
| HLFT7C201B1N6J | HLFT7C201AH4AG | HTNRPVY01AAD07 |  |  |
| HLFT7C201BMUQF | HLFT7C201BC0G5 | HTNRPVY01BRXOL |  |  |
| HLFT7C201BMVEF | HLFT7C201BNZL5 | HTNRPVY01AVVVI |  |  |
| HLFT7C201AMBSP | HLFT7C201BTGIX | HTNRPVY01A09KU |  |  |
| HLFT7C201AYGC7 | HLFT7C201BQMKT | HTNRPVY01AEB77 |  |  |
| HLFT7C201BJSFG | HLFT7C201A964R | HTNRPVY01BBPX9 |  |  |
| HLFT7C201AIM3T | HLFT7C201A4H7Q | HTNRPVY01AI5RD |  |  |
| HLFT7C201AU9ZL | HLFT7C201AHOBX | HTNRPVY01AANBA |  |  |
| HLFT7C201BBRWH | HLFT7C201AUHQF | HTNRPVY01BQTOW |  |  |
| HLFT7C201BL5DY | HLFT7C201BLPKP | HTNRPVY01A9VB7 |  |  |
| HLFT7C201BZXCS | HLFT7C201B1BI3 | HTNRPVY01BBLR3 |  |  |
| HLFT7C201AB445 | HLFT7C201BWRG8 | HTNRPVY01BSZA1 |  |  |
| HLFT7C201BBSGU | HLFT7C201AN9KL | HTNRPVY01AA0ND |  |  |
| HLFT7C201A2TR8 | HLFT7C201BQY8V | HTNRPVY01BEE9B |  |  |
| HLFT7C201ABS2C | HLFT7C201BMQ0W | HTNRPVY01ASS84 |  |  |
| HLFT7C201A6O7J | HLFT7C201A7Z36 | HTNRPVY01BIW84 |  |  |
| HLFT7C201BG5H4 | HLFT7C201BSTI5 | HTNRPVY01BEUK7 |  |  |
| HLFT7C201ALPUB | HLFT7C201BMLZF | HTNRPVY01BIDIG |  |  |
| HLFT7C201AD8KJ | HLFT7C201BSW99 | HTNRPVY01ANZZ4 |  |  |
| HLFT7C201AUHQB | HLFT7C201AYV98 | HTNRPVY01ANQ4Z |  |  |
| HLFT7C201AQS6N | HLFT7C201AZ4BB | HTNRPVY01APKAI |  |  |
| HLFT7C201B3R6Y | HLFT7C201ANTYA | HTNRPVY01ALGWT |  |  |
| HLFT7C201ADZ0E | HLFT7C201AKN8E | HTNRPVY01BGJOD |  |  |
| HLFT7C201B1FDB | HLFT7C201AU7MQ | HTNRPVY01B3YX6 |  |  |
| HLFT7C201BF62D | HLFT7C201AQ2CN | HTNRPVY01BE4PD |  |  |
| HLFT7C201AHOFL | HLFT7C201AG5K6 | HTNRPVY01A5DYQ |  |  |
| HLFT7C201BN2HO | HLFT7C201BN3AW | HTNRPVY01BEBI0 |  |  |
| HLFT7C201BBWC1 | HLFT7C201B0MDF | HTNRPVY01BKXDF |  |  |
| HLFT7C201BT11E | HLFT7C201AK0WE | HTNRPVY01AUB6L |  |  |
| HLFT7C201AK3I3 | HLFT7C201BOIW3 | HTNRPVY01BIMQF |  |  |
| HLFT7C201BADPS | HLFT7C201BT4FV | HTNRPVY01AEII6 |  |  |
| HLFT7C201BBJLZ | HLFT7C201BQC1H | HTNRPVY01AMT9P |  |  |
| HLFT7C201AVQAT | HLFT7C201BK9CU | HTNRPVY01BBSK5 |  |  |
| HLFT7C201AC08B | HLFT7C201AYFOY | HTNRPVY01A8AJD |  |  |
| HLFT7C201APEQJ | HLFT7C201BHIKY | HTNRPVY01BNG4D |  |  |
| HLFT7C201BSBBO | HLFT7C201ABOY6 | HTNRPVY01AVC7W |  |  |
| HLFT7C201A946Z | HLFT7C201BIMVK | HTNRPVY01BA3N7 |  |  |
| HLFT7C201BU975 | HLFT7C201A6RPT | HTNRPVY01BGZCC |  |  |
| HLFT7C201BYU7R | HLFT7C201BMHL0 | HTNRPVY01A7WYQ |  |  |
| HLFT7C201BHN30 | HLFT7C201AR1Y2 | HTNRPVY01BKZ2H |  |  |
| HLFT7C201BO7M3 | HLFT7C201AGJO4 | HTNRPVY01BR6Y6 |  |  |
| HLFT7C201AFURK | HLFT7C201BKK46 | HTNRPVY01BBL81 |  |  |
| HLFT7C201ACROZ | HLFT7C201AF3HY | HTNRPVY01AKQ1M |  |  |
| HLFT7C201AXGNN | HLFT7C201BX8X5 | HTNRPVY01A5NTA |  |  |
| HLFT7C201BJVHD | HLFT7C201B3GEL | HTNRPVY01AVMHE |  |  |
| HLFT7C201B0TL4 | HLFT7C201BB709 | HTNRPVY01BUEZ3 |  |  |
