# Supplementary material for: Gene Discovery through Transcriptome Sequencing for the Invasive Mussel Limnoperna fortunei
Source: PLoS One. 2014 Jul 21;9(7):e102973. doi: 10.1371/journal.pone.0102973 (PMC4105566; doi:10.1371/journal.pone.0102973)
Supplement: Table S3 — BLAST result for full-length search: C. gigas IDs together with the unigenes of L. fortunei annotated for each C. gigas sequence. (DOCX) [file pone.0102973.s003.docx]

**SUPPORTING INFORMATION S3**

Uliano-Silva *et al.,* 2014. Gene discovery through transcriptome sequencing for the invasive mussel *Limnoperna fortunei.*

BLAST result for full-length search: *C. gigas* IDs together with the unigenes of *L. fortunei* annotated for each *C. gigas* sequences.

The unigenes of *L. fortunei* are available for download at: **http://goo.gl/mNYPbX**.

CGI_10010646 634 (1, 634) 634 100.0

Contig1244

Contig1903

Contig2015

Contig2015

Contig2015

Contig2080

Contig2080

Contig2185

Contig2232

Contig2985

HLFT7C201ALAUC

HLFT7C201BNJOD

HLFT7C201AC6LC

HLFT7C201AC6LC

HLFT7C201A9U2N

HLFT7C201BO77D

HLFT7C201BO77D

HLFT7C201BW31X

HLFT7C201BW31X

HLFT7C201A9US4

HLFT7C201A9E27

HLFT7C201AIM3T

HLFT7C201AIM3T

HLFT7C201AM056

HLFT7C201A4BD5

HLFT7C201BPKPW

HLFT7C201BCXKK

HLFT7C201BR1AD

HLFT7C201BR1AD

HLFT7C201BL1TF

HLFT7C201AXJ5F

HLFT7C201AXJ5F

HLFT7C201AXJ5F

HLFT7C201BD8RK

HLFT7C201BD8RK

HLFT7C201BD8RK

HLFT7C201A8M03

HLFT7C201A94LK

HLFT7C201BBCNU

HLFT7C201BEXWM

HLFT7C201AS23T

HLFT7C201AS23T

HLFT7C201A1BQY

HLFT7C201A1BQY

HLFT7C201AKUKH

HLFT7C201BJF4X

HLFT7C201AQQFK

HLFT7C201BTJOF

HLFT7C201BTJOF

HLFT7C201BNKOL

HLFT7C201ALAIF

HLFT7C201AS0UQ

HTNRPVY01BSNWM

HTNRPVY01AQ8ZK

HTNRPVY01BPZYR

HTNRPVY01BPZYR

HTNRPVY01BKGYL

HTNRPVY01ADGI4

HTNRPVY01A59B2

HTNRPVY01A0GY4

HTNRPVY01BZN77

HTNRPVY01BZ953

HTNRPVY01BZ953

HTNRPVY01A8YOK

HTNRPVY01BA64J

HTNRPVY01BBAC1

HTNRPVY01ANTES

HTNRPVY01ATU4J

HTNRPVY01ATJMJ

HTNRPVY01AUBCY

HTNRPVY01BAWOK

HTNRPVY01BK292

HTNRPVY01A8WR7

HTNRPVY01AOU00

HTNRPVY01AA9V8

HTNRPVY01AEUW9

HTNRPVY01A47R4

HTNRPVY01BSKPM

HTNRPVY01AXDO6

HTNRPVY01BTSRJ

HTNRPVY01BTSRJ

HTNRPVY01AHLGO

HTNRPVY01AKXWT

HTNRPVY01ADG5B

HTNRPVY01ALSXR

HTNRPVY01ALSXR

HTNRPVY01AGCE7

HTNRPVY01AK29Y

HTNRPVY01A6R99

HTNRPVY01AIEFU

HTNRPVY01AIEFU

HTNRPVY01ASQ82

HTNRPVY01ASQ82

HTNRPVY01AFM5Y

HTNRPVY01A02Z0

HTNRPVY01A02Z0

HTNRPVY01A3JCQ

HTNRPVY01AFKRL

HTNRPVY01AMRH3

HTNRPVY01BA6F3

HTNRPVY01BA6F3

HTNRPVY01BBSMU

HTNRPVY01AL50U

HTNRPVY01AHX4W

HTNRPVY01BTB95

HTNRPVY01BTB95

HTNRPVY01BTB95

HTNRPVY01BROLO

HTNRPVY01BVPYF

HTNRPVY01BVPYF

CGI_10010647 634 (1, 634) 634 100.0

Contig1244

Contig1903

Contig2015

Contig2015

Contig2015

Contig2080

Contig2080

Contig2185

Contig2232

Contig2985

HLFT7C201ALAUC

HLFT7C201BNJOD

HLFT7C201AC6LC

HLFT7C201AC6LC

HLFT7C201A9U2N

HLFT7C201BO77D

HLFT7C201BO77D

HLFT7C201BW31X

HLFT7C201BW31X

HLFT7C201A9US4

HLFT7C201A9E27

HLFT7C201AIM3T

HLFT7C201AIM3T

HLFT7C201AM056

HLFT7C201A4BD5

HLFT7C201BPKPW

HLFT7C201BCXKK

HLFT7C201BR1AD

HLFT7C201BR1AD

HLFT7C201BL1TF

HLFT7C201AXJ5F

HLFT7C201AXJ5F

HLFT7C201AXJ5F

HLFT7C201BD8RK

HLFT7C201BD8RK

HLFT7C201BD8RK

HLFT7C201A8M03

HLFT7C201A94LK

HLFT7C201BBCNU

HLFT7C201BEXWM

HLFT7C201AS23T

HLFT7C201AS23T

HLFT7C201A1BQY

HLFT7C201A1BQY

HLFT7C201AKUKH

HLFT7C201BJF4X

HLFT7C201AQQFK

HLFT7C201BTJOF

HLFT7C201BTJOF

HLFT7C201BNKOL

HLFT7C201ALAIF

HLFT7C201AS0UQ

HTNRPVY01BSNWM

HTNRPVY01AQ8ZK

HTNRPVY01BPZYR

HTNRPVY01BPZYR

HTNRPVY01BKGYL

HTNRPVY01ADGI4

HTNRPVY01A59B2

HTNRPVY01A0GY4

HTNRPVY01BZN77

HTNRPVY01BZ953

HTNRPVY01BZ953

HTNRPVY01A8YOK

HTNRPVY01BA64J

HTNRPVY01BBAC1

HTNRPVY01ANTES

HTNRPVY01ATU4J

HTNRPVY01ATJMJ

HTNRPVY01AUBCY

HTNRPVY01BAWOK

HTNRPVY01BK292

HTNRPVY01A8WR7

HTNRPVY01AOU00

HTNRPVY01AA9V8

HTNRPVY01AEUW9

HTNRPVY01A47R4

HTNRPVY01BSKPM

HTNRPVY01AXDO6

HTNRPVY01BTSRJ

HTNRPVY01BTSRJ

HTNRPVY01AHLGO

HTNRPVY01AKXWT

HTNRPVY01ADG5B

HTNRPVY01ALSXR

HTNRPVY01ALSXR

HTNRPVY01AGCE7

HTNRPVY01AK29Y

HTNRPVY01A6R99

HTNRPVY01AIEFU

HTNRPVY01AIEFU

HTNRPVY01ASQ82

HTNRPVY01ASQ82

HTNRPVY01AFM5Y

HTNRPVY01A02Z0

HTNRPVY01A02Z0

HTNRPVY01A3JCQ

HTNRPVY01AFKRL

HTNRPVY01AMRH3

HTNRPVY01BA6F3

HTNRPVY01BA6F3

HTNRPVY01BBSMU

HTNRPVY01AL50U

HTNRPVY01AHX4W

HTNRPVY01BTB95

HTNRPVY01BTB95

HTNRPVY01BTB95

HTNRPVY01BROLO

HTNRPVY01BVPYF

HTNRPVY01BVPYF

CGI_10003417 634 (1, 634) 634 100.0

Contig1244

Contig1903

Contig2015

Contig2015

Contig2015

Contig2080

Contig2080

Contig2185

Contig2232

Contig2985

HLFT7C201ALAUC

HLFT7C201BNJOD

HLFT7C201AC6LC

HLFT7C201AC6LC

HLFT7C201A9U2N

HLFT7C201BO77D

HLFT7C201BO77D

HLFT7C201BW31X

HLFT7C201BW31X

HLFT7C201A9US4

HLFT7C201A9E27

HLFT7C201AIM3T

HLFT7C201AIM3T

HLFT7C201AM056

HLFT7C201A4BD5

HLFT7C201BPKPW

HLFT7C201BCXKK

HLFT7C201BR1AD

HLFT7C201BR1AD

HLFT7C201BL1TF

HLFT7C201AXJ5F

HLFT7C201AXJ5F

HLFT7C201AXJ5F

HLFT7C201BD8RK

HLFT7C201BD8RK

HLFT7C201A8M03

HLFT7C201A94LK

HLFT7C201BBCNU

HLFT7C201BEXWM

HLFT7C201AS23T

HLFT7C201AS23T

HLFT7C201AS23T

HLFT7C201A1BQY

HLFT7C201A1BQY

HLFT7C201AKUKH

HLFT7C201BJF4X

HLFT7C201AQQFK

HLFT7C201BTJOF

HLFT7C201BNKOL

HLFT7C201ALAIF

HLFT7C201AS0UQ

HTNRPVY01BSNWM

HTNRPVY01BPZYR

HTNRPVY01BPZYR

HTNRPVY01BKGYL

HTNRPVY01ADGI4

HTNRPVY01A59B2

HTNRPVY01A0GY4

HTNRPVY01BZN77

HTNRPVY01BZ953

HTNRPVY01BZ953

HTNRPVY01A8YOK

HTNRPVY01BA64J

HTNRPVY01BBAC1

HTNRPVY01ANTES

HTNRPVY01ATU4J

HTNRPVY01ATJMJ

HTNRPVY01AUBCY

HTNRPVY01BAWOK

HTNRPVY01BK292

HTNRPVY01A8WR7

HTNRPVY01AOU00

HTNRPVY01AA9V8

HTNRPVY01AEUW9

HTNRPVY01A47R4

HTNRPVY01BSKPM

HTNRPVY01AXDO6

HTNRPVY01BTSRJ

HTNRPVY01BTSRJ

HTNRPVY01AHLGO

HTNRPVY01AKXWT

HTNRPVY01ADG5B

HTNRPVY01ALSXR

HTNRPVY01ALSXR

HTNRPVY01AGCE7

HTNRPVY01AK29Y

HTNRPVY01A6R99

HTNRPVY01AIEFU

HTNRPVY01AIEFU

HTNRPVY01ASQ82

HTNRPVY01ASQ82

HTNRPVY01AFM5Y

HTNRPVY01A02Z0

HTNRPVY01A02Z0

HTNRPVY01A3JCQ

HTNRPVY01AFKRL

HTNRPVY01AMRH3

HTNRPVY01BA6F3

HTNRPVY01BA6F3

HTNRPVY01BBSMU

HTNRPVY01AL50U

HTNRPVY01AHX4W

HTNRPVY01BTB95

HTNRPVY01BTB95

HTNRPVY01BTB95

HTNRPVY01BROLO

HTNRPVY01BVPYF

HTNRPVY01BVPYF

CGI_10002594 634 (1, 634) 634 100.0

Contig1244

Contig1903

Contig2015

Contig2015

Contig2015

Contig2080

Contig2080

Contig2185

Contig2232

Contig2985

HLFT7C201ALAUC

HLFT7C201BNJOD

HLFT7C201AC6LC

HLFT7C201AC6LC

HLFT7C201A9U2N

HLFT7C201BO77D

HLFT7C201BO77D

HLFT7C201BW31X

HLFT7C201BW31X

HLFT7C201A9US4

HLFT7C201A9E27

HLFT7C201AIM3T

HLFT7C201AIM3T

HLFT7C201AM056

HLFT7C201A4BD5

HLFT7C201BPKPW

HLFT7C201BCXKK

HLFT7C201BR1AD

HLFT7C201BR1AD

HLFT7C201BL1TF

HLFT7C201AXJ5F

HLFT7C201AXJ5F

HLFT7C201AXJ5F

HLFT7C201BD8RK

HLFT7C201BD8RK

HLFT7C201BD8RK

HLFT7C201A8M03

HLFT7C201A94LK

HLFT7C201BBCNU

HLFT7C201BEXWM

HLFT7C201AS23T

HLFT7C201AS23T

HLFT7C201A1BQY

HLFT7C201A1BQY

HLFT7C201AKUKH

HLFT7C201BJF4X

HLFT7C201AQQFK

HLFT7C201BTJOF

HLFT7C201BTJOF

HLFT7C201BNKOL

HLFT7C201ALAIF

HLFT7C201AS0UQ

HTNRPVY01BSNWM

HTNRPVY01AQ8ZK

HTNRPVY01BPZYR

HTNRPVY01BPZYR

HTNRPVY01BKGYL

HTNRPVY01ADGI4

HTNRPVY01A59B2

HTNRPVY01A0GY4

HTNRPVY01BZN77

HTNRPVY01BZ953

HTNRPVY01BZ953

HTNRPVY01A8YOK

HTNRPVY01BA64J

HTNRPVY01BBAC1

HTNRPVY01ANTES

HTNRPVY01ATU4J

HTNRPVY01ATJMJ

HTNRPVY01AUBCY

HTNRPVY01BAWOK

HTNRPVY01BK292

HTNRPVY01A8WR7

HTNRPVY01AOU00

HTNRPVY01AA9V8

HTNRPVY01AEUW9

HTNRPVY01A47R4

HTNRPVY01BSKPM

HTNRPVY01AXDO6

HTNRPVY01BTSRJ

HTNRPVY01BTSRJ

HTNRPVY01AHLGO

HTNRPVY01AKXWT

HTNRPVY01ADG5B

HTNRPVY01ALSXR

HTNRPVY01ALSXR

HTNRPVY01AGCE7

HTNRPVY01AK29Y

HTNRPVY01A6R99

HTNRPVY01AIEFU

HTNRPVY01AIEFU

HTNRPVY01ASQ82

HTNRPVY01ASQ82

HTNRPVY01AFM5Y

HTNRPVY01A02Z0

HTNRPVY01A02Z0

HTNRPVY01A3JCQ

HTNRPVY01AFKRL

HTNRPVY01AMRH3

HTNRPVY01BA6F3

HTNRPVY01BA6F3

HTNRPVY01BBSMU

HTNRPVY01AL50U

HTNRPVY01AHX4W

HTNRPVY01BTB95

HTNRPVY01BTB95

HTNRPVY01BTB95

HTNRPVY01BROLO

HTNRPVY01BVPYF

HTNRPVY01BVPYF

CGI_10028167 593 (1, 582) 582 98.1450252951

Contig1244

Contig1903

Contig2015

Contig2015

Contig2080

Contig2080

Contig2232

Contig2985

HLFT7C201ALAUC

HLFT7C201AC6LC

HLFT7C201A9U2N

HLFT7C201BO77D

HLFT7C201A9US4

HLFT7C201A9E27

HLFT7C201AIM3T

HLFT7C201AM056

HLFT7C201A4BD5

HLFT7C201BPKPW

HLFT7C201BCXKK

HLFT7C201BR1AD

HLFT7C201BR1AD

HLFT7C201AXJ5F

HLFT7C201AXJ5F

HLFT7C201AXJ5F

HLFT7C201A8M03

HLFT7C201A94LK

HLFT7C201BBCNU

HLFT7C201BEXWM

HLFT7C201AS23T

HLFT7C201AS23T

HLFT7C201AS23T

HLFT7C201A1BQY

HLFT7C201AKUKH

HLFT7C201AQQFK

HLFT7C201ALAIF

HTNRPVY01BPZYR

HTNRPVY01BPZYR

HTNRPVY01BKGYL

HTNRPVY01A59B2

HTNRPVY01A0GY4

HTNRPVY01BZN77

HTNRPVY01BZ953

HTNRPVY01A8YOK

HTNRPVY01BA64J

HTNRPVY01BBAC1

HTNRPVY01ANTES

HTNRPVY01ANTES

HTNRPVY01ATU4J

HTNRPVY01AUBCY

HTNRPVY01BK292

HTNRPVY01BK292

HTNRPVY01A8WR7

HTNRPVY01AEUW9

HTNRPVY01AXDO6

HTNRPVY01BTSRJ

HTNRPVY01BTSRJ

HTNRPVY01AKXWT

HTNRPVY01ALSXR

HTNRPVY01ALSXR

HTNRPVY01AGCE7

HTNRPVY01AK29Y

HTNRPVY01A6R99

HTNRPVY01AIEFU

HTNRPVY01AIEFU

HTNRPVY01ASQ82

HTNRPVY01AFM5Y

HTNRPVY01A02Z0

HTNRPVY01A3JCQ

HTNRPVY01AFKRL

HTNRPVY01AL50U

HTNRPVY01BTB95

HTNRPVY01BVPYF

CGI_10018425 468 (9, 457) 449 95.9401709402

Contig1244

Contig1903

Contig2015

Contig2080

Contig2232

Contig2985

HLFT7C201ALAUC

HLFT7C201BNJOD

HLFT7C201A9U2N

HLFT7C201BO77D

HLFT7C201A9E27

HLFT7C201AIM3T

HLFT7C201AM056

HLFT7C201A4BD5

HLFT7C201BPKPW

HLFT7C201BR1AD

HLFT7C201AXJ5F

HLFT7C201AXJ5F

HLFT7C201AXJ5F

HLFT7C201A8M03

HLFT7C201BEXWM

HLFT7C201AS23T

HLFT7C201AS23T

HLFT7C201AS23T

HLFT7C201A1BQY

HLFT7C201AKUKH

HLFT7C201AQQFK

HTNRPVY01BPZYR

HTNRPVY01BPZYR

HTNRPVY01BKGYL

HTNRPVY01ADGI4

HTNRPVY01A0GY4

HTNRPVY01BZ953

HTNRPVY01BZ953

HTNRPVY01A8YOK

HTNRPVY01BBAC1

HTNRPVY01BBAC1

HTNRPVY01ANTES

HTNRPVY01ATU4J

HTNRPVY01AUBCY

HTNRPVY01BK292

HTNRPVY01A8WR7

HTNRPVY01AA9V8

HTNRPVY01AEUW9

HTNRPVY01A47R4

HTNRPVY01BSKPM

HTNRPVY01AXDO6

HTNRPVY01BTSRJ

HTNRPVY01BTSRJ

HTNRPVY01AHLGO

HTNRPVY01AKXWT

HTNRPVY01ADG5B

HTNRPVY01AGCE7

HTNRPVY01AK29Y

HTNRPVY01A6R99

HTNRPVY01AIEFU

HTNRPVY01AIEFU

HTNRPVY01ASQ82

HTNRPVY01AFM5Y

HTNRPVY01A02Z0

HTNRPVY01A3JCQ

HTNRPVY01BBSMU

HTNRPVY01AL50U

CGI_10028173 633 (7, 618) 612 96.682464455

Contig1244

Contig1903

Contig2015

Contig2015

Contig2080

Contig2080

Contig2232

Contig2985

HLFT7C201ALAUC

HLFT7C201AC6LC

HLFT7C201AC6LC

HLFT7C201A9U2N

HLFT7C201BO77D

HLFT7C201A9US4

HLFT7C201A9E27

HLFT7C201AIM3T

HLFT7C201AM056

HLFT7C201A4BD5

HLFT7C201A4BD5

HLFT7C201BPKPW

HLFT7C201BCXKK

HLFT7C201BR1AD

HLFT7C201BR1AD

HLFT7C201AXJ5F

HLFT7C201AXJ5F

HLFT7C201AXJ5F

HLFT7C201BD8RK

HLFT7C201A8M03

HLFT7C201A94LK

HLFT7C201BBCNU

HLFT7C201BEXWM

HLFT7C201AS23T

HLFT7C201AS23T

HLFT7C201AS23T

HLFT7C201A1BQY

HLFT7C201AKUKH

HLFT7C201AQQFK

HLFT7C201BTJOF

HLFT7C201ALAIF

HTNRPVY01BPZYR

HTNRPVY01BPZYR

HTNRPVY01BKGYL

HTNRPVY01A59B2

HTNRPVY01A0GY4

HTNRPVY01BZN77

HTNRPVY01BZ953

HTNRPVY01BZ953

HTNRPVY01A8YOK

HTNRPVY01BA64J

HTNRPVY01BBAC1

HTNRPVY01ANTES

HTNRPVY01ATU4J

HTNRPVY01AUBCY

HTNRPVY01BK292

HTNRPVY01BK292

HTNRPVY01A8WR7

HTNRPVY01AA9V8

HTNRPVY01AEUW9

HTNRPVY01A47R4

HTNRPVY01AXDO6

HTNRPVY01BTSRJ

HTNRPVY01BTSRJ

HTNRPVY01AHLGO

HTNRPVY01AKXWT

HTNRPVY01ADG5B

HTNRPVY01ALSXR

HTNRPVY01AGCE7

HTNRPVY01AK29Y

HTNRPVY01A6R99

HTNRPVY01AIEFU

HTNRPVY01AIEFU

HTNRPVY01ASQ82

HTNRPVY01AFM5Y

HTNRPVY01A02Z0

HTNRPVY01A3JCQ

HTNRPVY01AFKRL

HTNRPVY01AL50U

HTNRPVY01BVPYF

CGI_10015492 661 (3, 655) 653 98.7897125567

Contig1244

Contig1903

Contig2015

Contig2015

Contig2015

Contig2080

Contig2080

Contig2185

Contig2232

Contig2232

Contig2985

HLFT7C201ALAUC

HLFT7C201BNJOD

HLFT7C201AC6LC

HLFT7C201AC6LC

HLFT7C201A9U2N

HLFT7C201BO77D

HLFT7C201BO77D

HLFT7C201BW31X

HLFT7C201BW31X

HLFT7C201A9US4

HLFT7C201A9E27

HLFT7C201AIM3T

HLFT7C201AIM3T

HLFT7C201AM056

HLFT7C201A4BD5

HLFT7C201BPKPW

HLFT7C201BCXKK

HLFT7C201BCXKK

HLFT7C201BR1AD

HLFT7C201BR1AD

HLFT7C201BL1TF

HLFT7C201AXJ5F

HLFT7C201AXJ5F

HLFT7C201AXJ5F

HLFT7C201BD8RK

HLFT7C201BD8RK

HLFT7C201A8M03

HLFT7C201A94LK

HLFT7C201BBCNU

HLFT7C201BEXWM

HLFT7C201AS23T

HLFT7C201AS23T

HLFT7C201AS23T

HLFT7C201A1BQY

HLFT7C201AKUKH

HLFT7C201BJF4X

HLFT7C201AQQFK

HLFT7C201BTJOF

HLFT7C201ALAIF

HLFT7C201AS0UQ

HLFT7C201AS0UQ

HTNRPVY01BSNWM

HTNRPVY01BPZYR

HTNRPVY01BPZYR

HTNRPVY01BKGYL

HTNRPVY01ADGI4

HTNRPVY01A59B2

HTNRPVY01A59B2

HTNRPVY01A0GY4

HTNRPVY01A0GY4

HTNRPVY01BZN77

HTNRPVY01BZ953

HTNRPVY01BZ953

HTNRPVY01A8YOK

HTNRPVY01BA64J

HTNRPVY01BBAC1

HTNRPVY01BBAC1

HTNRPVY01ANTES

HTNRPVY01ATU4J

HTNRPVY01AUBCY

HTNRPVY01BAWOK

HTNRPVY01BK292

HTNRPVY01A8WR7

HTNRPVY01AOU00

HTNRPVY01AA9V8

HTNRPVY01AEUW9

HTNRPVY01A47R4

HTNRPVY01BSKPM

HTNRPVY01AXDO6

HTNRPVY01BTSRJ

HTNRPVY01BTSRJ

HTNRPVY01AHLGO

HTNRPVY01AKXWT

HTNRPVY01ADG5B

HTNRPVY01ALSXR

HTNRPVY01ALSXR

HTNRPVY01AGCE7

HTNRPVY01AK29Y

HTNRPVY01A6R99

HTNRPVY01AIEFU

HTNRPVY01AIEFU

HTNRPVY01ASQ82

HTNRPVY01ASQ82

HTNRPVY01AFM5Y

HTNRPVY01A02Z0

HTNRPVY01A3JCQ

HTNRPVY01AFKRL

HTNRPVY01AFKRL

HTNRPVY01BA6F3

HTNRPVY01BA6F3

HTNRPVY01BBSMU

HTNRPVY01AL50U

HTNRPVY01BTB95

HTNRPVY01BTB95

HTNRPVY01BTB95

HTNRPVY01BVPYF

CGI_10023367 445 (2, 423) 422 94.8314606742

Contig1244

Contig2080

Contig2232

Contig2985

HLFT7C201BO77D

HLFT7C201A9E27

HLFT7C201AIM3T

HLFT7C201AM056

HLFT7C201BPKPW

HLFT7C201AXJ5F

HLFT7C201AXJ5F

HLFT7C201A8M03

HLFT7C201BEXWM

HLFT7C201AS23T

HLFT7C201A1BQY

HLFT7C201AQQFK

HTNRPVY01BKGYL

HTNRPVY01A0GY4

HTNRPVY01BZ953

HTNRPVY01BZ953

HTNRPVY01BBAC1

HTNRPVY01ATU4J

HTNRPVY01A8WR7

HTNRPVY01AEUW9

HTNRPVY01BTSRJ

HTNRPVY01BTSRJ

HTNRPVY01AKXWT

HTNRPVY01AKXWT

HTNRPVY01AK29Y

HTNRPVY01AIEFU

HTNRPVY01A3JCQ

HTNRPVY01AL50U

HTNRPVY01AL50U

HTNRPVY01BVPYF

CGI_10002823 615 (4, 613) 610 99.1869918699

Contig1244

Contig1903

Contig2015

Contig2080

Contig2080

Contig2232

Contig2985

HLFT7C201ALAUC

HLFT7C201AC6LC

HLFT7C201AC6LC

HLFT7C201A9U2N

HLFT7C201BO77D

HLFT7C201BO77D

HLFT7C201BW31X

HLFT7C201A9US4

HLFT7C201A9E27

HLFT7C201A9E27

HLFT7C201AIM3T

HLFT7C201AM056

HLFT7C201BPKPW

HLFT7C201BCXKK

HLFT7C201BR1AD

HLFT7C201BL1TF

HLFT7C201AXJ5F

HLFT7C201AXJ5F

HLFT7C201AXJ5F

HLFT7C201BD8RK

HLFT7C201BD8RK

HLFT7C201A8M03

HLFT7C201A94LK

HLFT7C201BBCNU

HLFT7C201BEXWM

HLFT7C201AS23T

HLFT7C201AS23T

HLFT7C201AS23T

HLFT7C201A1BQY

HLFT7C201AKUKH

HLFT7C201BJF4X

HLFT7C201AQQFK

HLFT7C201BTJOF

HLFT7C201BTJOF

HLFT7C201BTJOF

HLFT7C201ALAIF

HLFT7C201AS0UQ

HLFT7C201AS0UQ

HTNRPVY01BPZYR

HTNRPVY01BPZYR

HTNRPVY01BKGYL

HTNRPVY01ADGI4

HTNRPVY01A59B2

HTNRPVY01A0GY4

HTNRPVY01BZN77

HTNRPVY01BZ953

HTNRPVY01BZ953

HTNRPVY01A8YOK

HTNRPVY01BA64J

HTNRPVY01BBAC1

HTNRPVY01ANTES

HTNRPVY01ANTES

HTNRPVY01ATU4J

HTNRPVY01ATU4J

HTNRPVY01AUBCY

HTNRPVY01BAWOK

HTNRPVY01BK292

HTNRPVY01BK292

HTNRPVY01A8WR7

HTNRPVY01AA9V8

HTNRPVY01AEUW9

HTNRPVY01A47R4

HTNRPVY01BSKPM

HTNRPVY01AXDO6

HTNRPVY01BTSRJ

HTNRPVY01BTSRJ

HTNRPVY01AHLGO

HTNRPVY01AKXWT

HTNRPVY01ADG5B

HTNRPVY01ALSXR

HTNRPVY01ALSXR

HTNRPVY01AK29Y

HTNRPVY01A6R99

HTNRPVY01A6R99

HTNRPVY01AIEFU

HTNRPVY01AIEFU

HTNRPVY01A02Z0

HTNRPVY01A3JCQ

HTNRPVY01AFKRL

HTNRPVY01BA6F3

HTNRPVY01BA6F3

HTNRPVY01BBSMU

HTNRPVY01AL50U

HTNRPVY01BTB95

HTNRPVY01BTB95

HTNRPVY01BVPYF
