# Supplementary material for: Gene Discovery through Transcriptome Sequencing for the Invasive Mussel Limnoperna fortunei
Source: PLoS One. 2014 Jul 21;9(7):e102973. doi: 10.1371/journal.pone.0102973 (PMC4105566; doi:10.1371/journal.pone.0102973)
Supplement: Table S5 — IDs of unigenes annotated against C. gigas sequences related to cellular defenses. (DOCX) [file pone.0102973.s005.docx]

**SUPPORTING INFORMATION S5**

Uliano-Silva *et al.,* 2014. Gene discovery through transcriptome sequencing for the invasive mussel *Limnoperna fortunei.*

IDs of unigenes related to defense annotated by BLAST against *C. gigas* sequences. The unigenes of *L. fortunei* are available for download at: **http://goo.gl/mNYPbX** .

**CAT**

HLFT7C201AV5QG
HLFT7C201BN5X7
HTNRPVY01A4EW3
HTNRPVY01A9E4H

**SOD**

Contig514
HLFT7C201A6R2K
HLFT7C201AMVID
HLFT7C201ARCT6
HLFT7C201BFUKQ
HLFT7C201BIDJH
HLFT7C201BK6SS
HLFT7C201BMUMQ
HLFT7C201BQYSU
HLFT7C201BT5NT
HLFT7C201BV5QP
HTNRPVY01A1CC7
HTNRPVY01AA9NE
HTNRPVY01AHCHF
HTNRPVY01AJCE1
HTNRPVY01AMXM1
HTNRPVY01AVJSJ
HTNRPVY01BL8CF
HTNRPVY01BLJBA
HTNRPVY01BQSHE

**GR**

Contig3028
HLFT7C201A4934
HLFT7C201AWVV1
HLFT7C201AY1LE
HLFT7C201B1E9X
HLFT7C201BFZXX
HTNRPVY01A6PIK
HTNRPVY01A7X5A
HTNRPVY01ASKIR
HTNRPVY01BIXB3

**GPX**

Contig4330
HLFT7C201AJ4SX
HLFT7C201BDQC9
HLFT7C201BOB5U
HTNRPVY01A77QJ
HTNRPVY01AFW9O
HTNRPVY01AG8YJ
HTNRPVY01AOI37
HTNRPVY01BBVN6
HTNRPVY01BIPRT
HTNRPVY01BJMDS
HTNRPVY01BP62F
HTNRPVY01BSD6L

**PRX**

HLFT7C201APXEH
HLFT7C201AYPTW
HLFT7C201BIVZC
HLFT7C201BWX3Z
HTNRPVY01ANTI8
HTNRPVY01BKM1H

**GGT**

HLFT7C201BSJOU
HTNRPVY01ATDJN

**TRX**

| Contig1513 | HTNRPVY01A0MK7 | HLFT7C201BKTZY | |  |
| --- | --- | --- | --- | --- |
| Contig1626 | HTNRPVY01A0Z59 | HLFT7C201BL5GE | |  |
| Contig1628 | HTNRPVY01A3J88 | HLFT7C201BMYH5 | |  |
| Contig2194 | HTNRPVY01A6COZ | HLFT7C201BNF6O | |  |
| Contig2922 | HTNRPVY01A6PIK | HLFT7C201BNQXJ | |  |
| Contig3028 | HTNRPVY01A7W6A | HLFT7C201BO4PO | |  |
| Contig3366 | HTNRPVY01A7X5A | HLFT7C201BSG8B | |  |
| Contig3500 | HTNRPVY01A82YV | HLFT7C201BVATY | |  |
| Contig3608 | HTNRPVY01ABFRC | HLFT7C201BYSTF | |  |
| HLFT7C201A0PVO | HTNRPVY01AD6AK |  |  |  |
| HLFT7C201A1L4R | HTNRPVY01AEPPW | |  |  |
| HLFT7C201A3ZAQ | HTNRPVY01AGFOZ |  |  |  |
| HLFT7C201A4934 | HTNRPVY01AH9XC |  |  |  |
| HLFT7C201A4XK1 | HTNRPVY01AI9IQ |  |  |  |
| HLFT7C201A6139 | HTNRPVY01AJI5F |  |  |  |
| HLFT7C201AAZQ3 | HTNRPVY01AKKVF |  |  |  |
| HLFT7C201ACIB2 | HTNRPVY01ALFZL |  |  |  |
| HLFT7C201AEFXI | HTNRPVY01AM7H7 |  |  |  |
| HLFT7C201AG8JO | HTNRPVY01ANEK1 |  |  |  |
| HLFT7C201AH6U9 | HTNRPVY01AQ2Q7 |  |  |  |
| HLFT7C201AI8TT | HTNRPVY01ASKIR |  |  |  |
| HLFT7C201AIQ1D | HTNRPVY01ATF9S |  |  |  |
| HLFT7C201AJLWG | HTNRPVY01ATO9K |  |  |  |
| HLFT7C201AOL6X | HTNRPVY01AUOOG |  |  |  |
| HLFT7C201AST20 | HTNRPVY01BA5UW |  |  |  |
| HLFT7C201AVKHU | HTNRPVY01BADU4 |  |  |  |
| HLFT7C201AVS6W | HTNRPVY01BBL93 |  |  |  |
| HLFT7C201AWVV1 | HTNRPVY01BF52R |  |  |  |
| HLFT7C201AY1LE | HTNRPVY01BGLV5 |  |  |  |
| HLFT7C201AYI2G | HTNRPVY01BI8XX |  |  |  |
| HLFT7C201B02FC | HTNRPVY01BIXB3 |  |  |  |
| HLFT7C201B0CRL | HTNRPVY01BLJPL |  |  |  |
| HLFT7C201B1E9X | HTNRPVY01BLVVF |  |  |  |
| HLFT7C201B2HFH | HTNRPVY01BN9UI |  |  |  |
| HLFT7C201BESMS | HTNRPVY01BQ14P |  |  |  |
| HLFT7C201BFZXX | HTNRPVY01BR3ZR |  |  |  |
| HLFT7C201BHESC | HTNRPVY01BTSC6 |  |  |  |
| HLFT7C201BJU20 | HTNRPVY01BXKVV |  |  |  |

**ABC**

Contig1251
Contig2841
Contig3496
Contig3975
HLFT7C201A0JZ1
HLFT7C201A1BRM
HLFT7C201A5QBD
HLFT7C201A81XE
HLFT7C201AIQFP
HLFT7C201AKARM
HLFT7C201ALGXU
HLFT7C201ARRFX
HLFT7C201AX515
HLFT7C201B0JJM
HLFT7C201BB2E3
HLFT7C201BDFXP
HLFT7C201BG88C
HTNRPVY01A0P7C
HTNRPVY01A0ZZM
HTNRPVY01A6PAE
HTNRPVY01AB111
HTNRPVY01ACHK6
HTNRPVY01AGST1
HTNRPVY01AIMWP
HTNRPVY01APK1X
HTNRPVY01ATGCM
HTNRPVY01AW5BN
HTNRPVY01AYGM3
HTNRPVY01B0VS0
HTNRPVY01B2QM3
HTNRPVY01BA0IO
HTNRPVY01BBYRC
HTNRPVY01BE7PU
HTNRPVY01BKBKQ
HTNRPVY01BKEA6
HTNRPVY01BNQ7B
HTNRPVY01BRR3E
HTNRPVY01BSNI9

**CYP450**

| \| Contig1341 \| \| HTNRPVY01ADC9L \| HTNRPVY01A32FB \| \| \| --- \| --- \| --- \| --- \| --- \| \| Contig2402 \| \| HTNRPVY01AG15K \| HTNRPVY01A3DDV \| \| \| Contig2885 \| \| HTNRPVY01AH695 \| HTNRPVY01A3JR5 \| \| \| Contig3548 \| \| HTNRPVY01AIP9W \| HTNRPVY01A53Q7 \| \| \| Contig3549 \| \| HTNRPVY01AK7KG \| HTNRPVY01A7B60 \| \| \| Contig3650 \| \| HTNRPVY01AKKYO \| HTNRPVY01A8G0A \| \| \| Contig4072 \| \| HTNRPVY01AM03F \| HTNRPVY01AC38W \| \| \| Contig494 \|  \| HTNRPVY01AMOP9 \|  \|  \| \| Contig797 \|  \| HTNRPVY01AMPBU \|  \|  \| \| Contig916 \|  \| HTNRPVY01ANWEF \|  \|  \| \| HLFT7C201A14OT \| \| HTNRPVY01AO44M \|  \|  \| \| HLFT7C201A6U39 \| \| HTNRPVY01AOFOC \|  \|  \| \| HLFT7C201A82F3 \| \| HTNRPVY01AQI4V \|  \|  \| \| HLFT7C201A8YR1 \| \| HTNRPVY01AR4TD \|  \|  \| \| HLFT7C201ADDOT \| \| HTNRPVY01ATMFE \|  \|  \| \| HLFT7C201AEY2H \| \| HTNRPVY01ATMKH \|  \|  \| \| HLFT7C201AJY8K \| \| HTNRPVY01AU08Y \|  \|  \| \| HLFT7C201ANHPC \| \| HTNRPVY01AUIHM \|  \|  \| \| HLFT7C201ATOZ3 \| \| HTNRPVY01AVAVK \|  \|  \| \| HLFT7C201AVD44 \| \| HTNRPVY01AY875 \|  \|  \| \| HLFT7C201AVDRW \| \| HTNRPVY01B11W9 \|  \|  \| \| HLFT7C201AYYL2 \| \| HTNRPVY01BAD9L \|  \|  \| \| HLFT7C201AZOJ1 \| \| HTNRPVY01BBPBJ \|  \|  \| \| HLFT7C201BANAT \| \| HTNRPVY01BCBDD \|  \|  \| \| HLFT7C201BDPUM \| \| HTNRPVY01BCERQ \|  \|  \| \| HLFT7C201BGMED \| \| HTNRPVY01BEVI7 \|  \|  \| \| HLFT7C201BGYUU \| \| HTNRPVY01BFW04 \|  \|  \| \| HLFT7C201BIPO5 \| \| HTNRPVY01BGTAP \|  \|  \| \| HLFT7C201BK9KZ \| \| HTNRPVY01BI9G4 \|  \|  \| \| HLFT7C201BN8OR \| \| HTNRPVY01BJ8MY \|  \|  \| \| HLFT7C201BOSD5 \| \| HTNRPVY01BJMEI \|  \|  \| \| HLFT7C201BPT2Q \| \| HTNRPVY01BJUZF \|  \|  \| \| HLFT7C201BPXYO \| \| HTNRPVY01BKJ0Q \|  \|  \| \| HLFT7C201BS5RA \| \| HTNRPVY01BN2LH \|  \|  \| \| HLFT7C201BS8SP \| \| HTNRPVY01BNKGD \|  \|  \| \| HLFT7C201BUNIZ \| \| HTNRPVY01BNQPG \|  \|  \| \| HLFT7C201BXJNW \| \| HTNRPVY01BOVNB \|  \|  \| \| HLFT7C201BYB25 \| \| HTNRPVY01BR3Y2 \|  \|  \| \| HLFT7C201BYOPT \| \| HTNRPVY01BTVBJ \|  \|  \| \| HLFT7C201BYPDE \| \| HTNRPVY01BV5MG \|  \|  \| \| HLFT7C201BZHUT \| \| HTNRPVY01BVVVO \|  \|  \| \| HLFT7C201BZKSE \| \| HTNRPVY01BW71S \|  \|  \| \| HTNRPVY01A03L8 \| \| HTNRPVY01BX57X \|  \|  \| \| HTNRPVY01A0M4H \| \| HTNRPVY01BZ4C6 \|  \|  \| |
| --- | --- | --- | --- | --- | --- | --- | --- | --- | --- | --- | --- | --- | --- | --- | --- | --- | --- | --- | --- | --- | --- | --- | --- | --- | --- | --- | --- | --- | --- | --- | --- | --- | --- | --- | --- | --- | --- | --- | --- | --- | --- | --- | --- | --- | --- | --- | --- | --- | --- | --- | --- | --- | --- | --- | --- | --- | --- | --- | --- | --- | --- | --- | --- | --- | --- | --- | --- | --- | --- | --- | --- | --- | --- | --- | --- | --- | --- | --- | --- | --- | --- | --- | --- | --- | --- | --- | --- | --- | --- | --- | --- | --- | --- | --- | --- | --- | --- | --- | --- | --- | --- | --- | --- | --- | --- | --- | --- | --- | --- | --- | --- | --- | --- | --- | --- | --- | --- | --- | --- | --- | --- | --- | --- | --- | --- | --- | --- | --- | --- | --- | --- | --- | --- | --- | --- | --- | --- | --- | --- | --- | --- | --- | --- | --- | --- | --- | --- | --- | --- | --- | --- | --- | --- | --- | --- | --- | --- | --- | --- | --- | --- | --- | --- | --- | --- | --- | --- | --- | --- | --- | --- | --- | --- | --- | --- | --- | --- | --- | --- | --- | --- | --- | --- | --- | --- | --- | --- | --- | --- | --- | --- | --- | --- | --- | --- | --- | --- | --- | --- | --- | --- | --- | --- | --- | --- | --- | --- | --- | --- | --- | --- | --- | --- | --- | --- | --- | --- | --- | --- | --- |
|  |
|  |
|  |
|  |
|  |
|  |
|  |

**HSP70**

| Contig1244 | HLFT7C201AWFJ6 | HTNRPVY01AA9V8 | HTNRPVY01BRL04 | |  |
| --- | --- | --- | --- | --- | --- |
| Contig1481 | HLFT7C201AXJ5F | HTNRPVY01ADGI4 | HTNRPVY01BSQP9 | |  |
| Contig1697 | HLFT7C201BAP4O | HTNRPVY01AEUW9 | HTNRPVY01BTB95 | |  |
| Contig2015 | HLFT7C201BBCNU | HTNRPVY01AFKRL | HTNRPVY01BTSRJ | |  |
| Contig2080 | HLFT7C201BCH5M | HTNRPVY01AGCE7 | HTNRPVY01BU98G | |  |
| Contig2232 | HLFT7C201BCXKK | HTNRPVY01AHLGO | HTNRPVY01BUNM8 | | |
| Contig2270 | HLFT7C201BD8RK | HTNRPVY01AIEFU | HTNRPVY01BVPYF | |  |
| Contig2361 | HLFT7C201BEXWM | HTNRPVY01AIF90 | HTNRPVY01BWIFX | |  |
| Contig2410 | HLFT7C201BHAYG | HTNRPVY01AK29Y | HTNRPVY01BWUOB | | |
| Contig2985 | HLFT7C201BHE98 | HTNRPVY01AKXWT | HTNRPVY01BXWCT | | |
| Contig3021 | HLFT7C201BJ5IC | HTNRPVY01AL50U | HTNRPVY01BY2FU | |  |
| Contig4458 | HLFT7C201BJF4X | HTNRPVY01ALSXR | HTNRPVY01BYAEU | | |
| Contig583 | HLFT7C201BJMFU | HTNRPVY01AM1FS | HTNRPVY01BYLUW | | |
| Contig877 | HLFT7C201BL1TF | HTNRPVY01ANTES | HTNRPVY01BZ05L | |  |
| HLFT7C201A171E | HLFT7C201BLAI1 | HTNRPVY01AOSHX | HTNRPVY01BZ953 | |  |
| HLFT7C201A1BQY | HLFT7C201BME48 | HTNRPVY01AOU00 | HTNRPVY01BZN77 | |  |
| HLFT7C201A1UVR | HLFT7C201BNJOD | HTNRPVY01APK32 |  |  |  |
| HLFT7C201A2E28 | HLFT7C201BO77D | HTNRPVY01AQNC3 |  |  |  |
| HLFT7C201A2LAH | HLFT7C201BOYE1 | HTNRPVY01ASQ82 |  |  |  |
| HLFT7C201A3C9P | HLFT7C201BPKPW | HTNRPVY01ATU4J |  |  |  |
| HLFT7C201A468K | HLFT7C201BQG2D | HTNRPVY01AUBCY | |  |  |
| HLFT7C201A849R | HLFT7C201BQJDH | HTNRPVY01AXDO6 |  |  |  |
| HLFT7C201A8M03 | HLFT7C201BR1AD | HTNRPVY01B2D5C |  |  |  |
| HLFT7C201A94LK | HLFT7C201BTJOF | HTNRPVY01BA64J |  |  |  |
| HLFT7C201A9E27 | HLFT7C201BW31X | HTNRPVY01BA6F3 |  |  |  |
| HLFT7C201A9U2N | HTNRPVY01A02Z0 | HTNRPVY01BABFM | |  |  |
| HLFT7C201A9US4 | HTNRPVY01A0GY4 | HTNRPVY01BAWOK | |  |  |
| HLFT7C201AC6LC | HTNRPVY01A23BV | HTNRPVY01BBAC1 |  |  |  |
| HLFT7C201AF04J | HTNRPVY01A2TYW | HTNRPVY01BCLKB |  |  |  |
| HLFT7C201AIM3T | HTNRPVY01A3JCQ | HTNRPVY01BCW3A | |  |  |
| HLFT7C201ALAIF | HTNRPVY01A47R4 | HTNRPVY01BGR13 |  |  |  |
| HLFT7C201ALAUC | HTNRPVY01A4BVE | HTNRPVY01BK292 |  |  |  |
| HLFT7C201AM056 | HTNRPVY01A59B2 | HTNRPVY01BKGYL |  |  |  |
| HLFT7C201APFAQ | HTNRPVY01A8WR7 | HTNRPVY01BLVA5 |  |  |  |
| HLFT7C201AQQFK | HTNRPVY01A8YOK | HTNRPVY01BOPOL | |  |  |
| HLFT7C201AS0UQ | HTNRPVY01A90RW | HTNRPVY01BOVZN |  |  |  |
| HLFT7C201AS23T | HTNRPVY01A9LM8 | HTNRPVY01BPZYR |  |  |  |
| HLFT7C201AVAX2 | HTNRPVY01A9VFW | HTNRPVY01BR007 |  |  |  |
