# Supplementary material for: Gene Discovery through Transcriptome Sequencing for the Invasive Mussel Limnoperna fortunei
Source: PLoS One. 2014 Jul 21;9(7):e102973. doi: 10.1371/journal.pone.0102973 (PMC4105566; doi:10.1371/journal.pone.0102973)
Supplement: Table S6 — IDs of unigenes filtered out by NCBI. (DOCX) [file pone.0102973.s006.docx]

**SUPPORTING INFORMATION S6**

Uliano-Silva *et al.,* 2014. Gene discovery through transcriptome sequencing for the invasive mussel *Limnoperna fortunei.*

IDs of unigenes filtered out by NCBI criteria and thus did not submitted to the NCBI/TSA database. All the unigenes of *L. fortunei* are available for download at: **http://goo.gl/mNYPbX** .

|  | |  |  | |  | | |  |  |
| --- | --- | --- | --- | --- | --- | --- | --- | --- | --- |
|  | |  |  | |  | | |  |  |
|  | |  |  | |  | | |  |  |
| Contig1012 | | Contig3251 | HLFT7C201A70J6 | | HLFT7C201BAXH9 | | HLFT7C201BQL07 | | |
| Contig1019 | | Contig3275 | HLFT7C201A70KA | | HLFT7C201BB1UQ | | HLFT7C201BQMV4 | | |
| Contig1100 | | Contig3320 | HLFT7C201A728J | | HLFT7C201BBFBP | | HLFT7C201BQTPV | | |
| Contig1180 | | Contig3322 | HLFT7C201A7D6I | | HLFT7C201BBI3Y | | HLFT7C201BQYO3 | | |
| Contig1195 | | Contig3356 | HLFT7C201A7IFE | | HLFT7C201BBMO6 | | HLFT7C201BQZLI | | |
| Contig1196 | | Contig341 | HLFT7C201A7TW0 | | HLFT7C201BBR6D | | HLFT7C201BR6XC | | |
| Contig1217 | | Contig3410 | HLFT7C201A7WZM | | HLFT7C201BBS1R | | HLFT7C201BR97A | | |
| Contig1226 | | Contig3517 | HLFT7C201A816S | | HLFT7C201BBSGU | | HLFT7C201BRETO | | |
| Contig1230 | | Contig3577 | HLFT7C201A88MT | | HLFT7C201BBYXW | | HLFT7C201BREZ6 | | |
| Contig1261 | | Contig359 | HLFT7C201A89JQ | | HLFT7C201BC7P7 | | HLFT7C201BRK3M | | |
| Contig1262 | | Contig3596 | HLFT7C201A8J3R | | HLFT7C201BC9M6 | | HLFT7C201BRL1M | | |
| Contig127 | | Contig3644 | HLFT7C201A8M8E | | HLFT7C201BCA9F | | HLFT7C201BRXRS | | |
| Contig1272 | | Contig3698 | HLFT7C201A8MBN | | HLFT7C201BCEDK | | HLFT7C201BS9RB | | |
| Contig1273 | | Contig3719 | HLFT7C201A8Y3J | | HLFT7C201BCRGE | | HLFT7C201BSGQH | | |
| Contig1291 | | Contig375 | HLFT7C201A8YZ6 | | HLFT7C201BCTXV | | HLFT7C201BSMWP | | |
| Contig1302 | | HLFT7C201APQPJ | HLFT7C201A945K | | HLFT7C201BCUYD | | HLFT7C201BSNAW | | |
| Contig1309 | | HLFT7C201APXMJ | HLFT7C201A9RDT | | HLFT7C201BCW1N | | HLFT7C201BSQAS | | |
| Contig1396 | | HLFT7C201AQ10B | HLFT7C201A9XRA | | HLFT7C201BD2UP | | HLFT7C201BSQMK | | |
| Contig1406 | | HLFT7C201AQ2FZ | HLFT7C201AA32D | | HLFT7C201BD8L7 | | HLFT7C201BSXG9 | | |
| Contig143 | | HLFT7C201AQF35 | HLFT7C201AA3D1 | | HLFT7C201BDF0X | | HLFT7C201BT1LB | | |
| Contig1528 | | HLFT7C201AQHC4 | HLFT7C201AA59R | | HLFT7C201BDF9Y | | HLFT7C201BT425 | | |
| Contig153 | | HLFT7C201AQKIU | HLFT7C201AA6BB | | HLFT7C201BDG9C | | HLFT7C201BT4PD | | |
| Contig1536 | | HLFT7C201AQT0L | HLFT7C201AA87S | | HLFT7C201BDJCF | | HLFT7C201BT4PK | | |
| Contig1537 | | HLFT7C201AQTMG | HLFT7C201AADQK | | HLFT7C201BDSV5 | | HLFT7C201BT4UB | | |
| Contig1581 | | HLFT7C201AQVPZ | HLFT7C201AAEIM | | HLFT7C201BDWDO | | HLFT7C201BT8DC | | |
| Contig16 |  | HLFT7C201AQZ9Z | HLFT7C201AAJ20 | | HLFT7C201BDZD0 | | HLFT7C201BTCBN | | |
| Contig1663 | | HLFT7C201AR04T | HLFT7C201AAJ6J | | HLFT7C201BDZJI | | HLFT7C201BTE9O | | |
| Contig1724 | | HLFT7C201AR102 | HLFT7C201AAJXA | | HLFT7C201BE05J | | HLFT7C201BTFZU | | |
| Contig1822 | | HLFT7C201AR7TQ | HLFT7C201AAM80 | | HLFT7C201BE7VV | | HLFT7C201BTMD8 | | |
| Contig1876 | | HLFT7C201ARBOP | HLFT7C201AAT6C | | HLFT7C201BEBW0 | | HLFT7C201BTMHU | | |
| Contig1973 | | HLFT7C201ARCN2 | HLFT7C201AAWS6 | | HLFT7C201BEBZZ | | HLFT7C201BTMS1 | | |
| Contig1999 | | HLFT7C201ARLKK | HLFT7C201AAWSU | | HLFT7C201BECUE | | HLFT7C201BTOWL | | |
| Contig2 |  | HLFT7C201ARPCU | HLFT7C201ABCA4 | | HLFT7C201BEFEZ | | HLFT7C201BTPGH | | |
| Contig20 |  | HLFT7C201ARR73 | HLFT7C201ABGIA | | HLFT7C201BEFYM | | HLFT7C201BTS3K | | |
| Contig2011 | | HLFT7C201ARYKT | HLFT7C201ABIW4 | | HLFT7C201BEK6Z | | HLFT7C201BTX3Y | | |
| Contig2088 | | HLFT7C201AS0RA | HLFT7C201ABIXX | | HLFT7C201BEK9N | | HLFT7C201BUDU2 | | |
| Contig2162 | | HLFT7C201AS635 | HLFT7C201ABJRV | | HLFT7C201BEX82 | | HLFT7C201BUEHL | | |
| Contig2238 | | HLFT7C201ASNK3 | HLFT7C201ABPAU | | HLFT7C201BFGON | | HLFT7C201BUHBX | | |
| Contig2328 | | HLFT7C201ASNWT | HLFT7C201ABPT9 | | HLFT7C201BFJ94 | | HLFT7C201BUHN4 | | |
| Contig2348 | | HLFT7C201ASWQ2 | HLFT7C201ABSFR | | HLFT7C201BFP5I | | HLFT7C201BUK8H | | |
| Contig2445 | | HLFT7C201ATIRV | HLFT7C201ABVF1 | | HLFT7C201BFRJ4 | | HLFT7C201BUN4U | | |
| Contig2547 | | HLFT7C201ATLSE | HLFT7C201ABVQ0 | | HLFT7C201BFZLX | | HLFT7C201BUW4M | | |
| Contig2742 | | HLFT7C201ATSCC | HLFT7C201ABVTX | | HLFT7C201BG1P9 | | HLFT7C201BV1XI | | |
| Contig2774 | | HLFT7C201ATVIT | HLFT7C201ACBM7 | | HLFT7C201BG2YM | | HLFT7C201BV2I2 | | |
| Contig2781 | | HLFT7C201ATZR8 | HLFT7C201ACEPT | | HLFT7C201BG9AS | | HLFT7C201BV8ZZ | | |
| Contig2794 | | HLFT7C201AU0VJ | HLFT7C201ACH9I | | HLFT7C201BGAEG | | HLFT7C201BVD1E | | |
| Contig2907 | | HLFT7C201AU4WG | HLFT7C201ACLCB | | HLFT7C201BGC0H | | HLFT7C201BVD2X | | |
| Contig3153 | | HLFT7C201AU9ZL | HLFT7C201ACRDL | | HLFT7C201BGI6D | | HLFT7C201BVDN1 | | |
| Contig3212 | | HLFT7C201AUCCW | HLFT7C201ACRIF | | HLFT7C201BGJO2 | | HLFT7C201BVGFF | | |
| Contig3245 | | HLFT7C201AUHED | HLFT7C201ACUNU | | HLFT7C201BGLWK | | HLFT7C201BVMC2 | | |
| Contig325 | | HLFT7C201AUIF9 | HLFT7C201ACUZM | | HLFT7C201BGMQM | | HLFT7C201BVTAW | | |
| Contig3821 | | HLFT7C201AULAS | HLFT7C201ACXTA | | HLFT7C201BGPDR | | HLFT7C201BVTXO | | |
| Contig3823 | | HLFT7C201AULSR | HLFT7C201AD2DG | | HLFT7C201BGRZZ | | HLFT7C201BVWBK | | |
| Contig3831 | | HLFT7C201AUR8S | HLFT7C201AD83B | | HLFT7C201BGSM7 | | HLFT7C201BVYV0 | | |
| Contig4081 | | HLFT7C201AUT1K | HLFT7C201AD9HT | | HLFT7C201BGTI3 | | HLFT7C201BW02Z | | |
| Contig4118 | | HLFT7C201AV8IA | HLFT7C201ADDHT | | HLFT7C201BGU67 | | HLFT7C201BW1OG | | |
| Contig4122 | | HLFT7C201AVHI6 | HLFT7C201ADMH3 | | HLFT7C201BGWDV | | HLFT7C201BW4OV | | |
| Contig4142 | | HLFT7C201AVNRL | HLFT7C201ADS3U | | HLFT7C201BH1B0 | | HLFT7C201BWFI8 | | |
| Contig4155 | | HLFT7C201AVTK3 | HLFT7C201AE05S | | HLFT7C201BH4FY | | HLFT7C201BWLJE | | |
| Contig4265 | | HLFT7C201AVY2D | HLFT7C201AE35V | | HLFT7C201BH7LL | | HLFT7C201BWOOM | | |
| Contig4282 | | HLFT7C201AW4RX | HLFT7C201AE4HE | | HLFT7C201BH7ME | | HLFT7C201BWYYB | | |
| Contig4297 | | HLFT7C201AW7NW | HLFT7C201AE4SZ | | HLFT7C201BHD9V | | HLFT7C201BX0US | | |
| Contig432 | | HLFT7C201AWB3U | HLFT7C201AE4VF | | HLFT7C201BHEB1 | | HLFT7C201BX5RL | | |
| Contig4327 | | HLFT7C201AWUTF | HLFT7C201AE7U7 | | HLFT7C201BHEWG | | HLFT7C201BX824 | | |
| Contig4377 | | HLFT7C201AWYYF | HLFT7C201AEBRI | | HLFT7C201BHHWZ | | HLFT7C201BXHCX | | |
| Contig4416 | | HLFT7C201AX05B | HLFT7C201AECJD | | HLFT7C201BHKIU | | HLFT7C201BXHG2 | | |
| Contig4429 | | HLFT7C201AX2VJ | HLFT7C201AEE01 | | HLFT7C201BHNN6 | | HLFT7C201BXM2D | | |
| Contig4436 | | HLFT7C201AX6MK | HLFT7C201AEEWK | | HLFT7C201BHOLU | | HLFT7C201BXXNA | | |
| Contig4452 | | HLFT7C201AX9PE | HLFT7C201AEEWR | | HLFT7C201BHRA3 | | HLFT7C201BXZ61 | | |
| Contig4455 | | HLFT7C201AXA5M | HLFT7C201AEOO5 | | HLFT7C201BHU77 | | HLFT7C201BY4KU | | |
| Contig4458 | | HLFT7C201AXAMI | HLFT7C201AERIY | | HLFT7C201BHUUA | | HLFT7C201BYCB2 | | |
| Contig4467 | | HLFT7C201AXG54 | HLFT7C201AESFL | | HLFT7C201BI89F | | HLFT7C201BYLPI | | |
| Contig4472 | | HLFT7C201AXT0O | HLFT7C201AFDQ4 | | HLFT7C201BIDKW | | HLFT7C201BYO3M | | |
| Contig4476 | | HLFT7C201AY121 | HLFT7C201AFM2V | | HLFT7C201BIJMD | | HLFT7C201BYO47 | | |
| Contig4485 | | HLFT7C201AY16B | HLFT7C201AFNIQ | | HLFT7C201BIPM9 | | HLFT7C201BYOWI | | |
| Contig4488 | | HLFT7C201AY1LE | HLFT7C201AFQMR | | HLFT7C201BIW2J | | HLFT7C201BYR18 | | |
| Contig4497 | | HLFT7C201AY1ZL | HLFT7C201AG24E | | HLFT7C201BIW3G | | HLFT7C201BYU7N | | |
| Contig4515 | | HLFT7C201AY2W0 | HLFT7C201AG7ZD | | HLFT7C201BJ1CV | | HLFT7C201BYVSI | | |
| Contig4551 | | HLFT7C201AY5E7 | HLFT7C201AGF1N | | HLFT7C201BJ7Q4 | | HLFT7C201BZ0BI | | |
| Contig4561 | | HLFT7C201AY83R | HLFT7C201AGGB5 | | HLFT7C201BJC1M | | HLFT7C201BZ1MC | | |
| Contig4601 | | HLFT7C201AYGUE | HLFT7C201AGZCG | | HLFT7C201BJIEQ | | HLFT7C201BZ3LA | | |
| Contig57 |  | HLFT7C201AYIZE | HLFT7C201AH986 | | HLFT7C201BJLZM | | HLFT7C201BZ3LO | | |
| Contig576 | | HLFT7C201AYS9V | HLFT7C201AHCH0 | | HLFT7C201BJOX5 | | HLFT7C201BZ6IK | | |
| Contig6 |  | HLFT7C201AYVJ1 | HLFT7C201AHE73 | | HLFT7C201BJR0Y | | HLFT7C201BZ979 | | |
| Contig629 | | HLFT7C201AYVUD | HLFT7C201AHE7S | | HLFT7C201BJU1B | | HLFT7C201BZEZI | | |
| Contig65 |  | HLFT7C201AYYZD | HLFT7C201AHIUN | | HLFT7C201BJUN3 | | HLFT7C201BZKRB | | |
| Contig656 | | HLFT7C201AZ32W | HLFT7C201AHKR5 | | HLFT7C201BK9ZN | | HLFT7C201BZKTO | | |
| Contig749 | | HLFT7C201AZD7A | HLFT7C201AHU53 | | HLFT7C201BKAW6 | | HLFT7C201BZNSH | | |
| Contig814 | | HLFT7C201AZLD7 | HLFT7C201AHUMP | | HLFT7C201BKH5J | | HLFT7C201BZOY8 | | |
| Contig847 | | HLFT7C201AZN3V | HLFT7C201AHXBN | | HLFT7C201BKNAC | | HLFT7C201BZQ22 | | |
| Contig861 | | HLFT7C201B02LU | HLFT7C201AHXSP | | HLFT7C201BKNBR | | HLFT7C201BZQ2O | | |
| Contig869 | | HLFT7C201B048Z | HLFT7C201AI0A7 | | HLFT7C201BKNOE | | HLFT7C201BZQPV | | |
| Contig895 | | HLFT7C201B05Z1 | HLFT7C201AI9KN | | HLFT7C201BKQ0B | | HLFT7C201BZQQM | | |
| Contig96 |  | HLFT7C201B08RA | HLFT7C201AIANC | | HLFT7C201BKWSX | | HLFT7C201BZQTZ | | |
| HLFT7C201A02AP | | HLFT7C201B08TA | HLFT7C201AIJ7Z | | HLFT7C201BL5D9 | | HLFT7C201BZT8T | | |
| HLFT7C201A03KN | | HLFT7C201B0DBK | HLFT7C201AIJD2 | | HLFT7C201BL5S5 | | HLFT7C201BZTWR | | |
| HLFT7C201A0532 | | HLFT7C201B0F37 | HLFT7C201AIJGN | | HLFT7C201BL8BU | | HLFT7C201BZW1G | | |
| HLFT7C201A09VO | | HLFT7C201B0GDS | HLFT7C201AIP96 | | HLFT7C201BLIS8 | | HLFT7C201BZW1U | | |
| HLFT7C201A0JQM | | HLFT7C201B0JI0 | HLFT7C201AIS4A | | HLFT7C201BLP9U | | HLFT7C201BZW5H | | |
| HLFT7C201A0WOC | | HLFT7C201B0JRU | HLFT7C201AJ03Q | | HLFT7C201BLPFU | | HTNRPVY01A03S5 | | |
| HLFT7C201A0XCK | | HLFT7C201B0MFA | HLFT7C201AJ4LP | | HLFT7C201BLYI7 | | HTNRPVY01A05K7 | | |
| HLFT7C201A14OP | | HLFT7C201B0MPC | HLFT7C201AJ5NM | | HLFT7C201BM0LH | | HTNRPVY01A0D8Y | | |
| HLFT7C201A15JL | | HLFT7C201B0PYS | HLFT7C201AJFC4 | | HLFT7C201BM7BS | | HTNRPVY01A0F61 | | |
| HLFT7C201A1BQR | | HLFT7C201B0TCZ | HLFT7C201AJMSG | | HLFT7C201BM9Y5 | | HTNRPVY01A0G6B | | |
| HLFT7C201A1F60 | | HLFT7C201B0TLQ | HLFT7C201AJRMC | | HLFT7C201BMCL4 | | HTNRPVY01A0J4C | | |
| HLFT7C201A1LAL | | HLFT7C201B0VQI | HLFT7C201AJUT0 | | HLFT7C201BME5J | | HTNRPVY01A0NEQ | | |
| HLFT7C201A1LNV | | HLFT7C201B0VUV | HLFT7C201AK3PO | | HLFT7C201BMHRO | | HTNRPVY01A0PNN | | |
| HLFT7C201A1LX8 | | HLFT7C201B17T4 | HLFT7C201AK3ZZ | | HLFT7C201BMHTH | | HTNRPVY01A0QXT | | |
| HLFT7C201A1MKM | | HLFT7C201B1BKB | HLFT7C201AK9IA | | HLFT7C201BMINC | | HTNRPVY01A0TIT | | |
| HLFT7C201A1RR7 | | HLFT7C201B1H1U | HLFT7C201AKBYK | | HLFT7C201BMNZX | | HTNRPVY01A0TT1 | | |
| HLFT7C201A1SV6 | | HLFT7C201B1HUR | HLFT7C201AKD2A | | HLFT7C201BMR70 | | HTNRPVY01A0WON | | |
| HLFT7C201A1V4Y | | HLFT7C201B1K08 | HLFT7C201AKG8X | | HLFT7C201BMRRH | | HTNRPVY01A15NO | | |
| HLFT7C201A1XWK | | HLFT7C201B1N67 | HLFT7C201AL250 | | HLFT7C201BMUQF | | HTNRPVY01A17CP | | |
| HLFT7C201A27HF | | HLFT7C201B1OCF | HLFT7C201AL5E6 | | HLFT7C201BMXCD | | HTNRPVY01A17LT | | |
| HLFT7C201A2DZ8 | | HLFT7C201B1OKR | HLFT7C201AL741 | | HLFT7C201BMXEF | | HTNRPVY01A18FV | | |
| HLFT7C201A2HBF | | HLFT7C201B1RCU | HLFT7C201AL9JM | | HLFT7C201BMXEP | | HTNRPVY01A1IF0 | | |
| HLFT7C201A2HQK | | HLFT7C201B1RGF | HLFT7C201ALASI | | HLFT7C201BMXOM | | HTNRPVY01A1MAX | | |
| HLFT7C201A2NIM | | HLFT7C201B1RQT | HLFT7C201ALG0M | | HLFT7C201BN3EY | | HTNRPVY01A1MCT | | |
| HLFT7C201A31XN | | HLFT7C201B1U71 | HLFT7C201ALI6D | | HLFT7C201BN9GO | | HTNRPVY01A1MLF | | |
| HLFT7C201A3GIX | | HLFT7C201B1XT5 | HLFT7C201ALO94 | | HLFT7C201BND8N | | HTNRPVY01A1OYI | | |
| HLFT7C201A3LZQ | | HLFT7C201B23YE | HLFT7C201ALPM8 | | HLFT7C201BNPS3 | | HTNRPVY01A1PVK | | |
| HLFT7C201A3MIU | | HLFT7C201B2879 | HLFT7C201ALSPN | | HLFT7C201BNQ1S | | HTNRPVY01A1SRX | | |
| HLFT7C201A3SWA | | HLFT7C201B2AAV | HLFT7C201ALWBS | | HLFT7C201BNQE7 | | HTNRPVY01A1UUO | | |
| HLFT7C201A4018 | | HLFT7C201B2APB | HLFT7C201AM602 | | HLFT7C201BNV5L | | HTNRPVY01A20AN | | |
| HLFT7C201A406N | | HLFT7C201B2ARP | HLFT7C201AMHRJ | | HLFT7C201BNWKI | | HTNRPVY01A258R | | |
| HLFT7C201A44TN | | HLFT7C201B2AZU | HLFT7C201AMHY4 | | HLFT7C201BNXEO | | HTNRPVY01A2AUP | | |
| HLFT7C201A496F | | HLFT7C201B2D2T | HLFT7C201AMK02 | | HLFT7C201BNXFU | | HTNRPVY01A2BW9 | | |
| HLFT7C201A4BHM | | HLFT7C201B2D5K | HLFT7C201AMK2H | | HLFT7C201BNY6Y | | HTNRPVY01A2E5N | | |
| HLFT7C201A4BJ7 | | HLFT7C201B2D5N | HLFT7C201AMRKF | | HLFT7C201BO1VX | | HTNRPVY01A2H13 | | |
| HLFT7C201A4CHE | | HLFT7C201B2D5Y | HLFT7C201AN5QQ | | HLFT7C201BO2CI | | HTNRPVY01A2OOC | | |
| HLFT7C201A4FLR | | HLFT7C201B2GQI | HLFT7C201AN87F | | HLFT7C201BOIHW | | HTNRPVY01A2RG6 | | |
| HLFT7C201A4K41 | | HLFT7C201B2J4F | HLFT7C201ANAAU | | HLFT7C201BOLSI | | HTNRPVY01A2RSQ | | |
| HLFT7C201A4N4Q | | HLFT7C201B2J6I | HLFT7C201ANAWF | | HLFT7C201BOMKO | | HTNRPVY01A2WUB | | |
| HLFT7C201A4R9X | | HLFT7C201B2QOL | HLFT7C201ANHTU | | HLFT7C201BOR8H | | HTNRPVY01A2XL4 | | |
| HLFT7C201A4VNA | | HLFT7C201B2TVT | HLFT7C201ANJ6R | | HLFT7C201BOYTA | | HTNRPVY01A3802 | | |
| HLFT7C201A52GL | | HLFT7C201B3C20 | HLFT7C201ANJTZ | | HLFT7C201BP94Q | | HTNRPVY01A3FKP | | |
| HLFT7C201A5569 | | HLFT7C201B3F7C | HLFT7C201ANJZ3 | | HLFT7C201BPA9Z | | HTNRPVY01A3FOQ | | |
| HLFT7C201A55ZO | | HLFT7C201B3IPE | HLFT7C201ANM0G | | HLFT7C201BPAZO | | HTNRPVY01A3FS7 | | |
| HLFT7C201A5JHD | | HLFT7C201B3LVR | HLFT7C201AO2IQ | | HLFT7C201BPEIA | | HTNRPVY01A3GD9 | | |
| HLFT7C201A5NJE | | HLFT7C201B3OZL | HLFT7C201AO5T6 | | HLFT7C201BPEY9 | | HTNRPVY01A3MHG | | |
| HLFT7C201A5S9E | | HLFT7C201B3R6J | HLFT7C201AO8AV | | HLFT7C201BPKIV | | HTNRPVY01A3Y19 | | |
| HLFT7C201A5TJL | | HLFT7C201B3SGT | HLFT7C201AOCA1 | | HLFT7C201BPTLU | | HTNRPVY01A3Z02 | | |
| HLFT7C201A5TLI | | HLFT7C201B3V39 | HLFT7C201AOVMJ | | HLFT7C201BPXI0 | | HTNRPVY01A47Y9 | | |
| HLFT7C201A64K1 | | HLFT7C201B3VHM | HLFT7C201AOYBE | | HLFT7C201BQ18M | | HTNRPVY01A4ERJ | | |
| HLFT7C201A65LI | | HLFT7C201BA63R | HLFT7C201AP4PR | | HLFT7C201BQ1Q4 | | HTNRPVY01A4HMG | | |
| HLFT7C201A67H1 | | HLFT7C201BAC88 | HLFT7C201APBS2 | | HLFT7C201BQ29K | | HTNRPVY01A4LUI | | |
| HLFT7C201A6C1T | | HLFT7C201BAG08 | HLFT7C201APE40 | | HLFT7C201BQ2K5 | | HTNRPVY01A4U5F | | |
| HLFT7C201A6ROD | | HLFT7C201BAGG1 | HLFT7C201APH0M | | HLFT7C201BQ8KA | | HTNRPVY01A4UPO | | |
| HLFT7C201A6V34 | | HLFT7C201BAHUR | HLFT7C201APK0B | | HLFT7C201BQ8VE | | HTNRPVY01A4UTE | | |
| HLFT7C201A6YNV | | HLFT7C201BAP3D | HLFT7C201APNEF | | HLFT7C201BQCH9 | | HTNRPVY01A4UYL | | |
| HLFT7C201A70HC | | HLFT7C201BAQ9G | HLFT7C201APNRT | | HLFT7C201BQJHJ | | HTNRPVY01A4X0Q | | |
| HTNRPVY01A56VM | | HTNRPVY01A7UHT | HTNRPVY01AC3XN | | HTNRPVY01AGMT0 | | HTNRPVY01AJMCA | | |
| HTNRPVY01A5AHR | | HTNRPVY01A7ZYA | HTNRPVY01AC3ZC | | HTNRPVY01AGP3Q | | HTNRPVY01AK1EM | | |
| HTNRPVY01A5HGT | | HTNRPVY01A89F0 | HTNRPVY01ACE8D | | HTNRPVY01AGQAT | | HTNRPVY01AK6O1 | | |
| HTNRPVY01A5JXG | | HTNRPVY01A8FTG | HTNRPVY01ACHDF | | HTNRPVY01AGVIA | | HTNRPVY01AK7KG | | |
| HTNRPVY01A5KCI | | HTNRPVY01A8I7F | HTNRPVY01ACISK | | HTNRPVY01AGVK2 | | HTNRPVY01AK96K | | |
| HTNRPVY01A5KXG | | HTNRPVY01A8P2P | HTNRPVY01ACN0F | | HTNRPVY01AGVQD | | HTNRPVY01AKAUA | | |
| HTNRPVY01A5MU5 | | HTNRPVY01A8SNV | HTNRPVY01ACXCY | | HTNRPVY01AGWA2 | | HTNRPVY01AKHCK | | |
| HTNRPVY01A5N3H | | HTNRPVY01A8WLT | HTNRPVY01ACXHT | | HTNRPVY01AGWVD | | HTNRPVY01AKJ4B | | |
| HTNRPVY01A5QW7 | | HTNRPVY01A90YC | HTNRPVY01ACXQD | | HTNRPVY01AGYTE | | HTNRPVY01AKKUW | | |
| HTNRPVY01A5S82 | | HTNRPVY01A91LD | HTNRPVY01ACYGG | | HTNRPVY01AGZFE | | HTNRPVY01AKNLE | | |
| HTNRPVY01A5WJ5 | | HTNRPVY01A932K | HTNRPVY01AD0CC | | HTNRPVY01AH0NT | | HTNRPVY01AKQHT | | |
| HTNRPVY01A5XCY | | HTNRPVY01A94OE | HTNRPVY01AD5OS | | HTNRPVY01AH4J9 | | HTNRPVY01AKUW3 | | |
| HTNRPVY01A5ZOV | | HTNRPVY01A94ZL | HTNRPVY01ADA3I | | HTNRPVY01AH66T | | HTNRPVY01AL423 | | |
| HTNRPVY01A6061 | | HTNRPVY01A962U | HTNRPVY01ADGFI | | HTNRPVY01AH7OL | | HTNRPVY01AL8CV | | |
| HTNRPVY01A6112 | | HTNRPVY01A9CBL | HTNRPVY01ADGUX | | HTNRPVY01AHHOB | | HTNRPVY01ALFUQ | | |
| HTNRPVY01A6IYJ | | HTNRPVY01A9CQ5 | HTNRPVY01ADHEP | | HTNRPVY01AHIG5 | | HTNRPVY01ALGZE | | |
| HTNRPVY01A6MUJ | | HTNRPVY01A9ELA | HTNRPVY01ADKG7 | | HTNRPVY01AHLVF | | HTNRPVY01ALJVD | | |
| HTNRPVY01A6SJR | | HTNRPVY01A9LG9 | HTNRPVY01ADVVW | | HTNRPVY01AHQ30 | | HTNRPVY01ALL6Y | | |
| HTNRPVY01A6V6H | | HTNRPVY01A9O7P | HTNRPVY01AE5HZ | | HTNRPVY01AHX1B | | HTNRPVY01ALMM4 | | |
| HTNRPVY01A6VFD | | HTNRPVY01A9Q5O | HTNRPVY01AEF9H | | HTNRPVY01AHXFA | | HTNRPVY01ALO7K | | |
| HTNRPVY01A6VUB | | HTNRPVY01A9UJS | HTNRPVY01AEK7J | | HTNRPVY01AI25C | | HTNRPVY01ALSO6 | | |
| HTNRPVY01A6X7Z | | HTNRPVY01AAQYJ | HTNRPVY01AEU5T | | HTNRPVY01AI518 | | HTNRPVY01ALTD3 | | |
| HTNRPVY01A6YHF | | HTNRPVY01AAREO | HTNRPVY01AEVP8 | | HTNRPVY01AIAKN | | HTNRPVY01ALTIZ | | |
| HTNRPVY01A6YQG | | HTNRPVY01AATOO | HTNRPVY01AEVWS | | HTNRPVY01AIGRL | | HTNRPVY01ALWDX | | |
| HTNRPVY01A701G | | HTNRPVY01AATPM | HTNRPVY01AEY5D | | HTNRPVY01AIQUA | | HTNRPVY01ALZSI | | |
| HTNRPVY01A70H9 | | HTNRPVY01AATZV | HTNRPVY01AF0D8 | | HTNRPVY01AIQVJ | | HTNRPVY01AM1ZU | | |
| HTNRPVY01A71B7 | | HTNRPVY01AAUFE | HTNRPVY01AF0ET | | HTNRPVY01AIWHA | | HTNRPVY01AM414 | | |
| HTNRPVY01A7ARL | | HTNRPVY01AAXMR | HTNRPVY01AF7CO | | HTNRPVY01AIZ98 | | HTNRPVY01AM7MF | | |
| HTNRPVY01A7FCG | | HTNRPVY01AB4ST | HTNRPVY01AFLB5 | | HTNRPVY01AJ2B8 | | HTNRPVY01AM93W | | |
| HTNRPVY01A7G1U | | HTNRPVY01AB86V | HTNRPVY01AFWLM | | HTNRPVY01AJ5I7 | | HTNRPVY01AMCRN | | |
| HTNRPVY01A7HLG | | HTNRPVY01ABF4B | HTNRPVY01AFWO6 | | HTNRPVY01AJ5N1 | | HTNRPVY01AMFCW | | |
| HTNRPVY01A7NEF | | HTNRPVY01ABFFL | HTNRPVY01AG150 | | HTNRPVY01AJ7FI | | HTNRPVY01AMFM4 | | |
| HTNRPVY01A7NLE | | HTNRPVY01ABLS5 | HTNRPVY01AG1ZF | | HTNRPVY01AJ7XX | | HTNRPVY01AMKTQ | | |
| HTNRPVY01A7QH0 | | HTNRPVY01ABLTT | HTNRPVY01AG8AH | | HTNRPVY01AJDAC | | HTNRPVY01AMLYS | | |
| HTNRPVY01A7QMP | | HTNRPVY01ABMXE | HTNRPVY01AGC0J | | HTNRPVY01AJE66 | | HTNRPVY01AMN85 | | |
| HTNRPVY01A7RC5 | | HTNRPVY01ABU6C | HTNRPVY01AGCYD | | HTNRPVY01AJJH3 | | HTNRPVY01AMRY4 | | |
| HTNRPVY01A7TVC | | HTNRPVY01AC1MZ | HTNRPVY01AGDFM | | HTNRPVY01AJL10 | | HTNRPVY01AMSB8 | | |
| HTNRPVY01BS5LW | | HTNRPVY01AV6BT | HTNRPVY01B2NFT | | HTNRPVY01BFXNK | | HTNRPVY01AMVA4 | | |
| HTNRPVY01BS6NG | | HTNRPVY01AV8HZ | HTNRPVY01B2NOC | | HTNRPVY01BFZRB | | HTNRPVY01AMXO6 | | |
| HTNRPVY01BS9OB | | HTNRPVY01AV8XC | HTNRPVY01B2Z5V | | HTNRPVY01BG5E8 | | HTNRPVY01AN2XQ | | |
| HTNRPVY01BSD5G | | HTNRPVY01AVC4A | HTNRPVY01B3AA8 | | HTNRPVY01BG77K | | HTNRPVY01ANDQE | | |
| HTNRPVY01BSGLK | | HTNRPVY01AVF4A | HTNRPVY01B3YX6 | | HTNRPVY01BG7XL | | HTNRPVY01ANENE | | |
| HTNRPVY01BSGTM | | HTNRPVY01AVGGG | HTNRPVY01BA3YM | | HTNRPVY01BGCD2 | | HTNRPVY01ANGIC | | |
| HTNRPVY01BSNUL | | HTNRPVY01AVJX2 | HTNRPVY01BA59K | | HTNRPVY01BGCQH | | HTNRPVY01ANNBV | | |
| HTNRPVY01BSQW6 | | HTNRPVY01AVJZI | HTNRPVY01BAHYD | | HTNRPVY01BGDHX | | HTNRPVY01ANNE4 | | |
| HTNRPVY01BSTRF | | HTNRPVY01AVMGH | HTNRPVY01BAHYQ | | HTNRPVY01BGII6 | | HTNRPVY01ANP0Q | | |
| HTNRPVY01BT1J4 | | HTNRPVY01AVP7D | HTNRPVY01BAM9L | | HTNRPVY01BGIQM | | HTNRPVY01ANQHZ | | |
| HTNRPVY01BT46V | | HTNRPVY01AVQAU | HTNRPVY01BAQ7J | | HTNRPVY01BGJWR | | HTNRPVY01ANTPT | | |
| HTNRPVY01BT8IY | | HTNRPVY01AVSSJ | HTNRPVY01BAQJJ | | HTNRPVY01BGLR7 | | HTNRPVY01ANZ67 | | |
| HTNRPVY01BTB62 | | HTNRPVY01AVSY9 | HTNRPVY01BAQOH | | HTNRPVY01BGLV5 | | HTNRPVY01AO5IZ | | |
| HTNRPVY01BTFD9 | | HTNRPVY01AVV5R | HTNRPVY01BARBG | | HTNRPVY01BGMAZ | | HTNRPVY01AO7WJ | | |
| HTNRPVY01BTFHG | | HTNRPVY01AVWUF | HTNRPVY01BATYD | | HTNRPVY01BGO2A | | HTNRPVY01AOCG6 | | |
| HTNRPVY01BTLNP | | HTNRPVY01AW05Q | HTNRPVY01BAUFD | | HTNRPVY01BGPZI | | HTNRPVY01AODC9 | | |
| HTNRPVY01BTLSL | | HTNRPVY01AW384 | HTNRPVY01BAV81 | | HTNRPVY01BGR7P | | HTNRPVY01AOFUK | | |
| HTNRPVY01BTO6O | | HTNRPVY01AW8P0 | HTNRPVY01BAZMF | | HTNRPVY01BGVCH | | HTNRPVY01AOIRQ | | |
| HTNRPVY01BTPQG | | HTNRPVY01AWFBG | HTNRPVY01BB1K9 | | HTNRPVY01BGVCK | | HTNRPVY01AOJLC | | |
| HTNRPVY01BTYDK | | HTNRPVY01AWIQR | HTNRPVY01BBDH3 | | HTNRPVY01BGVDB | | HTNRPVY01AOPI1 | | |
| HTNRPVY01BU0IA | | HTNRPVY01AWRSQ | HTNRPVY01BBF01 | | HTNRPVY01BGVDJ | | HTNRPVY01AOS38 | | |
| HTNRPVY01BU3LL | | HTNRPVY01AWUZ6 | HTNRPVY01BBFH5 | | HTNRPVY01BGWHF | | HTNRPVY01AOVO8 | | |
| HTNRPVY01BU4BV | | HTNRPVY01AX295 | HTNRPVY01BBMWB | | HTNRPVY01BH355 | | HTNRPVY01AOX33 | | |
| HTNRPVY01BU4JN | | HTNRPVY01AX37X | HTNRPVY01BBPCY | | HTNRPVY01BH389 | | HTNRPVY01AOX7N | | |
| HTNRPVY01BU62X | | HTNRPVY01AX6GK | HTNRPVY01BBPOI | | HTNRPVY01BH3QH | | HTNRPVY01AOY2M | | |
| HTNRPVY01BU6ZX | | HTNRPVY01AXDK4 | HTNRPVY01BBR21 | | HTNRPVY01BH7YU | | HTNRPVY01AOYNG | | |
| HTNRPVY01BUB19 | | HTNRPVY01AXEEZ | HTNRPVY01BBTDU | | HTNRPVY01BH9X6 | | HTNRPVY01AP0HL | | |
| HTNRPVY01BULDJ | | HTNRPVY01AXLA8 | HTNRPVY01BBVC5 | | HTNRPVY01BHBK5 | | HTNRPVY01AP0P5 | | |
| HTNRPVY01BUNEG | | HTNRPVY01AXREO | HTNRPVY01BBVK3 | | HTNRPVY01BHECN | | HTNRPVY01AP668 | | |
| HTNRPVY01BUNK9 | | HTNRPVY01AXT7T | HTNRPVY01BBY2Z | | HTNRPVY01BHH69 | | HTNRPVY01AP6RU | | |
| HTNRPVY01BUQ5R | | HTNRPVY01AXTCF | HTNRPVY01BC7TZ | | HTNRPVY01BHK1P | | HTNRPVY01APFAL | | |
| HTNRPVY01BURDK | | HTNRPVY01AXWLI | HTNRPVY01BC9RS | | HTNRPVY01BHKNP | | HTNRPVY01APICI | | |
| HTNRPVY01BUTM7 | | HTNRPVY01AXWQZ | HTNRPVY01BCFGM | | HTNRPVY01BHR9M | | HTNRPVY01APIJ7 | | |
| HTNRPVY01BUTU0 | | HTNRPVY01AY16U | HTNRPVY01BCH8N | | HTNRPVY01BHU9W | | HTNRPVY01APOTJ | | |
| HTNRPVY01BUWXS | | HTNRPVY01AY4YY | HTNRPVY01BCINN | | HTNRPVY01BHUM8 | | HTNRPVY01APXBJ | | |
| HTNRPVY01BUXCK | | HTNRPVY01AY8KE | HTNRPVY01BCKWA | | HTNRPVY01BI5LX | | HTNRPVY01APXDP | | |
| HTNRPVY01BV2NN | | HTNRPVY01AY8SD | HTNRPVY01BCN5T | | HTNRPVY01BI5O9 | | HTNRPVY01APXOJ | | |
| HTNRPVY01BV2R9 | | HTNRPVY01AYCZX | HTNRPVY01BCQLA | | HTNRPVY01BI8V9 | | HTNRPVY01AQ2H7 | | |
| HTNRPVY01BV9C0 | | HTNRPVY01AYDCW | HTNRPVY01BCQS5 | | HTNRPVY01BI9J9 | | HTNRPVY01AQ5BS | | |
| HTNRPVY01BV9NB | | HTNRPVY01AYGVK | HTNRPVY01BCRDP | | HTNRPVY01BIAGV | | HTNRPVY01AQAW5 | | |
| HTNRPVY01BVG5M | | HTNRPVY01AYI3U | HTNRPVY01BCRF7 | | HTNRPVY01BIDYV | | HTNRPVY01AQGMZ | | |
| HTNRPVY01BVJ5Y | | HTNRPVY01AYM6R | HTNRPVY01BCT7N | | HTNRPVY01BISW3 | | HTNRPVY01AQGT1 | | |
| HTNRPVY01BVJTN | | HTNRPVY01AYMLS | HTNRPVY01BCTSN | | HTNRPVY01BISWW | | HTNRPVY01AQJJL | | |
| HTNRPVY01BVM79 | | HTNRPVY01AYSGO | HTNRPVY01BCTVJ | | HTNRPVY01BIW1E | | HTNRPVY01AQM43 | | |
| HTNRPVY01BVO82 | | HTNRPVY01AYU86 | HTNRPVY01BCUJ9 | | HTNRPVY01BIZ64 | | HTNRPVY01AQS37 | | |
| HTNRPVY01BVPE2 | | HTNRPVY01AYVX7 | HTNRPVY01BD172 | | HTNRPVY01BIZQ1 | | HTNRPVY01AQS8Q | | |
| HTNRPVY01BVPYQ | | HTNRPVY01AYYE2 | HTNRPVY01BD5ES | | HTNRPVY01BJ40O | | HTNRPVY01AQWSP | | |
| HTNRPVY01BVSOL | | HTNRPVY01AZ0RX | HTNRPVY01BD8KF | | HTNRPVY01BJ443 | | HTNRPVY01AR0W7 | | |
| HTNRPVY01BVWQM | | HTNRPVY01AZ96S | HTNRPVY01BDCVJ | | HTNRPVY01BJ4W5 | | HTNRPVY01ARE00 | | |
| HTNRPVY01BVWTI | | HTNRPVY01AZFN2 | HTNRPVY01BDD3S | | HTNRPVY01BJ68H | | HTNRPVY01ARFFS | | |
| HTNRPVY01BW1H2 | | HTNRPVY01AZH01 | HTNRPVY01BDJ48 | | HTNRPVY01BJ8IO | | HTNRPVY01ARLU4 | | |
| HTNRPVY01BW96V | | HTNRPVY01AZH9W | HTNRPVY01BDJJW | | HTNRPVY01BJBMZ | | HTNRPVY01ARRG0 | | |
| HTNRPVY01BWCRE | | HTNRPVY01AZHES | HTNRPVY01BDMW7 | | HTNRPVY01BJCID | | HTNRPVY01ARVV1 | | |
| HTNRPVY01BWEJF | | HTNRPVY01AZLPI | HTNRPVY01BE33J | | HTNRPVY01BJF25 | | HTNRPVY01ARXWG | | |
| HTNRPVY01BWF3P | | HTNRPVY01AZO4S | HTNRPVY01BE488 | | HTNRPVY01BJFDG | | HTNRPVY01ARYT9 | | |
| HTNRPVY01BWFL9 | | HTNRPVY01AZW6B | HTNRPVY01BE4VD | | HTNRPVY01BJFX6 | | HTNRPVY01AS0DI | | |
| HTNRPVY01BWFVK | | HTNRPVY01AZXS9 | HTNRPVY01BE73I | | HTNRPVY01BJFYY | | HTNRPVY01AS0JG | | |
| HTNRPVY01BWIJN | | HTNRPVY01B02M9 | HTNRPVY01BE7BW | | HTNRPVY01BJIXC | | HTNRPVY01AS0VS | | |
| HTNRPVY01BX0TR | | HTNRPVY01B053X | HTNRPVY01BE7CF | | HTNRPVY01BJLOW | | HTNRPVY01AS6AQ | | |
| HTNRPVY01BX98W | | HTNRPVY01B05FP | HTNRPVY01BE7P4 | | HTNRPVY01BJO3O | | HTNRPVY01AS6WT | | |
| HTNRPVY01BXDAX | | HTNRPVY01B0AGI | HTNRPVY01BECFD | | HTNRPVY01BJOH6 | | HTNRPVY01ASHSL | | |
| HTNRPVY01BXDCL | | HTNRPVY01B0ARZ | HTNRPVY01BEH1C | | HTNRPVY01BJOIA | | HTNRPVY01ASKLE | | |
| HTNRPVY01BXDDB | | HTNRPVY01B0D46 | HTNRPVY01BEK66 | | HTNRPVY01BJU4W | | HTNRPVY01ASKVY | | |
| HTNRPVY01BXJMC | | HTNRPVY01B0FZO | HTNRPVY01BEK6V | | HTNRPVY01BJX4Y | | HTNRPVY01ASTKC | | |
| HTNRPVY01BXJMJ | | HTNRPVY01B0GVT | HTNRPVY01BELXR | | HTNRPVY01BK58Y | | HTNRPVY01ASUCW | | |
| HTNRPVY01BXMTZ | | HTNRPVY01B0JF1 | HTNRPVY01BEOC7 | | HTNRPVY01BKADX | | HTNRPVY01ASW6E | | |
| HTNRPVY01BXS3T | | HTNRPVY01B0KE5 | HTNRPVY01BERIM | | HTNRPVY01BKDM6 | | HTNRPVY01AT14T | | |
| HTNRPVY01BXTYK | | HTNRPVY01B0ME8 | HTNRPVY01BESM7 | | HTNRPVY01BKGP8 | | HTNRPVY01AT80O | | |
| HTNRPVY01BXV9J | | HTNRPVY01B0S6R | HTNRPVY01BEVFJ | | HTNRPVY01BKGRQ | | HTNRPVY01ATB97 | | |
| HTNRPVY01BXWX0 | | HTNRPVY01B0VQZ | HTNRPVY01BEVSV | | HTNRPVY01BKH70 | | HTNRPVY01ATGFM | | |
| HTNRPVY01BXZUI | | HTNRPVY01B0ZG0 | HTNRPVY01BEVYT | | HTNRPVY01BKJ2T | | HTNRPVY01ATLRA | | |
| HTNRPVY01BY2VT | | HTNRPVY01B1FX9 | HTNRPVY01BEYQJ | | HTNRPVY01BKJW9 | | HTNRPVY01ATLXY | | |
| HTNRPVY01BYGOK | | HTNRPVY01B1N6D | HTNRPVY01BF225 | | HTNRPVY01BKKIV | | HTNRPVY01ATU33 | | |
| HTNRPVY01BYGQE | | HTNRPVY01B1UO2 | HTNRPVY01BF52R | | HTNRPVY01BKNW8 | | HTNRPVY01ATWNF | | |
| HTNRPVY01BYMFU | | HTNRPVY01B1VP0 | HTNRPVY01BF6YU | | HTNRPVY01BKOF5 | | HTNRPVY01AU36P | | |
| HTNRPVY01BYOXR | | HTNRPVY01B20I5 | HTNRPVY01BF96B | | HTNRPVY01BKQGK | | HTNRPVY01AU4OL | | |
| HTNRPVY01BYP3H | | HTNRPVY01B226J | HTNRPVY01BFDV9 | | HTNRPVY01BKWIK | | HTNRPVY01AU9O6 | | |
| HTNRPVY01BYS7B | | HTNRPVY01B238V | HTNRPVY01BFEC3 | | HTNRPVY01BKWX7 | | HTNRPVY01AUB2J | | |
| HTNRPVY01BYVKX | | HTNRPVY01B26DP | HTNRPVY01BFGLG | | HTNRPVY01BKZ2H | | HTNRPVY01AUHGI | | |
| HTNRPVY01BYVPC | | HTNRPVY01B2AZP | HTNRPVY01BFGTI | | HTNRPVY01BL4ZZ | | HTNRPVY01AUINJ | | |
| HTNRPVY01BYY1E | | HTNRPVY01B2BIX | HTNRPVY01BFHYJ | | HTNRPVY01BL79O | | HTNRPVY01AULGI | | |
| HTNRPVY01BYY9I | | HTNRPVY01B2BMO | HTNRPVY01BFKCQ | | HTNRPVY01BLC0J | | HTNRPVY01AUOE4 | | |
| HTNRPVY01BYZKX | | HTNRPVY01B2DKM | HTNRPVY01BFKDY | | HTNRPVY01BLCJE | | HTNRPVY01AUOQD | | |
| HTNRPVY01BZ7GQ | | HTNRPVY01B2G1W | HTNRPVY01BFNLN | | HTNRPVY01BLDRJ | | HTNRPVY01AURGB | | |
| HTNRPVY01BZK3W | | HTNRPVY01B2HUU | HTNRPVY01BFRAS | | HTNRPVY01BLFJM | | HTNRPVY01AUUUR | | |
| HTNRPVY01BZOG1 | | HTNRPVY01B2KLF | HTNRPVY01BFUJQ | | HTNRPVY01BLFPQ | | HTNRPVY01AV3BD | | |
| HTNRPVY01BZX3T | | HTNRPVY01B2N7W | HTNRPVY01BFWMC | | HTNRPVY01BLI2J | | HTNRPVY01AV3DB | | |
| HTNRPVY01BPEK4 | | HTNRPVY01BNSTH | HTNRPVY01BQYU0 | | HTNRPVY01BLJAU | | HTNRPVY01BMHMY | | |
| HTNRPVY01BPGXC | | HTNRPVY01BNTTC | HTNRPVY01BR3WA | | HTNRPVY01BLJBD | | HTNRPVY01BMIAD | | |
| HTNRPVY01BPNXB | | HTNRPVY01BNXBU | HTNRPVY01BR737 | | HTNRPVY01BLJCP | | HTNRPVY01BMKWL | | |
| HTNRPVY01BPTTQ | | HTNRPVY01BO1DF | HTNRPVY01BRE1R | | HTNRPVY01BLL08 | | HTNRPVY01BMRSO | | |
| HTNRPVY01BPWVL | | HTNRPVY01BO7R6 | HTNRPVY01BRE71 | | HTNRPVY01BLL0V | | HTNRPVY01BMYA9 | | |
| HTNRPVY01BPX0I | | HTNRPVY01BOC1O | HTNRPVY01BRH7Q | | HTNRPVY01BLL1B | | HTNRPVY01BN2RA | | |
| HTNRPVY01BQ5NV | | HTNRPVY01BOCFC | HTNRPVY01BRK16 | | HTNRPVY01BLPDW | | HTNRPVY01BN2X3 | | |
| HTNRPVY01BQ5TI | | HTNRPVY01BOICL | HTNRPVY01BRLI5 | | HTNRPVY01BLS9O | | HTNRPVY01BN5WI | | |
| HTNRPVY01BQAJ3 | | HTNRPVY01BOJAT | HTNRPVY01BRLU9 | | HTNRPVY01BLSCH | | HTNRPVY01BN6KY | | |
| HTNRPVY01BQGPY | | HTNRPVY01BOL4S | HTNRPVY01BROWZ | | HTNRPVY01BLSPV | | HTNRPVY01BN9AM | | |
| HTNRPVY01BQGWO | | HTNRPVY01BOMQX | HTNRPVY01BRUF7 | | HTNRPVY01BLVIE | | HTNRPVY01BN9U0 | | |
| HTNRPVY01BQJGH | | HTNRPVY01BOOV8 | HTNRPVY01BRXXO | | HTNRPVY01BLVXH | | HTNRPVY01BNHB8 | | |
| HTNRPVY01BQJOS | | HTNRPVY01BOSH0 | HTNRPVY01BRXXZ | | HTNRPVY01BLY5D | | HTNRPVY01BNNUT | | |
| HTNRPVY01BQP0C | | HTNRPVY01BP4HN | HTNRPVY01BS29G | | HTNRPVY01BM0US | | HTNRPVY01BNP4D | | |
| HTNRPVY01BQQL0 | | HTNRPVY01BP581 |  |  | HTNRPVY01BM362 | |  | |  |
| HTNRPVY01BQSVB | | HTNRPVY01BP6AB |  |  | HTNRPVY01BM3T5 | |  | |  |
| HTNRPVY01BQSZF | | HTNRPVY01BP6KO |  |  | HTNRPVY01BM62A | |  | |  |
| HTNRPVY01BQTS6 | | HTNRPVY01BP93Q |  |  | HTNRPVY01BM74L | |  | |  |
| HTNRPVY01BQV6W | | HTNRPVY01BPATQ |  |  | HTNRPVY01BMA56 | |  | |  |
| HTNRPVY01BQVOV | | HTNRPVY01BPBV3 |  |  | HTNRPVY01BMEK1 | |  | |  |
| HTNRPVY01BQWU3 | | HTNRPVY01BPEA2 |  |  | HTNRPVY01BMH24 | |  | |  |
|  | |  |  | |  | | |  |  |
|  | |  |  | |  | | |  |  |
|  | |  |  | |  | | |  |  |
|  | |  |  | |  | | |  |  |
|  | |  |  | |  | | |  |  |
|  | |  |  | |  | | |  |  |
|  | |  |  | |  | | |  |  |
|  | |  |  | |  | | |  |  |
|  | |  |  | |  | | |  |  |
|  | |  |  | |  |  | |  |  |
|  | |  |  | |  |  | |  |  |
|  | |  |  | |  |  | |  |  |
|  | |  |  | |  |  | |  |  |
|  | |  |  | |  |  | |  |  |
|  | |  |  | |  | | |  |  |
|  | |  |  | |  | | |  |  |
|  | |  |  | |  | | |  |  |
|  | |  |  | |  | | |  |  |
|  | |  |  | |  | | |  |  |
|  | |  |  | |  |  | |  |  |
